# Supplementary material for: Study protocol for VIdeo assisted thoracoscopic lobectomy versus conventional Open LobEcTomy for lung cancer, a UK multicentre randomised controlled trial with an internal pilot (the VIOLET study)
Source: BMJ Open. 2019 Oct 14;9(10):e029507. doi: 10.1136/bmjopen-2019-029507 (PMC6797374; doi:10.1136/bmjopen-2019-029507)
Supplement: Author's manuscript [file bmjopen-2019-029507.draft_revisions.pdf]

BMJ Open is committed to open peer review. As part of this commitment we make the peer review history of every article we publish publicly available.

When an article is published we post the peer reviewers' comments and the authors' responses online. We also post the versions of the paper that were used during peer review. These are the versions that the peer review comments apply to.

The versions of the paper that follow are the versions that were submitted during the peer review process. They are not the versions of record or the final published versions. They should not be cited or distributed as the published version of this manuscript.

BMJ Open is an open access journal and the full, final, typeset and author-corrected version of record of the manuscript is available on our site with no access controls, subscription charges or pay-per-view fees (http://bmjopen.bmj.com).

If you have any questions on BMJ Open's open peer review process please email info.bmjopen@bmj.com

BMJ Open

Study Protocol for VIdeo assisted thoracoscopic lobectomy versus conventional Open LobEcTomy for lung cancer, a UK multi-centre randomised controlled trial with an internal pilot (The VIOLET study)

Journal:	*BMJ Open*
Manuscript ID	bmjopen-2019-029507
Article Type:	Protocol
Date Submitted by the Author:	30-Jan-2019

Complete List of Authors:	Lim, Eric; The Royal Brompton Hospital, Academic Division of Thoracic Surgery<br>Batchelor, Tim; Bristol Royal Infirmary, Thoracic Surgery<br>Shackcloth, Michael; Liverpool Heart and Chest Hospital,<br>Dunning, Joel ; The James Cook University Hospital, Marton Road<br>McGonigle, Niall; Harefield Hospital, Royal Brompton and Harefield<br>Brush, Tim; University of Bristol<br>Dabner, Lucy ; University of Bristol<br>Harris, Rosie; University of Bristol<br>Harris, Dawn; University of Bristol<br>Beard, Chloe; University of Bristol<br>Mckeon, Holly; University of Bristol<br>Paramasivan, Sangeetha ; University of Bristol<br>Elliott, Daisy; University of Bristol, School of Social and Community Medicine<br>Realpe, Alba; University of Bristol<br>Stokes, Elizabeth; University of Oxford, Health Economics Research Centre Nuffield Department of Population Health<br>Wordsworth, Sarah; University of Oxford, University of Oxford<br>Blazeby, Jane; University of Bristol, Department of Social Medicine<br>Rogers, Chris; University of Bristol, Clinical Sciences

Keywords:	Randomised controlled trial, minimally invasive, Cardiothoracic surgery < SURGERY, open surgery, video assisted, lung cancer

SCHOLARONE™ Manuscripts

Study Protocol for VIdeo assisted thoracoscopic lobectomy versus conventional Open LobEcTomy for lung cancer, a UK multi-centre randomised controlled trial with an internal pilot (The VIOLET study)

Eric Lim[1]	e.lim@rbht.nhs.uk
Tim Batchelor[2]	tim.batchelor@uhbristol.nhs.uk
Michael Shackcloth[3]	michael.shackcloth@lhch.nhs.uk
Joel Dunning[4]	joeldunning@nhs.net
Niall McGonigle[5]	Niall.McGonigle@belfasttrust.hscni.net
Tim Brush[6]	tim@ebclinical.co.uk
Lucy Dabner[6]	lucy.dabner@bristol.ac.uk
Rosie Harris[6]	rosie.harris@bristol.ac.uk
Dawn Phillips[6]	dawn.phillips@bristol.ac.uk
Chloe Beard[6]	chloe.beard@bristol.ac.uk
Holly McKeon[6]	Holly.mckeon@bristol.ac.uk
Sangeetha Paramasivan[7]	sangeetha.paramasivan@bristol.ac.uk
Daisy Elliott[7]	daisy.elliott@bristol.ac.uk
Alba Realpe Rojas[7]	alba.realpe@bristol.ac.uk
Elizabeth Stokes[8]	elizabeth.stokes@dph.ox.ac.uk
Sarah Wordsworth[8]	sarah.wordsworth@dph.ox.ac.uk
Jane Blazeby[7]	j.m.blazeby@bristol.ac.uk
Chris A Rogers on behalf of The VIOLET Trialists**[6]	chris.rogers@bristol.ac.uk

1. The Royal Brompton and Harefield NHS Foundation Trust, Sydney Street, London, UK
2. Thoracic Surgery, Bristol Royal Infirmary, University Hospitals Bristol, Bristol, UK
3. Liverpool Heart and Chest Hospital, Thomas Drive, Liverpool, UK
4. The James Cook University Hospital, Marton Road, Middlesbrough, UK
5. Harefield Hospital, Royal Brompton and Harefield, UK
6. Clinical Trials and Evaluation Unit, Bristol Trials Centre, Bristol Medical School, University of Bristol, Bristol, UK

For peer review only - http://bmjopen.bmj.com/site/about/guidelines.xhtml

7. Population Health Sciences, Bristol Medical School, University of Bristol, Bristol, UK
8. Health Economics Research Centre, Nuffield Department of Population Health, Old Road Campus, Headington, Oxford, UK

Corresponding author:

Professor Eric Lim, Academic Division of Thoracic Surgery, The Royal Brompton Hospital, Sydney Street, London, UK. Tel: +44 (0)207 351 8591.

Fax: +44 (0)207 351 8560

Email: e.lim@rbht.nhs.uk

Word count: 5169 (excluding title page, abstract, references, figures and tables)

Number of figures: 2

Number of tables: 1

Keywords: Randomised controlled trial; minimally invasive; Cardiothoracic surgery; open surgery; video assisted; lung cancer; lobectomy.

For peer review only

ABSTRACT

Introduction: Lung cancer is a leading cause of cancer deaths worldwide and surgery remains the main treatment for early stage disease. Prior to the introduction of video-assisted thoracoscopic surgery (VATS), lung resection for cancer was undertaken through an open thoracotomy. To date the evidence base supporting the different surgical approaches is based on non-randomised studies, small randomised trials and is focused mainly on short term in- hospital outcomes.

Methods and analysis: The VIOLET study is a UK multicentre parallel group randomised controlled trial (RCT) with blinding of outcome assessors and participants (to hospital discharge) comparing the effectiveness, cost-effectiveness and acceptability of VATS lobectomy versus open lobectomy for treatment of lung cancer. We will test the hypothesis that VATS lobectomy is superior to open lobectomy with respect to self-reported physical function five weeks after randomisation (approximately one month after surgery). Secondary outcomes include assessment of efficacy (hospital stay, pain, proportion and time to uptake of chemotherapy), measures of safety (adverse health events), oncological outcomes (proportion of patients upstaged to pN2 disease and disease-free survival), overall survival, and health related quality of life to 1-year. The QuinteT Recruitment Intervention is integrated into the trial to optimise recruitment.

Ethics and dissemination: This trial has been approved by the UK (Dulwich) National Research Ethics Service Committee London. Findings will be written-up as methodology papers for conference presentation, and publication in peer-reviewed journals. Many aspects of the feasibility work will inform surgical RCTs in general and these will be reported at methodology meetings. We will also link with lung cancer clinical studies groups. The patient and public involvement (PPI) group that works with the Respiratory Biomedical Research Unit at the Brompton Hospital will help identify how we can best publicise the findings.

Trial registration: VIOLET is registered at ISRCTN13472721 (doi 10.1186/ISRCTN13472721)

For peer review only - http://bmjopen.bmj.com/site/about/guidelines.xhtml

Article Summary

Strengths and limitations of this study

- First multicentre randomised trial on this topic
- All surgeons carry out both interventions; the randomisation scheme ensures surgeon balance across the groups to minimise performance bias
- Masking of the incision and evaluation of the success of blinding
- Procedures reflective of UK practice (majority are postero-lateral thoracotomy)
- Surgeon crossovers (i.e. surgeon changes after randomisation) can occur in centres with pooled service provision

INTRODUCTION

Background and objectives

Lung cancer is a leading cause of cancer death worldwide and survival in the United Kingdom (UK) remains amongst the lowest in Europe. Surgery, conventionally undertaken through an open thoracotomy for lung resection, remains the treatment for early stage disease. Since the introduction of minimal access video-assisted thoracoscopic surgery (VATS) techniques, lung cancer resection undertaken through a VATS approach increased from 14% in 2010 to 40% in 2014 in the UK.(1)

Much of the evidence generated to date is based on non-randomised studies(2, 3) or small randomised trials focusing on short term (in-hospital) outcomes(4), that are underpowered to detect differences in longer term outcomes such as survival(5) or have focused solely on operative technique.(6) Currently, the most well-designed randomised controlled trial (RCT) has reported shorter hospital stay and less pain in patients randomised to VATS lobectomy.(7) In this study, all patients received epidural anaesthesia and anterior thoracotomy for open surgery, which is not the current practice for most thoracic surgery centres in the UK.

A well designed and conducted RCT comparing the effectiveness and cost-effectiveness of minimal access and open surgery is needed to inform current UK (NHS) practice, health policy and individual surgeon and patient decision-making.

For peer review only - http://bmjopen.bmj.com/site/about/guidelines.xhtml

The VIOLET study is a UK multicentre pragmatic RCT comparing the effectiveness, cost-effectiveness and acceptability of VATS lobectomy versus open lobectomy for treatment of lung cancer.

Aims and objectives

The VIOLET study will test the hypothesis that VATS lobectomy is superior to open lobectomy with respect to self-reported physical function five weeks after randomisation (approximately one month after surgery).

Specific objectives are to estimate:

A. The difference between groups in the average self-reported physical function at five weeks.

B. The difference between groups with respect to a range of secondary outcomes including assessment of efficacy (hospital stay, pain, proportion and time to uptake of chemotherapy), measures of safety (adverse health events), oncological outcomes (proportion of patients upstaged to pN2 disease and disease-free survival) and overall survival.

C. The cost effectiveness of VATS lobectomy compared to open lobectomy.

METHODS

Trial design

A UK-based multicentre parallel group RCT with blinding of outcome assessors and participants until hospital discharge after surgery. Figures 1 and 2 show the expected patient pathway for both phases of recruitment to the VIOLET study.

Phase 1: The first phase with an integrated qualitative component is necessary to establish the processes for recruitment and consent. This phase is also essential to develop a study manual and a measure of surgical expertise to proceed to phase 2. Phase 1 will be conducted in five centres; Royal Brompton Hospital in London, The University Hospitals Bristol in Bristol, Liverpool Heart and Chest Hospital in Liverpool, The James Cook University Hospital in Middlesbrough and Harefield Hospital in Harefield. These centres are well spread geographically and represent a

For peer review only - http://bmjopen.bmj.com/site/about/guidelines.xhtml

mix of university and NHS trusts that are representative of NHS practice. Progression from pilot to the full trial will be dependent on pre-agreed progression criteria (assessed after 18 months of recruitment):

Specifically:
(a) at least 60% of patients undergoing lobectomy are considered eligible for the trial (if necessary, by revising the eligibility criteria);
(b) at least 50% consent to randomisation after 6 months of recruitment;
(c) less than 5% fail to receive their allocated treatment;
(d) less than 5% lost to follow up, excluding deaths;

Phase 2: This phase will extend the study to up to a further five centres. All centres will use the optimum methods of recruitment established in phase 1 and will follow-up all participants to one year.

Study population

Participating *centres* will only be eligible if they meet all the following eligibility criteria: 1. NHS Trust with an established and accredited lung cancer multi-disciplinary team (MDT); 2. Centre carries out ≥40 VATS lobectomies each year and employs at least one surgeon who has carried out ≥ 50 VATS lobectomies.

Participating *surgeons* will be eligible for the trial if they have performed ≥ 50 VATS lobectomies. Prospective surgeons will be required to submit their activity logs, which will be validated against local audit data from the MDT meetings, prior to acceptance to the trial. Lobectomy via open surgery is currently standard procedure and therefore surgical ability and competence will be assured by Specialist GMC registration.

Patients may enter the study if all the following apply:
1. Adult aged ≥16 years of age
2. Able to give written informed consent, undergoing either
    i. Lobectomy or bilobectomy for treatment of known or suspected primary lung cancer beyond lobar orifice* in TNM8 stage cT1-3 (by size criteria, equivalent

For peer review only - http://bmjopen.bmj.com/site/about/guidelines.xhtml

to TNM7 stage cT1a-2b) or cT3 (by virtue of 2 nodules in the same lobe), N0-1 and M0 or

ii. Undergoing frozen section biopsy with the intention to proceed with lobectomy or bilobectomy if primary lung cancer with a peripheral tumour beyond a lobar orifice* in TNM8 stage cT1-3 (by size criteria, equivalent to TNM7 stage cT1a-2b) or cT3 (by virtue of 2 nodules in the same lobe), N0-1 and M0 is confirmed

3. Disease suitable for both minimal access (VATS lobectomy) and lobectomy via open surgery

*In the case of bilobectomy, the distance for the "lobar" orifice is in reference to the bronchus intermedius

Patients may not enter the study if any of the following apply:

1. Previous malignancy that influences life expectancy
2. Pneumonectomy, segmentectomy or non-anatomic resection (e.g. wedge resection) is planned
3. Patient has a serious concomitant disorder that would compromise patient safety during surgery
4. Planned robotic surgery.

Randomisation

Participants will be randomised in a 1:1 ratio to either VATS lobectomy or open lobectomy. Randomisation will take place through a secure internet-based randomisation system, access to which will be restricted to authorised study personnel. Cohort minimisation (with a random element incorporated) will be used to ensure balance across groups with respect to the surgeon and the allocation will be stratified by centre.

Due to the pragmatic nature of this trial there will inevitably be some variability between surgeons, the surgical teams and the perioperative processes. Such heterogeneity is important as this accurately reflects real clinical practice.

For peer review only - http://bmjopen.bmj.com/site/about/guidelines.xhtml

Randomisation will be performed one week prior to the planned operation date, once eligibility has been confirmed and written consent taken by a research nurse. This will allow sufficient time for operating theatre schedules to be arranged. If there is a change in surgeon after randomisation, the analysis will account for the surgeon responsible for performing the operation and not the surgeon originally allocated to the patient.

Trial interventions

All operations will be undertaken with general anaesthesia and with the patient in the lateral decubitus position.

VATS lobectomy is undertaken through one to four keyhole incisions without rib spreading. The use of 'rib spreading' is prohibited as this is the key intra-operative manoeuvre which disrupts tissues and causes pain (and is used in open surgery). The procedure is performed with videoscopic visualisation without direct vision. The hilar structures are dissected, stapled and divided. Endoscopic ligation of pulmonary arterial branches may be performed. The fissure is completed and the lobe of lung resected. The incisions are closed in layers and may involve muscle, fat and skin layers. This definition of VATS lobectomy is a modification of Cancer and Leukaemia Group B (CALGB) 39802.(8)

Conventional open lobectomy is undertaken through a single incision with or without rib resection and with rib spreading. The operation is performed under direct vision with isolation of the hilar structures (vein, artery and bronchus) which are dissected, ligated and divided in sequence and the lobe of lung resected. The procedures may be undertaken using ligatures, over sewing or with staplers. The thoracotomy is closed in layers starting from pericostal sutures over the ribs, muscle, fat and skin layers.

In both groups, lymph node management is undertaken in accordance with the International Association of the Study of Lung Cancer (IASLC) recommendations where a minimum of six nodes / stations are removed, of which three are from the mediastinum that includes the subcarinal station.(9)

For peer review only - http://bmjopen.bmj.com/site/about/guidelines.xhtml

Because this is a pragmatic trial, adaptations and variation in both procedures (with the exception of the mandated elements outlined above) will be permitted although intra-operative details will be collected, and compliance monitored.

Primary and secondary outcomes

The primary outcome is self-reported physical function measured using the European Organization for Research and Treatment of Cancer Quality of Life Questionnaire-C30 (EORTC QLQ-C30) at 5 weeks post-randomisation. Physical function has been chosen because it is a patient-centred outcome that will reflect the anticipated earlier recovery with VATS lobectomy and has been used in other minimal access surgery trials. The primary endpoint has been chosen to be five weeks (one-month post-surgery) to capture the early benefits of minimal access surgery on recovery. Secondary outcomes have been selected to assess the efficacy of the two approaches.

Secondary outcomes are 1. Time from surgery to hospital discharge; 2. Adverse health events; 3. Proportion and time to uptake of adjuvant treatment; 4. Proportion of patients upstaged to pN2 disease after the procedure; 5. Overall and disease-free survival to one-year; 6. Proportion of patients who undergo complete resection during the procedure; 7. Proportion of patients who experience prolonged incision pain defined as the need for analgesia > 6 weeks after surgery; 8. Generic and disease-specific Health-related quality of life (HRQoL) assessed using the EORTC QLQ-C30, QLQ-LC13 and EQ-5D-5L questionnaires completed at 2 weeks, 5 weeks, 3 months, 6 months and 1-year post-randomisation); 9. Resource use measured for the duration of post-operative hospital stay until discharge, and at 5 weeks, 3 months, 6 months and one-year post-randomisation.

Data Collection

The schedule of data collection for the study is shown in Table 1. Data will be collected on paper and then entered onto a bespoke database. Access to the

For peer review only - http://bmjopen.bmj.com/site/about/guidelines.xhtml

database will be via a secure password-protected web-interface (NHS clinical portal). Study data transferred electronically between the University of Bristol and the NHS will only be transferred via a secure NHS.net network in an encrypted form.

Table 1. Data collection for trial participants who agree to randomisation to VATS lobectomy or open lobectomy

	Pre-randomisation	Post-randomisation								
	Baseline	Day of Surgery	Post-op	2 days post-op	Dis-charge	2 weeks*	5 weeks*	3 months*	6 months*	1 year*
Eligibility	X									
Imaging review (CT / PET-CT¥)	X									
Participant characteristics	X									
Audio recorded consultation	X									
Lobectomy via VATS lobectomy or Open Lobectomy		X								
Intra-operative details		X								
Histopathology staging		X								
Tumour sample for research		X								
Patient Questionnaires										
QLQ-C30	X					X	X	X	X	X
QLQ-LC13	X					X	X	X	X	X
EQ5D	X					X	X	X	X	X
Bang Blinding Index				X	X					
Pain score	X		X	X						
Adverse Events		X					X	X	X	X
Resource use	X	X					X	X	X	X
CT scan of chest & abdomen										X

*Follow-up time-points will be calculated from the date of randomisation.
¥ Review of images available from staging scans performed in accordance with standard practice at participating centres

Blinding of staff and study participants

The operating surgeon and staff responsible for the care of the participant during the operation cannot be blinded to the participants' treatment allocation. However, in order to minimise the risk of bias, attempts will be made to blind the research nurse responsible for the collection of follow-up data. Specifically, randomisation will be

For peer review only

performed by a member of the research team who is not responsible for the collection of follow up data for VIOLET study participants.

Furthermore, efforts will be made to minimise the risk of inadvertent unblinding of the research nurse responsible for data collection during the patient's post-operative stay. To accomplish this, large adhesive dressings will be applied to thorax. These adhesive dressings will be positioned similarly for all participants, regardless of their surgical allocation and will cover both real and potential incision/port locations. The initial adhesive dressings will be applied in the operating theatre by the operating team and these will not be changed until 3 days after surgery (or discharge if discharged before day 3), unless soiling or lack of adherence prompts their premature replacement. Three days after surgery, dressings will be changed by a nurse who is not responsible for conducting the participants' follow-up assessments. Wound cleaning will be performed on all real and potential incision/port locations to promote allocation masking.

Patients who agree to participate in the RCT will not be informed of their treatment allocation until they are discharged from hospital after their operation. In order to ensure that study patients are not unblinded during wound cleaning and dressing change, participants will be asked to turn their head away from the wound site that is being tended to. When participants are considered 'fit-for-discharge', they will be informed of their treatment allocation and advised as to how best to care for their surgical wounds.

The success of blinding will be monitored during each participant's in-hospital stay. Participants will be asked to complete the Bang-blinding Index(10) at 2 days post-operatively and at discharge, but before the treatment allocation is revealed. The research nurse responsible for data collection and follow-up of VIOLET study participants will also be asked to complete the Bang-blinding Index when the participant is ready for discharge and after the participant attends for their 5 week and 1-year follow-up appointments.

For peer review only

Standardisation of post-operative management

As this is a pragmatic RCT, post-operative care and the criteria for drain removal will be in accordance with local practice. However, we have identified two elements of patient care, which require standardisation to minimise the potential for bias, namely pain-control and the criteria by which a participant's medical fitness-for-discharge is assessed.

Standardising the use of analgesia across participating centres is impractical and does not reflect the intended pragmatic nature of the trial, it, would also produce data unrepresentative of real clinical practice. Therefore, each participating centre will prescribe analgesia in accordance with their local protocols. All patients recruited to the RCT at that centre will be given the same analgesia regardless of their treatment allocation (i.e. VATS lobectomy or open lobectomy). Local protocols for the provision of analgesia will be defined by the local Principal Investigator (in collaboration with the local research team) prior to the start of recruitment to the RCT. Analgesia administered throughout the participant's in-hospital stay will be recorded on the trial case report forms (CRFs) and compliance with the pre-defined and centre-specific analgesia protocols will be monitored.

In order to objectively compare the time from surgery to hospital discharge between VATS lobectomy and open lobectomy, the following discharge suitability criteria have been developed. Study participants will be evaluated against these criteria to ensure that they are medically fit-for-discharge:

- Participant has achieved satisfactory mobility
- Pain under control with analgesia
- Satisfactory serum haemoglobin and electrolytes (i.e. does not require intervention)
- Satisfactory chest-x-ray (which will be performed as part of routine clinical care)
- No complications that require further / additional treatment

Participants who are considered medically fit-for-discharge may not necessarily be discharged immediately; in some instances, social and other factors may necessitate extended hospitalisation. The time at which participants are considered medically fit-

For peer review only - http://bmjopen.bmj.com/site/about/guidelines.xhtml

for-discharge and when they are physically discharged from hospital will both be recorded on the trial CRFs.

Sample size calculation

We hypothesise that self-reported physical function (scale 0 – 100, with higher scores indicating better function) five weeks after randomisation for participants undergoing a VATS lobectomy will be superior to the physical function for participants having an open lobectomy, as measured using the EORTC QLQ-C30. The sample size has been chosen to test this hypothesis.

Although the primary endpoint is at 5 weeks post-randomisation self-reported physical function will also be assessed at other time points (baseline, 2 weeks, 3 months, 6 months and 1-year). In estimating the sample size these additional measurements have been taken into account. The power calculation requires the estimation of four parameters, i.e. the effect size that would be considered clinically important, the number of pre and post-surgery measures, and the correlations between pre and post-surgery scores and between repeated post-surgery scores. The effect size was chosen based on the published literature(11), which suggests that an effect size of 0.2 to 0.6 standard deviations equates to a clinically important difference in physical function score of between 5 and 14 points. In the absence of data from which to estimate the correlations between repeated measures we assumed conservative estimates (0.3 between pre and post measures, 0.6 between repeated post measures).

The study size has been set at 398; allowing for a 20% dropout at 1-year, the target sample size is 498 participants. This will provide 90% power to test the hypothesis, assuming an effect size of 0.25 standard deviations in physical function would be clinically important. The calculation based on five post-surgery measures assumes the treatment difference is similar at the five time points.

However, it is anticipated that the difference in physical function may change over time. The calculation based on a single measure shows that the study will have >80% power to detect a difference of 0.25 standard deviations and >90% power to

For peer review only - http://bmjopen.bmj.com/site/about/guidelines.xhtml

detect a difference of 0.3 standard deviations at the primary endpoint where dropout is expected to be less than 5%.

A study in 498 participants will also have 80% power to detect a 1-day difference in length of hospital stay (i.e. median 3 days versus 4 days, hazard ratio 1.3); assuming 2% of patients do not survive to discharge.

Research procedures

Generic and disease-specific HRQoL measures will assess the profiles of VATS and open lobectomy in the early and mid-postoperative phases. The extensively validated EQ-5D-5L will assess generic aspects of HRQoL and will be used in the economic evaluation(12, 13). The EORTC QLQ-C30 is one of the most widely used instruments for assessing HRQoL in patients with cancer and the QLQ-LC13 is the lung cancer module with 13 items that assesses lung cancer–specific symptoms.

Study participants will be asked to complete HRQoL questionnaires at baseline and post-operatively at 2 weeks, 5 weeks, 3 months, 6 months and 1-year post-randomisation. Baseline questionnaires will be administered by the research team at site, whereas the questionnaires completed post-operatively will be administered by the coordinating centre. Participants can choose to receive post-operative questionnaires by post or complete via a secure website.

Patient and Public Involvement (PPI)

The Royal Brompton Hospital Cancer Consortia PPI group were involved from inception and advised on trial design, identification of the choice and timing of the primary outcome, and secondary outcomes that were considered to be important. They were consulted between August 2012 and September 2013. The aim of PPI involvement in VIOLET was to advise on patient-orientated outcomes that matter. The group consists of four patients who have undergone surgery for cancer and one carer. Dr Hall, who is a patient, and a general practitioner by profession, has agreed to sit on the Trial Steering Committee (TSC).

For peer review only - http://bmjopen.bmj.com/site/about/guidelines.xhtml

For peer review only

The PPI group will also be involved in reviewing the content and format of PILs and dissemination of the results of the study.

Integrated QuinteT Recruitment Intervention (QRI)

The VIOLET study will employ an integrated QRI to optimise and sustain recruitment throughout the recruitment period. Surgical RCTs face recruitment challenges including surgeons' limited experience of RCTs, having more confidence in particular procedures and variations in individual practice(14). Furthermore, there is a dearth of robust evidence about effective strategies to improve recruitment in RCTs(15). However, qualitative research can be used to understand recruitment in specific RCTs(16-18) as well as across RCTs(19-21), and has been shown to optimise recruitment and informed consent, thereby contributing to successful recruitment and trial completion(22-34). In order to understand the recruitment process at each centre in real time and investigate the sources of recruitment difficulties, some of the key methods employed(35) will be as follows:

Patient pathway through eligibility and recruitment: A comprehensive process of logging potential trial patients through screening and eligibility phases will be undertaken to provide basic data about the levels of eligibility and recruitment, and identify points at which patients opt in or out of the RCT.

Individual patient equipoise: Individual patient equipoise will be explored using semi-structured in-depth interviews, which will explore patients views on the two procedures, the trial, the acceptability of randomisation between procedures and the factors that influence their decision to participate in the RCT or not. Interviewees will include eligible patients who accept randomisation and eligible patients who decline randomisation. This information will help to determine whether there is sufficient patient equipoise for such a study to be able to recruit in the specified time frame.

Surgeon equipoise: In-depth interviews will also be undertaken with surgeons to explore perceptions and experiences of undertaking both procedures, perceptions of their levels of individual equipoise and the equipoise of their colleagues, commitment to the trial, and views about the likely outcome of the trial.

For peer review only - http://bmjopen.bmj.com/site/about/guidelines.xhtml

Study team: Key members of the Trial Management Group (TMG), including the Chief Investigator (CI) and those closely involved in the design, management, leadership and coordination of the trial will be interviewed and there will be the opportunity to record discussions in the TMG about issues of preference and expertise. These interviews and recorded consultations will permit comparisons to be made to detect preferences unwittingly transmitted during recruitment consultations.

In-depth interviews: In-depth, semi-structured interviews will be conducted and audio-recorded with a purposive sample of staff members involved with aspects of trial design/management and recruitment across centres in phase 1 (and phase 2 where necessary). Patients eligible for recruitment to the RCT may also be interviewed. Across the different groups, interviews will explore participants' perspectives of the trial, the two procedures and acceptability of randomisation between procedures. In addition, recruitment staff (primarily surgeons) interviews will explore their experiences of undertaking both procedures (where appropriate), perceptions of equipoise for themselves and their colleagues, and views on likely outcome of the trial. Interview topic guides will be used to ensure similar topic areas are covered across interviews, while still providing the scope for participants to raise issues of pertinence to them.

Audio recording of recruitment appointments: Face-to-face and telephone consultations of healthcare staff (thoracic surgeons, nurses etc) with potentially eligible patients will be routinely audio recorded across centres to understand the recruitment process at each centre and to identify and investigate the challenges to recruitment. The QRI researcher will listen to and qualitatively analyse the appointments, documenting instances such as unclear, insufficient or imbalanced information provision and unintentional transferring of clinician treatment preferences to patients,

An account of the anonymised findings from all the data will be fed back to the RCT CI , with a plan of action to optimise recruitment developed collaboratively with key stakeholders. The data will be used by the QRI team to provide supportive and confidential individual and group feedback to recruiters to help them to communicate

For peer review only - http://bmjopen.bmj.com/site/about/guidelines.xhtml

equipoise, balance treatment options and explain to patients the benefits and purposes of trial participation, whilst optimising informed consent. Feedback sessions will include comparisons between what clinicians think they say to patients (interview data) and what they actually say to patients (consultation data). Rates of recruitment of eligible patients will be closely monitored against the feedback meetings and it is expected that an improvement will be demonstrated in recruitment over time with experience and training for recruiters (as we have demonstrated is possible in other similar trials(18, 19)).

For peer review only

Economic evaluation

The economic evaluation will compare the costs and effects of VATS lobectomy versus open lobectomy, and will follow established guidelines as set out by the National Institute for Health and Care Excellence(36). The within-trial cost-effectiveness analysis will be undertaken from an NHS and personal social services perspective, with a one-year time horizon from the day of surgery. The primary outcome measure for the economic evaluation will be quality-adjusted life-years (QALYs), estimated using the EuroQol EQ-5D-5L, administered at baseline (pre-randomisation), and five time points post-randomisation (see Table 1). Resource use data collection will be integrated into the trial CRFs for the index admission for items such as duration of surgery, number of staples used, and length of stay; and captured from participants regularly during the one-year follow up (see Table 1) for events such as hospital readmissions, outpatient attendances, and GP or nurse visits in the community.

Statistical analysis plan

The data will be analysed on intention to treat (ITT) and follow CONSORT reporting guidelines (http://www.consort-statement.org/). Randomised participants who are not found to have lung cancer will be included in the primary analysis, but a modified ITT analysis excluding these participants will also be performed. Analyses will be adjusted for centre and for design factors included in the cohort minimisation (e.g. the operating surgeon). As the allocation to VATS or open lobectomy is minimised by surgeon, clustering may occur within the dataset. The structure of the data, i.e.

For peer review only - http://bmjopen.bmj.com/site/about/guidelines.xhtml

nesting of patients by surgeon and centre, will be accounted for in the primary analysis.

Patient reported outcome scores (HRQoL) and will be compared using a mixed regression model, adjusted for baseline measures where appropriate. Changes in treatment effect with time will be assessed by adding a treatment x time interaction to the model and comparing models using a likelihood ratio test. Deaths will be accounted for by modelling HRQoL and survival jointly. Model fit will be assessed and alternative models and / or transformations (e.g. to induce normality) will be explored where appropriate.

Missing items or errors on questionnaire measures will be dealt with according to the scoring manuals or via imputation methods. For other outcomes a complete case analysis will be undertaken if fewer than 5% of cases have missing data, otherwise multiple imputation methods will be considered. Compliance rates will be reported, including the numbers of patients who have withdrawn from the study, have been lost to follow up or died. Causes of death for trial participants will be recorded.

Frequencies of adverse events will be described. Treatment differences will be reported with 95% confidence intervals. In this study of 498 patients we are underpowered to detect differences in survival of less than approximately 20% at 2 years. However, survival rates and 95% confidence intervals will be reported.

One subgroup analysis is planned, comparing pain scores by type of analgesia (paravertebral block vs. intercostal block). This will be tested by adding an analgesia by treatment interaction term to the model. In addition, as an exploratory analysis we will report pain scores within the VATS lobectomy group by number of port sites (single vs multiple port sites), but a formal comparison between the sub-sets of the VATS group is not planned.

The primary analysis will take place when follow-up is complete for all recruited participants. Interim analysis will be decided in discussion with the Data Monitoring and Safety Committee (DMSC). There is no intention to compare any outcomes

For peer review only

between groups after phase 1; the only analyses will be descriptive statistics to summarise recruitment to decide whether the trial satisfies the progression criteria.

Economic Evaluation: For the economic evaluation, unit costs will be derived from nationally published sources and attached to resource use data, and the total costs per participant calculated. Responses to the EQ-5D-5L will be assigned valuations derived from published UK population tariffs(37-39), and combined with survival to calculate QALYs gained per participant. Missing resource use and EQ-5D-5L data will be handled using multiple imputation methods(40). From the average costs and QALYs gained in each trial group, the incremental cost-effectiveness ratio will be derived, producing an incremental cost per QALY gained of VATS lobectomy compared to open lobectomy. Univariate and multivariate sensitivity analyses will assess the impact of varying key parameters in the analysis on baseline cost-effectiveness results. Results will be expressed in terms of a cost-effectiveness acceptability curve, which indicates the likelihood that VATS lobectomy is cost-effective for different levels of willingness to pay for health gain.

Qualitative analysis: Analysis of qualitative data will involve transcribing the audio-recorded consultations, interviews and meetings with consent. The QRI researcher will a) analyse the transcripts and notes thematically using techniques of constant comparison(41) and case study approaches to explore the 'clear obstacles' and 'hidden challenges(19) to recruitment in Violet, and b) employ targeted conversation analysis(16) to focus on areas in the consultations where communication appears to struggle or break down to identify aspects of recruitment that could be improved. Subsets of interview and consultation transcripts will be independently coded by two qualitative researchers, with the coding discussed and any discrepancies resolved, to establish a coding frame that can be applied to other transcripts. Descriptive accounts will summarise key challenges to recruitment. Anonymised findings will be documented and synthesised for presentation to the RCT CI.

Access to study data: Access to the study data will be limited to authorised personnel. Data will be collected and retained in accordance with the UK Data Protection Act 1998. An anonymised dataset will be held for future research as per the National Institute for Health Research (NIHR) contractual arrangements.

For peer review only - http://bmjopen.bmj.com/site/about/guidelines.xhtml

ETHICS

Research ethics approval was granted by the UK (Dulwich) National Research Ethics Service Committee London (reference 14/LO/2129) on 7 January 2015. The trial is managed by the Clinical Trials and Evaluation Unit Bristol (CTEU Bristol) and sponsored by Royal Brompton & Harefield NHS Foundation Trust. Participants have the right to withdraw at any time and if they do withdraw, data collected up until the time of withdrawal will be included in the analyses, unless the participant expresses a wish for their data to be destroyed. Withdrawing patients will be asked at this point if they can be contacted to complete HRQoL questionnaires for an assessment of physical function (primary end point). Participants who choose to withdraw from the study will be treated according to their hospitals' standard procedures.

Changes to the protocol since it was first approved

The number of VATS lobectomies performed for surgeons to be eligible to participate in the VIOLET study was reduced from >50 to >40 to allow more surgeons to participate as there was no evidence to suggest a material difference in outcome. Version 5.0 (dated 13/02/2018) of the protocol is currently in use.

Trial entry criteria by stage were amended following the introduction of the 8th edition of the TNM grading to:

i. Lobectomy or bilobectomy for treatment of known or suspected primary lung cancer beyond lobar orifice* in TNM8 stage cT1-3 (by size criteria, equivalent to TNM7 stage cT1a-2b) or cT3 (by virtue of 2 nodules in the same lobe), N0-1 and M0 or

ii. Undergoing frozen section biopsy with the intention to proceed with lobectomy or bilobectomy if primary lung cancer with a peripheral tumour beyond a lobar orifice* in TNM8 stage cT1-3 (by size criteria, equivalent to TNM7 stage cT1a-2b) or cT3 (by virtue of 2 nodules in the same lobe), N0-1 and M0 is confirmed.

*In the case of bilobectomy, the distance for the "lobar" orifice is in reference to the bronchus intermedius

For peer review only - http://bmjopen.bmj.com/site/about/guidelines.xhtml

The protocol was amended so that the research nurse at the site could obtain questionnaire data during a study visit or telephone call, for those participants who do not return their questionnaire. The relevant regulatory approvals were obtained for amendments to the protocol. Relevant parties (e.g. investigators, trial participants) were informed.

Study status

The actual numbers recruited at 18 months were 160 randomised participants and having received Trial Steering Committee and Funder approval, phase 2 is ongoing and the study is actively recruiting in eight centres. The centres opened in Phase 2 are Heartlands Hospital in Birmingham, John Radcliffe Hospital in Oxford and Castle Hill Hospital in Hull.

The full protocol is available from:

https://www.journalslibrary.nihr.ac.uk/programmes/hta/130403/

AUTHOR CONTRIBUTIONS

EL: Study design, preparation and drafting of protocol and manuscript, Chief Investigator for the trial

CAR: Study design, sample size and statistical analysis plan, drafting of protocol and manuscript

JB: Study design, preparation of study protocol and review of manuscript

SP, ARR & DE: Design of integrated qualitative study, preparation of study protocol, review of manuscript

ES & SW: Study design, preparation of study protocol, design of health economic component, review of manuscript

TB: Study design, preparation of protocol and review of manuscript

MS: Study design, preparation of protocol and review of manuscript

JD: Preparation of protocol and review of manuscript

NMcG: Preparation of protocol and review of manuscript

TBr,DP, LD, HM & CB: Preparation of study protocol.

RH: Statistical analysis plan, review of manuscript.

All authors read and approved the final manuscript.

For peer review only - http://bmjopen.bmj.com/site/about/guidelines.xhtml

DECLARATION OF INTERESTS

EL reports personal fees from Ethicon (Johnson and Johnson), Covidien (Medtronic). There are no other competing interests from the authors.

FUNDING STATEMENT

This project is funded by the NIHR Health Technology Assessment (HTA) Programme (ref 13/04/03). The views expressed are those of the author(s) and not necessarily those of the NHS, the NIHR or the Department of Health and Social Care.

JMB is an NIHR Senior Investigator. JMB, DE and SP are supported by the Medical Research Council (MRC) Hub for Trials Methodology Research ConDuCT-II (Collaboration and Innovation for Difficult Trials in Invasive Procedures) (MR/K025643/1). The funders had no role in the study design, data collection and analysis, decision to publish or preparation of the manuscript.

CR was supported by the British Heart Foundation (BHF) until April 2016.

This study was designed and delivered in collaboration with the Clinical Trials and Evaluation Unit (CTEU), a UKCRC registered clinical trials unit which, as part of the Bristol Trials Centre, is in receipt of NIHR Clinical Trials Unit (CTU) support funding.

The NIHR, MRC and BHF will not be involved in the study management.

ADDITIONAL FIGURES

Figure 1. The trial schema showing the recruitment pathway for Phase 1 (pilot phase) of the VIOLET study

Figure 2. The trial schema showing the recruitment pathway for Phase 2 of the VIOLET study

For peer review only - http://bmjopen.bmj.com/site/about/guidelines.xhtml

LIST OF ABBREVIATIONS

BHF	British Heart Foundation
CALGB	Cancer and Leukemia Group B
CI	Chief Investigator
CONSORT	Consolidated Standards of Reporting Trials
ConDuCT-II	Collaboration and Innovation for Difficult Trials in Invasive Procedures
CTEU	Clinical Trials and Evaluation Unit
DMSC	Data monitoring and safety committee
EORTC QLQ-C30	European Organization for Research and Treatment of Cancer Quality of Life Questionnaire-C30
EORTC QLQ-LC13	European Organisation for Research and Treatment of Cancer Quality of Life Questionnaire Lung Cancer 13
EQ-5D	EuroQoL-5D
GMC	General Medical Council
HRQoL	Health related quality of life
HTA	Health Technology Assessment
MDT	Multi-Disciplinary Team
MRC	Medical Research Council
NIHR	National Institute for Health Research
PIL	Patient information leaflet
PPI	Patient and Public Involvement
QALY	Quality-adjusted life year
QRI	QuinteT Recruitment Intervention
RCT	Randomised controlled trial
REC	Research ethics committee
TSC	Trial Steering Committee
TMG	Trial Management Group
TNM	TNM Classification of Malignant Tumours
UKCRC	The UK Clinical Research Collaboration
VATS	Video-assisted thoracoscopic surgery

For peer review only

ACKNOWLEDGEMENTS

The VIOLET trial is sponsored by The Royal Brompton and Harefield NHS Foundation Trust. The sponsor will be responsible for the oversight of the VIOLET study and to ensure the trial is managed appropriately.

VIOLET is supported by the UK Thoracic Surgery Research Collaborative

****VIOLET Trialists**

Project management team members

Professor Eric Lim, Chief Investigator

For peer review only - http://bmjopen.bmj.com/site/about/guidelines.xhtml

Professor Chris Rogers, Methodological lead and Statistician

Tim Brush, Clinical Trial Coordinator

Lucy Dabner, Clinical Trial Coordinator

Dawn Phillips, Clinical Trial Coordinator

Holly Mckeon, Clinical Trial Coordinator

Chloe Beard, Assistant Trial Coordinator

Rosie Harris, Medical Statistician

Dr Daisy Elliott, Senior Research Associate, QuinteT Research Intervention

Dr Sangeetha Paramasivan, Senior Research Associate, QuinteT Research Intervention

Dr Elizabeth Stokes, Health Economist

Dr Sarah Wordsworth, Health Economist

Professor Jane Blazeby, Methodologist and Surgeon

Professor Andrew Nicholson, Pathologist

Participating sites members phase 1

Royal Brompton Hospital:

Professor Eric Lim, Principal Investigator

Miss Sofina Begum, Consultant Cardiothoracic Surgeon

Paulo De Sousa, Senior Research Nurse

Monica Tavares Barbosa, Research Nurse

Bristol Royal Infirmary:

Mr Tim Batchelor, Principal Investigator

Ms Eveline Internullo, Consultant thoracic Surgeon

Mr Rakesh Krishnadas, Consultant thoracic Surgeon

Mr Gianluca Casali, Consultant thoracic Surgeon

Mr Doug West, Consultant thoracic Surgeon

Karen Bobruk, Research Nurse

Catherine O'Donovan, Research Nurse

Louise Flintoff, Research Nurse

Amelia Lowe, Research Nurse

Joanna Nicklin, Research Nurse

Emma Heron, Research Nurse

For peer review only - http://bmjopen.bmj.com/site/about/guidelines.xhtml

Jo Chambers, Research Nurse

Becky Houlihan, Research Nurse

Laura Beacham, Research Nurse

Heather Hudson, Research Nurse

Katy Tucker, Trial Coordinator

Danielle Davis, Trial Coordinator

Liverpool Heart and Chest Hospital:

Mr Mike Shackcloth, Principal Investigator

Mr Julius Asante-Siaw, Consultant Cardiothoracic Surgeon

Ms Susannah Love, Consultant Cardiothoracic Surgeon

Sarah Feeney, Research Nurse

Lindsey Murphy, Research Nurse

Almudena Duran Rosas, Research Nurse

Andrea Young, Research Nurse

James Cook Hospital:

Mr Joel Dunning, Principal Investigator

Mr Ian Paul, Consultant Cardiothoracic Surgeon

Hyder Latif, Clinical Trial Coordinator

Charlotte Jacobs, Clinical Trial Coordinator

Alison Chilvers, Clinical Trial Coordinator

Edward Stephenson, Research Data Assistant

Martyn Cain, Research Data Assistant

Nazalie Iqbal, Research Data Assistant

Harefield Hospital:

Mr Vladimir Anikin, Principal Investigator

Mr Niall McGonigle, previous Principal Investigator

Claire Prendergast, Research Nurse

Lisa Jones, Research Nurse

Paula Rogers, Research Nurse Manager

For peer review only

<u>Participating sites members phase 2</u>

Birmingham Heartlands Hospital:

Mr Babu Naidu, Principal Investigator

Mr Hazem Fallouh, Consultant Thoracic Surgeon

Mr Luis Hernandez, Consultant Thoracic Surgeon

Mr Maninder Kalkat, Consultant Cardiothoracic Surgeon

Mr Richard Steyn, Consultant Thoracic Surgeon

Nicola Oswald, Thoracic Research Fellow

Amy Kerr, Senior Research Nurse

Charlotte Ferris, Research Nurse

Jo Webb, Research Nurse

Joanne Taylor, Research Nurse

Hollie Bancroft, R&D Biomedical scientist

Salma Kadiri, Research Practitioner

Zara Jalal Senior, Thoracic Research Data Manager

Oxford Radcliffe Hospital:

Miss Elizabeth Belcher, Principal Investigator

Mr Dionisios Stavroulias, Consultant Thoracic Surgeon

Mr Francesco Di Chiara, Consultant Thoracic Surgeon

Kathryn Saunders, Research Nurse

May Havinden-Williams, Research Nurse

Castle Hill Hospital:

Professor Mahmoud Loubani, Principal Investigator

Mr Syed Qadri, Consultant Thoracic Surgeon

Karen Dobbs, Research Nurse

Paul Atkin, Research Nurse

Dominic Fellowes, Research Nurse

Leanne Cox, Clinical Trials Assistant

Edinburgh Royal Infirmary:

Mr Vipin Zamvar, Principal Investigator

Lucy Marshall, Research Nurse

For peer review only

Fiona Strachan, Research Nurse Manager

Stacey Stewart, Research Nurse

Independent Trial Steering Committee members

Professor Ruth Langley (Chair)

Professor Joy Adamson

Mr Ian Hunt

Professor Peter Licht

Dr Arjun Nair

Mr Chris Hall

Mr Mike Cowen (from study start to Jan 2017)

Independent Data Monitoring and Safety Committee members26

Ms Susan Dutton (Chair since May 2017, previously member of the Committee)

Mr Alan Kirk

Professor Keith Kerr

Mr Rajesh Shah

Dr Nagmi Qureshi

Professor Tom Treasure (Chair from study start to March 2017)

REFERENCES

1. The SCTS Thoracic Surgery Audit Group. Society for Cardiothoracic Surgery in Great Britain and Ireland. The Thoracic Surgery Registry Brief Report Audit Years 2011-12 to 2013-14 [Available from: https://scts.org/_userfiles/pages/file/Audit%20and%20Outcomes/3_year_data_summary_2015[1].pdf.
2. Yan TD, Black D, Bannon PG, McCaughan BC. Systematic review and meta-analysis of randomized and nonrandomized trials on safety and efficacy of video-assisted thoracic surgery lobectomy for early-stage non-small-cell lung cancer. J Clin Oncol. 2009;27(15):2553-62.
3. Cao C, Manganas C, Ang SC, Yan TD. A meta-analysis of unmatched and matched patients comparing video-assisted thoracoscopic lobectomy and conventional open lobectomy. Ann Cardiothorac Surg. 2012;1(1):16-23.
4. Kirby TJ, Mack MJ, Landreneau RJ, Rice TW. Lobectomy--video-assisted thoracic surgery versus muscle-sparing thoracotomy. A randomized trial. J Thorac Cardiovasc Surg. 1995;109(5):997-1001; discussion -2.

For peer review only - http://bmjopen.bmj.com/site/about/guidelines.xhtml

5. Sugi K, Kaneda Y, Esato K. Video-assisted thoracoscopic lobectomy achieves a satisfactory long-term prognosis in patients with clinical stage IA lung cancer. World J Surg. 2000;24(1):27-30.
6. Sugiura H, Morikawa T, Kaji M, Sasamura Y, Kondo S, Katoh H. Long-term benefits for the quality of life after video-assisted thoracoscopic lobectomy in patients with lung cancer. Surg Laparosc Endosc Percutan Tech. 1999;9(6):403-8.
7. Bendixen M, Jorgensen OD, Kronborg C, Andersen C, Licht PB. Postoperative pain and quality of life after lobectomy via video-assisted thoracoscopic surgery or anterolateral thoracotomy for early stage lung cancer: a randomised controlled trial. Lancet Oncol. 2016;17(6):836-44.
8. Swanson SJ, Herndon JE, 2nd, D'Amico TA, Demmy TL, McKenna RJ, Jr., Green MR, et al. Video-assisted thoracic surgery lobectomy: report of CALGB 39802--a prospective, multi-institution feasibility study. J Clin Oncol. 2007;25(31):4993-7.
9. Goldstraw P. International Association for the Study of Lung Cancer Staging Manual in Thoracic Oncology. Florida, USA: Editorial Rx Press; 2009.
10. Bang H, Ni L, Davis CE. Assessment of blinding in clinical trials. Control Clin Trials. 2004;25(2):143-56.
11. Dolan P. Modeling valuations for EuroQol health states. Medical Care. 1997;35(11):1095-108.
12. EuroQol Group. EuroQol - a new facility for the measurement of health-related quality of life. Health policy. 1990;16(3):199-208.
13. Herdman M, Gudex C, Lloyd A, Janssen M, Kind P, Parkin D, et al. Development and preliminary testing of the new five-level version of EQ-5D (EQ-5D-5L). Quality of life research : an international journal of quality of life aspects of treatment, care and rehabilitation. 2011;20(10):1727-36.
14. Ergina PL, Cook JA, Blazeby JM, Boutron I, Clavien P-A, Reeves BC, et al. Challenges in evaluating surgical innovation. Lancet. 2009;374(9695):1097-104.
15. Treweek S, Mitchell E, Pitkethly M, Cook J, Kjeldstrom M, Taskila T, et al. Strategies to improve recruitment to randomised controlled trials. Cochrane Database of Systematic Reviews. 2010(1).
16. Donovan JL, Lane JA, Peters TJ, Brindle L, Salter E, Gillatt D, et al. Development of a complex intervention improved randomization and informed consent in a randomized controlled trial. J Clin Epidemiol. 2009;62(1):29-36.
17. Paramasivan S, Rogers CA, Welbourn R, Byrne JP, Salter N, Mahon D, et al. Enabling recruitment success in bariatric surgical trials: pilot phase of the By-Band-Sleeve study. International journal of obesity (2005). 2017;41(11):1654-61.
18. Stein RC, Dunn JA, Bartlett JM, Campbell AF, Marshall A, Hall P, et al. OPTIMA prelim: a randomised feasibility study of personalised care in the treatment of women with early breast cancer. Health Technol Assess. 2016;20(10):xxiii-xxix, 1-201.
19. Donovan JL, Paramasivan S, de Salis I, Toerien M. Clear obstacles and hidden challenges: understanding recruiter perspectives in six pragmatic randomised controlled trials. Trials. 2014;15:5.
20. Mills N, Donovan JL, Wade J, Hamdy FC, Neal DE, Lane JA. Exploring treatment preferences facilitated recruitment to randomized controlled trials. J Clin Epidemiol. 2011;64(10):1127-36.
21. Rooshenas L, Elliott D, Wade J, Jepson M, Paramasivan S, Strong S, et al. Conveying Equipoise during Recruitment for Clinical Trials: Qualitative Synthesis of

For peer review only - http://bmjopen.bmj.com/site/about/guidelines.xhtml

Clinicians' Practices across Six Randomised Controlled Trials. PLoS medicine. 2016;13(10):e1002147.
22. Beard DJ, Rees JL, Cook JA, Rombach I, Cooper C, Merritt N, et al. Arthroscopic subacromial decompression for subacromial shoulder pain (CSAW): a multicentre, pragmatic, parallel group, placebo-controlled, three-group, randomised surgical trial. Lancet. 2017;391(10118):329-38.
23. Hamdy FC, Donovan JL, Lane JA, Mason M, Metcalfe C, Holding P, et al. 10-Year Outcomes after Monitoring, Surgery, or Radiotherapy for Localized Prostate Cancer. N Engl J Med. 2016;375(15):1415-24.
24. Rooshenas L SL, Blazeby JM, Rogers CA, Tilling KM, Husbands S, et al. The Quintet Recruitment Intervention supported five randomized trials to recruit to target: a mixed-methods evaluation. Journal of Clinical Epidemiology 2019(106):108-20.
25. de Salis I, Tomlin Z, Toerien M, Donovan J. Qualitative research to improve RCT recruitment: Issues arising in establishing research collaborations. Contemporary Clinical Trials. 2008;29(5):663-70.
26. de Salis I, Tomlin Z, Toerien M, Donovan J. Using qualitative research methods to improve recruitment to randomized controlled trials: the Quartet study. Journal of Health Services Research & Policy. 2008;13:92-6.
27. Donovan J, Mills N, Smith M, Brindle L, Jacoby A, Peters T, et al. Quality improvement report - Improving design and conduct of randomised trials by embedding them in qualitative research: ProtecT (prostate testing for cancer and treatment) study. British Medical Journal. 2002;325(7367):766-9.
28. Donovan JL, Lane JA, Peters TJ, Brindle L, Salter E, Gillatt D, et al. Development of a complex intervention improved randomization and informed consent in a randomized controlled trial. Journal of Clinical Epidemiology. 2009;62(1):29-36.
29. Donovan JL, Paramasivan S, de Salis I, Toerien M. Clear obstacles and hidden challenges: understanding recruiter perspectives in six pragmatic randomised controlled trials. Trials. 2014;15.
30. Howard L, de Salis I, Tomlin Z, Thornicroft G, Donovan J. Why is recruitment to trials difficult? An investigation into recruitment difficulties in an RCT of supported employment in patients with severe mental illness. Contemporary Clinical Trials. 2009;30(1):40-6.
31. Mills N, Donovan JL, Wade J, Hamdy FC, Neal DE, Lane JA. Exploring treatment preferences facilitated recruitment to randomized controlled trials. Journal of Clinical Epidemiology. 2011;64(10):1127-36.
32. Paramasivan S, Huddart R, Hall E, Lewis R, Birtle A, Donovan JL. Key issues in recruitment to randomised controlled trials with very different interventions: a qualitative investigation of recruitment to the SPARE trial (CRUK/07/011). Trials. 2011;12.
33. Tomlin Z, De Salis I, Toerien M, Donovan J. Patient advocacy and patient centredness in participant recruitment to randomised controlled trials: implications for informed consent. Health Expectations, in press.
34. Wade J, Donovan JL, Lane JA, Neal DE, Hamdy FC. It's not just what you say, it's also how you say it: Opening the 'black box' of informed consent appointments in randomised controlled trials. Social Science & Medicine. 2009;68(11):2018-28.
35. Donovan JL, Rooshenas L, Jepson M, Elliott D, Wade J, Avery K, et al. Optimising recruitment and informed consent in randomised controlled trials: the

For peer review only - http://bmjopen.bmj.com/site/about/guidelines.xhtml

development and implementation of the Quintet Recruitment Intervention (QRI). Trials. 2016;17(1):283.
36. National Institute for Health and Care Excellence. Guide to the Methods of Technology Appraisal 2013. London: National Institute for Health and Care Excellence; 2013.
37. Dolan P. Modeling valuations for EuroQol health states. Med Care. 1997;35(11):1095-108.
38. National Institute for Health and Care Excellence. Position statement on use of the EQ-5D-5L valuation set for England (updated November 2018)2018 09 January 2019. Available from: https://www.nice.org.uk/Media/Default/About/what-we-do/NICE-guidance/NICE-technology-appraisal-guidance/eq5d5l_nice_position_statement.pdf.
39. van Hout B, Janssen MF, Feng YS, Kohlmann T, Busschbach J, Golicki D, et al. Interim scoring for the EQ-5D-5L: mapping the EQ-5D-5L to EQ-5D-3L value sets. Value in health : the journal of the International Society for Pharmacoeconomics and Outcomes Research. 2012;15(5):708-15.
40. White IR, Royston P, Wood AM. Multiple imputation using chained equations: Issues and guidance for practice. Statistics in medicine. 2011;30(4):377-99.
41. Glaser BGS, A.L. The Discovery of Grounded Theory: Strategies for Qualitative Research. Piscataway, NJ, USA: Transaction Publishers; 2009.

For peer review only

For peer review only - http://bmjopen.bmj.com/site/about/guidelines.xhtml

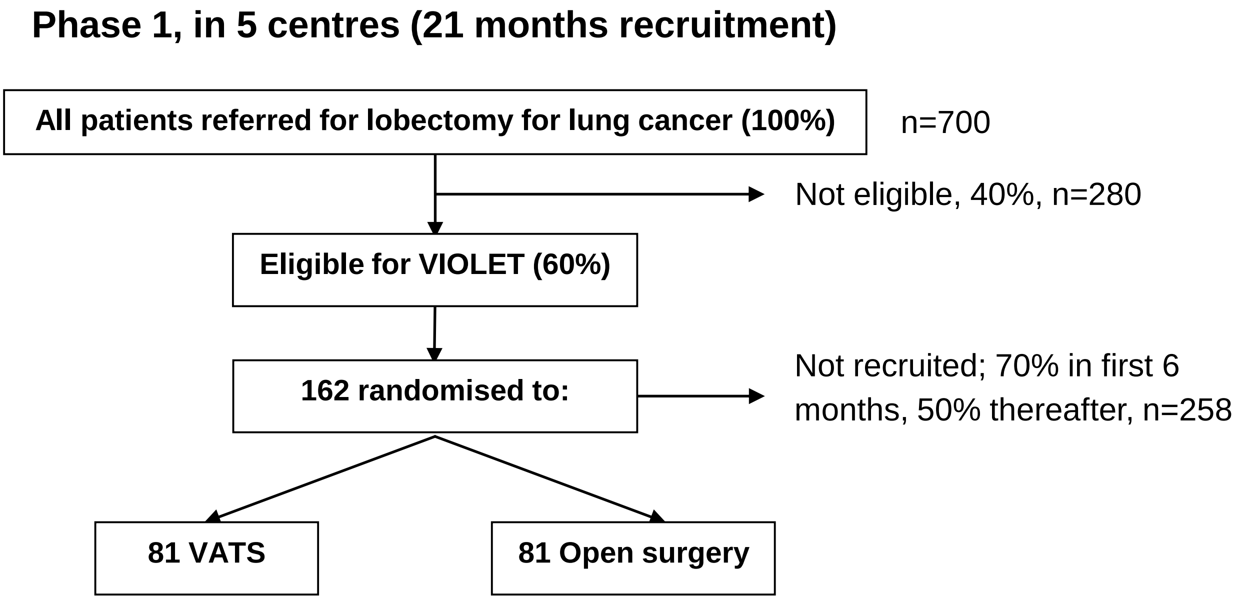

Figure 1: The trial schema for Phase 1 (pilot phase) of the VIOLET study is depicted above

For peer review only - http://bmjopen.bmj.com/site/about/guidelines.xhtml

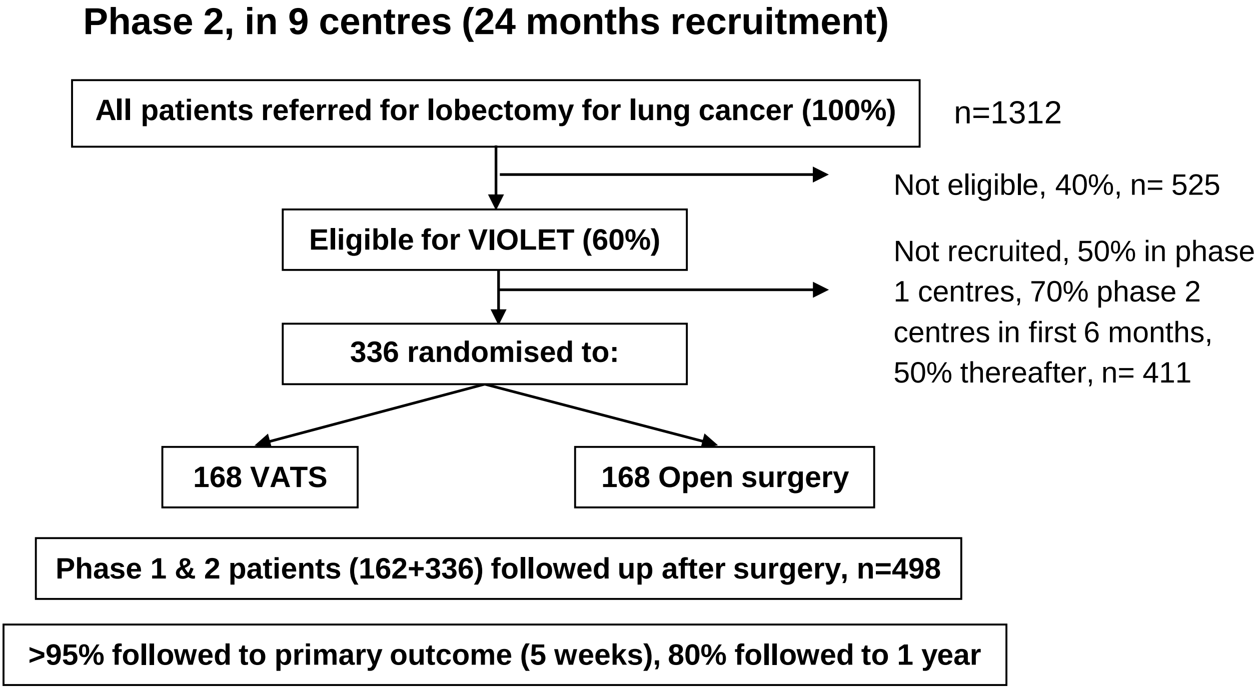

For peer review only - http://bmjopen.bmj.com/site/about/guidelines.xhtml

Figure 2: The trial schema for Phase 2 of the VIOLET study is depicted above

STANDARD PROTOCOL ITEMS: RECOMMENDATIONS FOR INTERVENTIONAL TRIALS

SPIRIT 2013 Checklist: Recommended items to address in a clinical trial protocol and related documents*

Section/item	Item No	Description	Addressed on page number
**Administrative information**			
Title	1	Descriptive title identifying the study design, population, interventions, and, if applicable, trial acronym	1
Trial registration	2a	Trial identifier and registry name. If not yet registered, name of intended registry	3
	2b	All items from the World Health Organization Trial Registration Data Set	N/A
Protocol version	3	Date and version identifier	20
Funding	4	Sources and types of financial, material, and other support	22
Roles and responsibilities	5a	Names, affiliations, and roles of protocol contributors	21
	5b	Name and contact information for the trial sponsor	20
	5c	Role of study sponsor and funders, if any, in study design; collection, management, analysis, and interpretation of data; writing of the report; and the decision to submit the report for publication, including whether they will have ultimate authority over any of these activities	22
	5d	Composition, roles, and responsibilities of the coordinating centre, steering committee, endpoint adjudication committee, data management team, and other individuals or groups overseeing the trial, if applicable (see Item 21a for data monitoring committee)	N/A

For peer review only

**Introduction**			
Background and rationale	6a	Description of research question and justification for undertaking the trial, including summary of relevant studies (published and unpublished) examining benefits and harms for each intervention	4, 5
	6b	Explanation for choice of comparators	5
Objectives	7	Specific objectives or hypotheses	5
Trial design	8	Description of trial design including type of trial (eg, parallel group, crossover, factorial, single group), allocation ratio, and framework (eg, superiority, equivalence, noninferiority, exploratory)	5, 6
**Methods: Participants, interventions, and outcomes**			
Study setting	9	Description of study settings (eg, community clinic, academic hospital) and list of countries where data will be collected. Reference to where list of study sites can be obtained	5, 6
Eligibility criteria	10	Inclusion and exclusion criteria for participants. If applicable, eligibility criteria for study centres and individuals who will perform the interventions (eg, surgeons, psychotherapists)	6, 7
Interventions	11a	Interventions for each group with sufficient detail to allow replication, including how and when they will be administered	8, 9
	11b	Criteria for discontinuing or modifying allocated interventions for a given trial participant (eg, drug dose change in response to harms, participant request, or improving/worsening disease)	8-11
	11c	Strategies to improve adherence to intervention protocols, and any procedures for monitoring adherence (eg, drug tablet return, laboratory tests)	11, 12
	11d	Relevant concomitant care and interventions that are permitted or prohibited during the trial	8-11
Outcomes	12	Primary, secondary, and other outcomes, including the specific measurement variable (eg, systolic blood pressure), analysis metric (eg, change from baseline, final value, time to event), method of aggregation (eg, median, proportion), and time point for each outcome. Explanation of the clinical relevance of chosen efficacy and harm outcomes is strongly recommended	9, 10
Participant timeline	13	Time schedule of enrolment, interventions (including any run-ins and washouts), assessments, and visits for participants. A schematic diagram is highly recommended (see Figure)	9, 10

For peer review only - http://bmjopen.bmj.com/site/about/guidelines.xhtml

Sample size	14	Estimated number of participants needed to achieve study objectives and how it was determined, including clinical and statistical assumptions supporting any sample size calculations	13, 14
Recruitment	15	Strategies for achieving adequate participant enrolment to reach target sample size	14-17
**Methods: Assignment of interventions (for controlled trials)**			
Allocation:			
Sequence generation	16a	Method of generating the allocation sequence (eg, computer-generated random numbers), and list of any factors for stratification. To reduce predictability of a random sequence, details of any planned restriction (eg, blocking) should be provided in a separate document that is unavailable to those who enrol participants or assign interventions	7, 8
Allocation concealment mechanism	16b	Mechanism of implementing the allocation sequence (eg, central telephone; sequentially numbered, opaque, sealed envelopes), describing any steps to conceal the sequence until interventions are assigned	7, 8
Implementation	16c	Who will generate the allocation sequence, who will enrol participants, and who will assign participants to interventions	7, 8
Blinding (masking)	17a	Who will be blinded after assignment to interventions (eg, trial participants, care providers, outcome assessors, data analysts), and how	10, 11
	17b	If blinded, circumstances under which unblinding is permissible, and procedure for revealing a participant's allocated intervention during the trial	10, 11
**Methods: Data collection, management, and analysis**			
Data collection methods	18a	Plans for assessment and collection of outcome, baseline, and other trial data, including any related processes to promote data quality (eg, duplicate measurements, training of assessors) and a description of study instruments (eg, questionnaires, laboratory tests) along with their reliability and validity, if known. Reference to where data collection forms can be found, if not in the protocol	9, 10, 13, 14
	18b	Plans to promote participant retention and complete follow-up, including list of any outcome data to be collected for participants who discontinue or deviate from intervention protocols	14-16, 20

Data management	19	Plans for data entry, coding, security, and storage, including any related processes to promote data quality (eg, double data entry; range checks for data values). Reference to where details of data management procedures can be found, if not in the protocol	N/A
Statistical methods	20a	Statistical methods for analysing primary and secondary outcomes. Reference to where other details of the statistical analysis plan can be found, if not in the protocol	17, 18
	20b	Methods for any additional analyses (eg, subgroup and adjusted analyses)	N/A
	20c	Definition of analysis population relating to protocol non-adherence (eg, as randomised analysis), and any statistical methods to handle missing data (eg, multiple imputation)	18
**Methods: Monitoring**			
Data monitoring	21a	Composition of data monitoring committee (DMC); summary of its role and reporting structure; statement of whether it is independent from the sponsor and competing interests; and reference to where further details about its charter can be found, if not in the protocol. Alternatively, an explanation of why a DMC is not needed	27
	21b	Description of any interim analyses and stopping guidelines, including who will have access to these interim results and make the final decision to terminate the trial	N/A
Harms	22	Plans for collecting, assessing, reporting, and managing solicited and spontaneously reported adverse events and other unintended effects of trial interventions or trial conduct	N/A
Auditing	23	Frequency and procedures for auditing trial conduct, if any, and whether the process will be independent from investigators and the sponsor	N/A
**Ethics and dissemination**			
Research ethics approval	24	Plans for seeking research ethics committee/institutional review board (REC/IRB) approval	20, 21
Protocol amendments	25	Plans for communicating important protocol modifications (eg, changes to eligibility criteria, outcomes, analyses) to relevant parties (eg, investigators, REC/IRBs, trial participants, trial registries, journals, regulators)	3, 15

Consent or assent	26a	Who will obtain informed consent or assent from potential trial participants or authorised surrogates, and how (see Item 32)	7, 8
	26b	Additional consent provisions for collection and use of participant data and biological specimens in ancillary studies, if applicable	N/A
Confidentiality	27	How personal information about potential and enrolled participants will be collected, shared, and maintained in order to protect confidentiality before, during, and after the trial	9, 10
Declaration of interests	28	Financial and other competing interests for principal investigators for the overall trial and each study site	20, 22
Access to data	29	Statement of who will have access to the final trial dataset, and disclosure of contractual agreements that limit such access for investigators	19
Ancillary and post-trial care	30	Provisions, if any, for ancillary and post-trial care, and for compensation to those who suffer harm from trial participation	N/A
Dissemination policy	31a	Plans for investigators and sponsor to communicate trial results to participants, healthcare professionals, the public, and other relevant groups (eg, via publication, reporting in results databases, or other data sharing arrangements), including any publication restrictions	3, 15
	31b	Authorship eligibility guidelines and any intended use of professional writers	N/A
	31c	Plans, if any, for granting public access to the full protocol, participant-level dataset, and statistical code	N/A
**Appendices**			
Informed consent materials	32	Model consent form and other related documentation given to participants and authorised surrogates	On request
Biological specimens	33	Plans for collection, laboratory evaluation, and storage of biological specimens for genetic or molecular analysis in the current trial and for future use in ancillary studies, if applicable	N/A

*It is strongly recommended that this checklist be read in conjunction with the SPIRIT 2013 Explanation & Elaboration for important clarification on the items. Amendments to the protocol should be tracked and dated. The SPIRIT checklist is copyrighted by the SPIRIT Group under the Creative Commons "Attribution-NonCommercial-NoDerivs 3.0 Unported" license.

BMJ Open

Study Protocol for VIdeo assisted thoracoscopic lobectomy versus conventional Open LobEcTomy for lung cancer, a UK multi-centre randomised controlled trial with an internal pilot (The VIOLET study)

Journal:	*BMJ Open*
Manuscript ID	bmjopen-2019-029507.R1
Article Type:	Protocol
Date Submitted by the Author:	20-May-2019
Complete List of Authors:	Lim, Eric; The Royal Brompton Hospital, Academic Division of Thoracic Surgery<br>Batchelor, Tim; Bristol Royal Infirmary, Thoracic Surgery<br>Shackcloth, Michael; Liverpool Heart and Chest Hospital,<br>Dunning, Joel ; The James Cook University Hospital, Marton Road<br>McGonigle, Niall; Harefield Hospital, Royal Brompton and Harefield<br>Brush, Tim; University of Bristol<br>Dabner, Lucy ; University of Bristol<br>Harris, Rosie; University of Bristol<br>Mckeon, Holly; University of Bristol<br>Paramasivan, Sangeetha ; University of Bristol<br>Elliott, Daisy; University of Bristol, School of Social and Community Medicine<br>Stokes, Elizabeth; University of Oxford, Health Economics Research Centre Nuffield Department of Population Health<br>Wordsworth, Sarah; University of Oxford, University of Oxford<br>Blazeby, Jane; University of Bristol, Department of Social Medicine<br>Rogers, Chris; University of Bristol, Clinical Sciences
**Primary Subject Heading**:	Surgery
Secondary Subject Heading:	Oncology, Respiratory medicine
Keywords:	Randomised controlled trial, minimally invasive, Cardiothoracic surgery < SURGERY, open surgery, video assisted, lung cancer

SCHOLARONE™
Manuscripts

Study Protocol for VIdeo assisted thoracoscopic lobectomy versus conventional Open LobEcTomy for lung cancer, a UK multi-centre randomised controlled trial with an internal pilot (The VIOLET study)

Eric Lim[1]	e.lim@rbht.nhs.uk
Tim Batchelor[2]	tim.batchelor@uhbristol.nhs.uk
Michael Shackcloth[3]	michael.shackcloth@lhch.nhs.uk
Joel Dunning[4]	joeldunning@nhs.net
Niall McGonigle[5]	Niall.McGonigle@belfasttrust.hscni.net
Tim Brush[6]	tim@ebclinical.co.uk
Lucy Dabner[6]	lucy.dabner@bristol.ac.uk
Rosie Harris[6]	rosie.harris@bristol.ac.uk
Holly McKeon[6]	Holly.mckeon@bristol.ac.uk
Sangeetha Paramasivan[7]	sangeetha.paramasivan@bristol.ac.uk
Daisy Elliott[7]	daisy.elliott@bristol.ac.uk
Elizabeth Stokes[8]	elizabeth.stokes@dph.ox.ac.uk
Sarah Wordsworth[8]	sarah.wordsworth@dph.ox.ac.uk
Jane Blazeby[7]	j.m.blazeby@bristol.ac.uk
Chris A Rogers on behalf of The VIOLET Trialists**[6]	chris.rogers@bristol.ac.uk

1. The Royal Brompton and Harefield NHS Foundation Trust, Sydney Street, London, UK
2. Thoracic Surgery, Bristol Royal Infirmary, University Hospitals Bristol NHS Foundation Trust, Bristol, UK
3. Liverpool Heart and Chest Hospital, Thomas Drive, Liverpool, UK
4. The James Cook University Hospital, Marton Road, Middlesbrough, UK
5. Harefield Hospital, Royal Brompton and Harefield, UK
6. Clinical Trials and Evaluation Unit, Bristol Trials Centre, Bristol Medical School, University of Bristol, Bristol, UK
7. Population Health Sciences, Bristol Medical School, University of Bristol, Bristol, UK
8. Health Economics Research Centre, Nuffield Department of Population Health, University of Oxford, Oxford, UK

For peer review only - http://bmjopen.bmj.com/site/about/guidelines.xhtml

Corresponding author:

Professor Eric Lim, Academic Division of Thoracic Surgery, The Royal Brompton Hospital, Sydney Street, London, UK. Tel: +44 (0)207 351 8591.

Fax: +44 (0)207 351 8560

Email: e.lim@rbht.nhs.uk

Word count: 5225 (excluding title page, abstract, references, figures and tables)

Number of figures: 2

Number of tables: 1

Keywords: Randomised controlled trial; minimally invasive; Cardiothoracic surgery; open surgery; video assisted; lung cancer; lobectomy.

For peer review only

For peer review only - http://bmjopen.bmj.com/site/about/guidelines.xhtml

ABSTRACT

Introduction: Lung cancer is a leading cause of cancer deaths worldwide and surgery remains the main treatment for early stage disease. Prior to the introduction of video-assisted thoracoscopic surgery (VATS), lung resection for cancer was undertaken through an open thoracotomy. To date the evidence base supporting the different surgical approaches is based on non-randomised studies, small randomised trials and is focused mainly on short term in- hospital outcomes.

Methods and analysis: The VIOLET study is a UK multicentre parallel group randomised controlled trial (RCT) with blinding of outcome assessors and participants (to hospital discharge) comparing the effectiveness, cost-effectiveness and acceptability of VATS lobectomy versus open lobectomy for treatment of lung cancer. We will test the hypothesis that VATS lobectomy is superior to open lobectomy with respect to self-reported physical function five weeks after randomisation (approximately one month after surgery). Secondary outcomes include assessment of efficacy (hospital stay, pain, proportion and time to uptake of chemotherapy), measures of safety (adverse health events), oncological outcomes (proportion of patients upstaged to pN2 disease and disease-free survival), overall survival, and health related quality of life to 1-year. The QuinteT Recruitment Intervention is integrated into the trial to optimise recruitment.

Ethics and dissemination: This trial has been approved by the UK (Dulwich) National Research Ethics Service Committee London. Findings will be written-up as methodology papers for conference presentation, and publication in peer-reviewed journals. Many aspects of the feasibility work will inform surgical RCTs in general and these will be reported at methodology meetings. We will also link with lung cancer clinical studies groups. The patient and public involvement (PPI) group that works with the Respiratory Biomedical Research Unit at the Brompton Hospital will help identify how we can best publicise the findings.

Trial registration: VIOLET is registered at ISRCTN13472721 (doi 10.1186/ISRCTN13472721)

For peer review only - http://bmjopen.bmj.com/site/about/guidelines.xhtml

Article Summary

Strengths and limitations of this study

- First multicentre randomised trial on this topic
- All surgeons carry out both interventions; the randomisation scheme ensures surgeon balance across the groups to minimise performance bias
- Masking of the incision and evaluation of the success of blinding
- Procedures reflective of UK practice (majority are postero-lateral thoracotomy)
- Surgeon crossovers (i.e. surgeon changes after randomisation) can occur in centres with pooled service provision

INTRODUCTION

Background and objectives

Lung cancer is a leading cause of cancer death worldwide and survival in the United Kingdom (UK) remains amongst the lowest in Europe. Surgery, conventionally undertaken through an open thoracotomy for lung resection, remains the treatment for early stage disease. Since the introduction of minimal access video-assisted thoracoscopic surgery (VATS) techniques, lung cancer resection undertaken through a VATS approach increased from 14% in 2010 to 40% in 2014 in the UK.(1)

Much of the evidence generated to date is based on non-randomised studies(2, 3) or small randomised trials focusing on short term (in-hospital) outcomes(4), that are underpowered to detect differences in longer term outcomes such as survival(5) or have focused solely on operative technique.(6) Currently, the most well-designed randomised controlled trial (RCT) by Bendixen et al, reported shorter hospital stay and less pain in patients randomised to VATS lobectomy.(7) In this study, all patients received epidural anaesthesia and anterior thoracotomy for open surgery, which is not the current practice for most thoracic surgery centres in the UK. In contrast, a recent trial by Hao et al from China, published in 2018, reported a similar hospital stay in the VATS and axillary thoracotomy groups.(8) In addition, little high quality randomised data has been published to ascertain the cost effectiveness (i.e. quality of life and costs) for VATS, highlighted in a follow up report by Bendixen et al and an

For peer review only - http://bmjopen.bmj.com/site/about/guidelines.xhtml

on-going trial in France (Lungsco1) that will specifically compare VATS lobectomy versus open thoracotomy from an economic cost to society perspective.(9, 10)

A well designed and conducted RCT comparing the effectiveness and cost-effectiveness of minimal access and open surgery is needed to inform current UK (NHS) practice, health policy and individual surgeon and patient decision-making.

The VIOLET study is a UK multicentre pragmatic RCT comparing the effectiveness, cost-effectiveness and acceptability of VATS lobectomy versus open lobectomy for treatment of lung cancer.

Aims and objectives

The VIOLET study will test the hypothesis that VATS lobectomy is superior to open lobectomy with respect to self-reported physical function five weeks after randomisation (approximately one month after surgery).

Specific objectives are to estimate:

A. The difference between groups in the average self-reported physical function at five weeks.

B. The difference between groups with respect to a range of secondary outcomes including assessment of efficacy (hospital stay, pain, proportion and time to uptake of chemotherapy), measures of safety (adverse health events), oncological outcomes (proportion of patients upstaged to pN2 disease and disease-free survival) and overall survival.

C. The cost effectiveness of VATS lobectomy compared to open lobectomy.

METHODS

Trial design

A UK-based multicentre parallel group RCT with blinding of outcome assessors and participants until hospital discharge after surgery. Figures 1 and 2 show the expected patient pathway for both phases of recruitment to the VIOLET study.

For peer review only - http://bmjopen.bmj.com/site/about/guidelines.xhtml

Phase 1: The first phase with an integrated qualitative component is necessary to establish the processes for recruitment and consent. This phase is also essential to develop a study manual and a measure of surgical expertise to proceed to phase 2. Phase 1 will be conducted in five centres; Royal Brompton Hospital in London, The University Hospitals Bristol in Bristol, Liverpool Heart and Chest Hospital in Liverpool, The James Cook University Hospital in Middlesbrough and Harefield Hospital in Harefield. These centres are well spread geographically and represent a mix of university and NHS trusts that are representative of NHS practice. Progression from pilot to the full trial will be dependent on pre-agreed progression criteria (assessed after 18 months of recruitment):

Specifically:

(a) at least 60% of patients undergoing lobectomy are considered eligible for the trial (if necessary, by revising the eligibility criteria);

(b) at least 50% consent to randomisation after 6 months of recruitment;

(c) less than 5% fail to receive their allocated treatment;

(d) less than 5% lost to follow up, excluding deaths;

Phase 2: This phase will extend the study to up to a further five centres. All centres will use the optimum methods of recruitment established in phase 1 and will follow-up all participants to one year.

Study population

Participating *centres* will only be eligible if they meet all the following eligibility criteria: 1. NHS Trust with an established and accredited lung cancer multi-disciplinary team (MDT); 2. Centre carries out ≥40 VATS lobectomies each year and employs at least one surgeon who has carried out ≥ 50 VATS lobectomies.

Participating *surgeons* will be eligible for the trial if they have performed ≥ 50 VATS lobectomies. Prospective surgeons will be required to submit their activity logs, which will be validated against local audit data from the MDT meetings, prior to acceptance to the trial. Lobectomy via open surgery is currently standard procedure

For peer review only - http://bmjopen.bmj.com/site/about/guidelines.xhtml

and therefore surgical ability and competence will be assured by Specialist GMC registration.

Patients may enter the study if all the following apply:

1. Adult aged ≥16 years of age
2. Able to give written informed consent, undergoing either
    i. Lobectomy or bilobectomy for treatment of known or suspected primary lung cancer beyond lobar orifice* in TNM8 stage cT1-3 (by size criteria, equivalent to TNM7 stage cT1a-2b) or cT3 (by virtue of 2 nodules in the same lobe), N0-1 and M0 or
    ii. Undergoing frozen section biopsy with the intention to proceed with lobectomy or bilobectomy if primary lung cancer with a peripheral tumour beyond a lobar orifice* in TNM8 stage cT1-3 (by size criteria, equivalent to TNM7 stage cT1a-2b) or cT3 (by virtue of 2 nodules in the same lobe), N0-1 and M0 is confirmed
3. Disease suitable for both minimal access (VATS lobectomy) and lobectomy via open surgery

*In the case of bilobectomy, the distance for the “lobar” orifice is in reference to the bronchus intermedius

Patients may not enter the study if any of the following apply:

1. Previous malignancy that influences life expectancy
2. Pneumonectomy, segmentectomy or non-anatomic resection (e.g. wedge resection) is planned
3. Patient has a serious concomitant disorder that would compromise patient safety during surgery
4. Planned robotic surgery.

Randomisation

Participants will be randomised in a 1:1 ratio to either VATS lobectomy or open lobectomy. Randomisation will take place through a secure internet-based randomisation system, access to which will be restricted to authorised study personnel. Cohort minimisation (with a random element incorporated) will be used to

For peer review only - http://bmjopen.bmj.com/site/about/guidelines.xhtml

ensure balance across groups with respect to the surgeon and the allocation will be stratified by centre.

Due to the pragmatic nature of this trial there will inevitably be some variability between surgeons, the surgical teams and the perioperative processes. Such heterogeneity is important as this accurately reflects real clinical practice.

Randomisation will be performed one week prior to the planned operation date, once eligibility has been confirmed and written consent taken by a research nurse. This will allow sufficient time for operating theatre schedules to be arranged. If there is a change in surgeon after randomisation, the analysis will account for the surgeon responsible for performing the operation and not the surgeon originally allocated to the patient.

Trial interventions

All operations will be undertaken with general anaesthesia and with the patient in the lateral decubitus position.

VATS lobectomy is undertaken through one to four keyhole incisions without rib spreading. The use of 'rib spreading' is prohibited as this is the key intra-operative manoeuvre which disrupts tissues and causes pain (and is used in open surgery). The procedure is performed with videoscopic visualisation without direct vision. The hilar structures are dissected, stapled and divided. Endoscopic ligation of pulmonary arterial branches may be performed. The fissure is completed and the lobe of lung resected. The incisions are closed in layers and may involve muscle, fat and skin layers. This definition of VATS lobectomy is a modification of Cancer and Leukaemia Group B (CALGB) 39802.(11)

Conventional open lobectomy is undertaken through a single incision with or without rib resection and with rib spreading. The operation is performed under direct vision with isolation of the hilar structures (vein, artery and bronchus) which are dissected, ligated and divided in sequence and the lobe of lung resected. The procedures may be undertaken using ligatures, over sewing or with staplers. The thoracotomy is

For peer review only - http://bmjopen.bmj.com/site/about/guidelines.xhtml

closed in layers starting from pericostal sutures over the ribs, muscle, fat and skin layers.

In both groups, lymph node management is undertaken in accordance with the International Association of the Study of Lung Cancer (IASLC) recommendations where a minimum of six nodes / stations are removed, of which three are from the mediastinum that includes the subcarinal station.(12)

Because this is a pragmatic trial, adaptations and variation in both procedures (with the exception of the mandated elements outlined above) will be permitted although intra-operative details will be collected, and compliance monitored.

Primary and secondary outcomes

The primary outcome is self-reported physical function measured using the European Organization for Research and Treatment of Cancer Quality of Life Questionnaire-C30 (EORTC QLQ-C30) at 5 weeks post-randomisation. Physical function has been chosen because it is a patient-centred outcome that will reflect the anticipated earlier recovery with VATS lobectomy and has been used in other minimal access surgery trials. The primary endpoint has been chosen to be five weeks (one-month post-surgery) to capture the early benefits of minimal access surgery on recovery. Secondary outcomes have been selected to assess the efficacy of the two approaches.

Secondary outcomes are 1. Time from surgery to hospital discharge; 2. Adverse health events; 3. Proportion and time to uptake of adjuvant treatment; 4. Proportion of patients upstaged to pN2 disease after the procedure; 5. Overall and disease-free survival to one-year; 6. Proportion of patients who undergo complete resection during the procedure; 7. Proportion of patients who experience prolonged incision pain defined as the need for analgesia > 6 weeks after surgery; 8. Generic and disease-specific Health-related quality of life (HRQoL) assessed using the EORTC QLQ-C30, QLQ-LC13 and EQ-5D-5L questionnaires completed at 2 weeks, 5 weeks, 3 months, 6 months and 1-year post-randomisation); 9. Resource use

For peer review only - http://bmjopen.bmj.com/site/about/guidelines.xhtml

measured for the duration of post-operative hospital stay until discharge, and at 5 weeks, 3 months, 6 months and one-year post-randomisation.

Data Collection

The schedule of data collection for the study is shown in Table 1. Data will be collected on paper and then entered onto a bespoke database. Access to the database will be via a secure password-protected web-interface (NHS clinical portal). Study data transferred electronically between the University of Bristol and the NHS will only be transferred via a secure NHS.net network in an encrypted form.

For peer review only

Table 1. Data collection for trial participants who agree to randomisation to VATS lobectomy or open lobectomy

	**Pre-randomisation**	**Post-randomisation**								
	**Baseline**	**Day of Surgery**	**Post-op**	**2 days post-op**	**Dis-charge**	**2 weeks***	**5 weeks***	**3 months***	**6 months***	**1 year***
Eligibility	X									
Imaging review (CT / PET-CT¥)	X									
Participant characteristics	X									
Audio recorded consultation	X									
Lobectomy via VATS lobectomy or Open Lobectomy		X								
Intra-operative details		X								
Histopathology staging		X								
Tumour sample for research		X								
Patient Questionnaires										
QLQ-C30	X					X	X	X	X	X
QLQ-LC13	X					X	X	X	X	X
EQ5D	X					X	X	X	X	X
Bang Blinding Index				X	X					
Pain score	X		X	X						
Adverse Events		X (Day of Surgery to Discharge)					X	X	X	X
Resource use	X	X (Day of Surgery to Discharge)					X	X	X	X
CT scan of chest & abdomen										X

*Follow-up time-points will be calculated from the date of randomisation.
¥ Review of images available from staging scans performed in accordance with standard practice at participating centres

For peer review only - http://bmjopen.bmj.com/site/about/guidelines.xhtml

Blinding of staff and study participants

The operating surgeon and staff responsible for the care of the participant during the operation cannot be blinded to the participants' treatment allocation. However, in order to minimise the risk of bias, attempts will be made to blind the research nurse responsible for the collection of follow-up data. Specifically, randomisation will be performed by a member of the research team who is not responsible for the collection of follow up data for VIOLET study participants.

Furthermore, efforts will be made to minimise the risk of inadvertent unblinding of the research nurse responsible for data collection during the patient's post-operative stay. To accomplish this, large adhesive dressings will be applied to thorax. These adhesive dressings will be positioned similarly for all participants, regardless of their surgical allocation and will cover both real and potential incision/port locations. The initial adhesive dressings will be applied in the operating theatre by the operating team and these will not be changed until 3 days after surgery (or discharge if discharged before day 3), unless soiling or lack of adherence prompts their premature replacement. Three days after surgery, dressings will be changed by a nurse who is not responsible for conducting the participants' follow-up assessments. Wound cleaning will be performed on all real and potential incision/port locations to promote allocation masking.

Patients who agree to participate in the RCT will not be informed of their treatment allocation until they are discharged from hospital after their operation. In order to ensure that study patients are not unblinded during wound cleaning and dressing change, participants will be asked to turn their head away from the wound site that is being tended to. When participants are considered 'fit-for-discharge', they will be informed of their treatment allocation and advised as to how best to care for their surgical wounds.

The success of blinding will be monitored during each participant's in-hospital stay. Participants will be asked to complete the Bang-blinding Index(13) at 2 days post-operatively and at discharge, but before the treatment allocation is revealed. The research nurse responsible for data collection and follow-up of VIOLET study

For peer review only - http://bmjopen.bmj.com/site/about/guidelines.xhtml

participants will also be asked to complete the Bang-blinding Index when the participant is ready for discharge and after the participant attends for their 5 week and 1-year follow-up appointments.

Standardisation of post-operative management

As this is a pragmatic RCT, post-operative care and the criteria for drain removal will be in accordance with local practice. However, we have identified two elements of patient care, which require standardisation to minimise the potential for bias, namely pain-control and the criteria by which a participant's medical fitness-for-discharge is assessed.

Standardising the use of analgesia across participating centres is impractical and does not reflect the intended pragmatic nature of the trial, it, would also produce data unrepresentative of real clinical practice. Therefore, each participating centre will prescribe analgesia in accordance with their local protocols. All patients recruited to the RCT at that centre will be given the same analgesia regardless of their treatment allocation (i.e. VATS lobectomy or open lobectomy). Local protocols for the provision of analgesia will be defined by the local Principal Investigator (in collaboration with the local research team) prior to the start of recruitment to the RCT. Analgesia administered throughout the participant's in-hospital stay will be recorded on the trial case report forms (CRFs) and compliance with the pre-defined and centre-specific analgesia protocols will be monitored.

In order to objectively compare the time from surgery to hospital discharge between VATS lobectomy and open lobectomy, the following discharge suitability criteria have been developed. Study participants will be evaluated against these criteria to ensure that they are medically fit-for-discharge:

• Participant has achieved satisfactory mobility
• Pain under control with analgesia
• Satisfactory serum haemoglobin and electrolytes (i.e. does not require intervention)
• Satisfactory chest-x-ray (which will be performed as part of routine clinical care)
• No complications that require further / additional treatment

For peer review only - http://bmjopen.bmj.com/site/about/guidelines.xhtml

Participants who are considered medically fit-for-discharge may not necessarily be discharged immediately; in some instances, social and other factors may necessitate extended hospitalisation. The time at which participants are considered medically fit-for-discharge and when they are physically discharged from hospital will both be recorded on the trial CRFs.

Sample size calculation

We hypothesise that self-reported physical function (scale 0 – 100, with higher scores indicating better function) five weeks after randomisation for participants undergoing a VATS lobectomy will be superior to the physical function for participants having an open lobectomy, as measured using the EORTC QLQ-C30. The sample size has been chosen to test this hypothesis.

Although the primary endpoint is at 5 weeks post-randomisation self-reported physical function will also be assessed at other time points (baseline, 2 weeks, 3 months, 6 months and 1-year). In estimating the sample size these additional measurements have been taken into account. The power calculation requires the estimation of four parameters, i.e. the effect size that would be considered clinically important, the number of pre and post-surgery measures, and the correlations between pre and post-surgery scores and between repeated post-surgery scores. The effect size was chosen based on the published literature,(14) which suggests that an effect size of 0.2 to 0.6 standard deviations equates to a clinically important difference in physical function score of between 5 and 14 points or approximately a one category change in performance status. In the absence of data from which to estimate the correlations between repeated measures we assumed conservative estimates (0.3 between pre and post measures, 0.6 between repeated post measures).

The study size has been set at 398; allowing for a 20% dropout at 1-year, the target sample size is 498 participants. This will provide 90% power to test the hypothesis, assuming an effect size of 0.25 standard deviations in physical function would be

For peer review only

For peer review only - http://bmjopen.bmj.com/site/about/guidelines.xhtml

clinically important. The calculation based on five post-surgery measures assumes the treatment difference is similar at the five time points.

However, it is anticipated that the difference in physical function may change over time. The calculation based on a single measure shows that the study will have >80% power to detect a difference of 0.25 standard deviations and >90% power to detect a difference of 0.3 standard deviations at the primary endpoint where dropout is expected to be less than 5%.

A study in 498 participants will also have 80% power to detect a 1-day difference in length of hospital stay (i.e. median 3 days versus 4 days, hazard ratio 1.3); assuming 2% of patients do not survive to discharge.

Research procedures

Generic and disease-specific HRQoL measures will assess the profiles of VATS and open lobectomy in the early and mid-postoperative phases. The extensively validated EQ-5D-5L will assess generic aspects of HRQoL and will be used in the economic evaluation.(15, 16) The EORTC QLQ-C30 is one of the most widely used instruments for assessing HRQoL in patients with cancer and the QLQ-LC13 is the lung cancer module with 13 items that assesses lung cancer–specific symptoms.

Study participants will be asked to complete HRQoL questionnaires at baseline and post-operatively at 2 weeks, 5 weeks, 3 months, 6 months and 1-year post-randomisation. Baseline questionnaires will be administered by the research team at site, whereas the questionnaires completed post-operatively will be administered by the coordinating centre. Participants can choose to receive post-operative questionnaires by post or complete via a secure website.

Patient and Public Involvement (PPI)

The Royal Brompton Hospital Cancer Consortia PPI group were involved from inception and advised on trial design, identification of the choice and timing of the primary outcome, and secondary outcomes that were considered to be important.

For peer review only - http://bmjopen.bmj.com/site/about/guidelines.xhtml

For peer review only

They were consulted between August 2012 and September 2013. The aim of PPI involvement in VIOLET was to advise on patient-orientated outcomes that matter. The group consists of four patients who have undergone surgery for cancer and one carer. Dr Hall, who is a patient, and a general practitioner by profession, has agreed to sit on the Trial Steering Committee (TSC).

The PPI group will also be involved in reviewing the content and format of PILs and dissemination of the results of the study.

Integrated QuinteT Recruitment Intervention (QRI)

The VIOLET study will employ an integrated QRI to optimise and sustain recruitment throughout the recruitment period because recruitment is anticipated to be difficult. Although recruitment to RCTs is recognised as a research priority,(17) there is a dearth of robust evidence about effective strategies to improve recruitment in RCTs.(18). Surgical RCTs face specific recruitment challenges due to the complex nature of surgical procedures, the dependence on many healthcare professionals across disciplines and surgeon-related factors such as variations in individual practice/expertise.(19) In addition, surgical RCTs, such as VIOLET, that compare minimally invasive and open operations have historically been difficult to conduct and recruit to.(20, 21)

The QRI, employing primarily qualitative research methods can be used to understand recruitment in specific RCTs(22-24) as well as across RCTs.(25-27) It has been shown to optimise recruitment and informed consent, thereby contributing to successful recruitment and trial completion.(28-30) In VIOLET, in order to understand the recruitment process at each centre in real time, investigate the sources of recruitment difficulties and address the challenges, some of the key methods employed(31) will be as follows:

Patient pathway through eligibility and recruitment: A comprehensive process of logging potential trial patients through screening and eligibility phases will be undertaken to provide basic data about the levels of eligibility and recruitment, and identify points at which patients opt in or out of the RCT.

For peer review only - http://bmjopen.bmj.com/site/about/guidelines.xhtml

In-depth interviews: In-depth, semi-structured interviews will be conducted and audio-recorded with a purposive sample of staff members involved with aspects of trial design/management and recruitment across centres in phase 1 (and phase 2 where necessary). Patients eligible for recruitment to the RCT may also be interviewed. Across the different groups, interviews will explore participants' perspectives of the trial, the two procedures and acceptability of randomisation between procedures. In addition, recruitment staff (primarily surgeons) interviews will explore their experiences of undertaking both procedures (where appropriate), perceptions of equipoise for themselves and their colleagues, and views on likely outcome of the trial. Interview topic guides will be used to ensure similar topic areas are covered across interviews, while still providing the scope for participants to raise issues of pertinence to them.

Audio recording of recruitment appointments: Face-to-face and telephone consultations of healthcare staff (thoracic surgeons, nurses etc) with potentially eligible patients will be routinely audio recorded across centres to understand the recruitment process at each centre and to identify and investigate the challenges to recruitment. The QRI researcher will listen to and qualitatively analyse the appointments, documenting instances such as unclear, insufficient or imbalanced information provision and unintentional transferring of clinician treatment preferences to patients,

An account of the anonymised findings from all the data will be fed back to the RCT CI, with a plan of action to optimise recruitment developed collaboratively with key stakeholders. The data will be used by the QRI team to provide supportive and confidential individual and group feedback to recruiters to help them to communicate equipoise, balance treatment options and explain to patients the benefits and purposes of trial participation, whilst optimising informed consent. Feedback sessions will include comparisons between what clinicians think they say to patients (interview data) and what they actually say to patients (consultation data). Rates of recruitment of eligible patients will be closely monitored against the feedback meetings and it is expected that an improvement will be demonstrated in recruitment

For peer review only - http://bmjopen.bmj.com/site/about/guidelines.xhtml

over time with experience and training for recruiters (as we have demonstrated is possible in other similar trials.(22-24, 28-30))

Economic evaluation

The economic evaluation will compare the costs and effects of VATS lobectomy versus open lobectomy, and will follow established guidelines as set out by the National Institute for Health and Care Excellence(32). The within-trial cost-effectiveness analysis will be undertaken from an NHS and personal social services perspective, with a one-year time horizon from the day of surgery. The primary outcome measure for the economic evaluation will be quality-adjusted life-years (QALYs), estimated using the EuroQol EQ-5D-5L, administered at baseline (pre-randomisation), and five time points post-randomisation (see Table 1). Resource use data collection will be integrated into the trial CRFs for the index admission for items such as duration of surgery, number of staples used, and length of stay; and captured from participants regularly during the one-year follow up (see Table 1) for events such as hospital readmissions, outpatient attendances, and GP or nurse visits in the community.

Statistical analysis plan

The data will be analysed on intention to treat (ITT) and follow CONSORT reporting guidelines (http://www.consort-statement.org/). Randomised participants who are not found to have lung cancer will be included in the primary analysis, but a modified ITT analysis excluding these participants will also be performed. Analyses will be adjusted for centre and for design factors included in the cohort minimisation (e.g. the operating surgeon). As the allocation to VATS or open lobectomy is minimised by surgeon, clustering may occur within the dataset. The structure of the data, i.e. nesting of patients by surgeon and centre, will be accounted for in the primary analysis.

Patient reported outcome scores (HRQoL) and will be compared using a mixed regression model, adjusted for baseline measures where appropriate. Changes in treatment effect with time will be assessed by adding a treatment x time interaction

For peer review only - http://bmjopen.bmj.com/site/about/guidelines.xhtml

to the model and comparing models using a likelihood ratio test. Deaths will be accounted for by modelling HRQoL and survival jointly. Model fit will be assessed and alternative models and / or transformations (e.g. to induce normality) will be explored where appropriate.

Missing items or errors on questionnaire measures will be dealt with according to the scoring manuals or via imputation methods. For other outcomes a complete case analysis will be undertaken if fewer than 5% of cases have missing data, otherwise multiple imputation methods will be considered. Compliance rates will be reported, including the numbers of patients who have withdrawn from the study, have been lost to follow up or died. Causes of death for trial participants will be recorded.

Frequencies of adverse events will be described. Treatment differences will be reported with 95% confidence intervals. In this study of 498 patients we are underpowered to detect differences in survival of less than approximately 20% at 2 years. However, survival rates and 95% confidence intervals will be reported.

One subgroup analysis is planned, comparing pain scores by type of analgesia (paravertebral block vs. intercostal block). This will be tested by adding an analgesia by treatment interaction term to the model. In addition, as an exploratory analysis we will report pain scores within the VATS lobectomy group by number of port sites (single vs multiple port sites), but a formal comparison between the sub-sets of the VATS group is not planned.

The primary analysis will take place when follow-up is complete for all recruited participants. Interim analysis will be decided in discussion with the Data Monitoring and Safety Committee (DMSC). There is no intention to compare any outcomes between groups after phase 1; the only analyses will be descriptive statistics to summarise recruitment to decide whether the trial satisfies the progression criteria.

Economic Evaluation: For the economic evaluation, unit costs will be derived from nationally published sources and attached to resource use data, and the total costs per participant calculated. Responses to the EQ-5D-5L will be assigned valuations derived from published UK population tariffs(33-35), and combined with survival to

For peer review only - http://bmjopen.bmj.com/site/about/guidelines.xhtml

calculate QALYs gained per participant. Missing resource use and EQ-5D-5L data will be handled using multiple imputation methods(36). From the average costs and QALYs gained in each trial group, the incremental cost-effectiveness ratio will be derived, producing an incremental cost per QALY gained of VATS lobectomy compared to open lobectomy. Univariate and multivariate sensitivity analyses will assess the impact of varying key parameters in the analysis on baseline cost-effectiveness results. Results will be expressed in terms of a cost-effectiveness acceptability curve, which indicates the likelihood that VATS lobectomy is cost-effective for different levels of willingness to pay for health gain.

Qualitative analysis: Analysis of qualitative data will involve transcribing the audio-recorded consultations, interviews and meetings with consent. The QRI researcher will a) analyse the transcripts and notes thematically using techniques of constant comparison(37) and case study approaches to explore the 'clear obstacles' and 'hidden challenges(25) to recruitment in Violet, and b) employ targeted conversation analysis(22) to focus on areas in the consultations where communication appears to struggle or break down to identify aspects of recruitment that could be improved. Subsets of interview and consultation transcripts will be independently coded by two qualitative researchers, with the coding discussed and any discrepancies resolved, to establish a coding frame that can be applied to other transcripts. Descriptive accounts will summarise key challenges to recruitment. Anonymised findings will be documented and synthesised for presentation to the RCT CI.

Access to study data: Access to the study data will be limited to authorised personnel. Data will be collected and retained in accordance with the UK Data Protection Act 1998. An anonymised dataset will be held for future research as per the National Institute for Health Research (NIHR) contractual arrangements.

ETHICS

Research ethics approval was granted by the UK (Dulwich) National Research Ethics Service Committee London (reference 14/LO/2129) on 7 January 2015. The trial is managed by the Clinical Trials and Evaluation Unit Bristol (CTEU Bristol) and sponsored by Royal Brompton & Harefield NHS Foundation Trust. Participants have the right to withdraw at any time and if they do withdraw, data collected up until the

For peer review only - http://bmjopen.bmj.com/site/about/guidelines.xhtml

time of withdrawal will be included in the analyses, unless the participant expresses a wish for their data to be destroyed. Withdrawing patients will be asked at this point if they can be contacted to complete HRQoL questionnaires for an assessment of physical function (primary end point). Participants who choose to withdraw from the study will be treated according to their hospitals' standard procedures.

Changes to the protocol since it was first approved

The number of VATS lobectomies performed for surgeons to be eligible to participate in the VIOLET study was reduced from >50 to >40 to allow more surgeons to participate as there was no evidence to suggest a material difference in outcome. Version 5.0 (dated 13/02/2018) of the protocol is currently in use.

Trial entry criteria by stage were amended following the introduction of the 8th edition of the TNM grading to:

i. Lobectomy or bilobectomy for treatment of known or suspected primary lung cancer beyond lobar orifice* in TNM8 stage cT1-3 (by size criteria, equivalent to TNM7 stage cT1a-2b) or cT3 (by virtue of 2 nodules in the same lobe), N0-1 and M0 or

ii. Undergoing frozen section biopsy with the intention to proceed with lobectomy or bilobectomy if primary lung cancer with a peripheral tumour beyond a lobar orifice* in TNM8 stage cT1-3 (by size criteria, equivalent to TNM7 stage cT1a-2b) or cT3 (by virtue of 2 nodules in the same lobe), N0-1 and M0 is confirmed.

*In the case of bilobectomy, the distance for the "lobar" orifice is in reference to the bronchus intermedius

The protocol was amended so that the research nurse at the site could obtain questionnaire data during a study visit or telephone call, for those participants who do not return their questionnaire. The relevant regulatory approvals were obtained for amendments to the protocol. Relevant parties (e.g. investigators, trial participants) were informed.

For peer review only - http://bmjopen.bmj.com/site/about/guidelines.xhtml

Study status

The actual numbers recruited at 18 months were 160 randomised participants and having received Trial Steering Committee and Funder approval, phase 2 is ongoing and the study is actively recruiting in eight centres. The centres opened in Phase 2 are Heartlands Hospital in Birmingham, John Radcliffe Hospital in Oxford and Castle Hill Hospital in Hull.

The full protocol is available from:

https://www.journalslibrary.nihr.ac.uk/programmes/hta/130403/

AUTHOR CONTRIBUTIONS

EL: Study design, preparation and drafting of protocol and manuscript, Chief Investigator for the trial

CAR: Study design, sample size and statistical analysis plan, drafting of protocol and manuscript

JB: Study design, preparation of study protocol and review of manuscript

SP & DE: Design of integrated qualitative study, preparation of study protocol, review of manuscript

ES & SW: Study design, preparation of study protocol, design of health economic component, review of manuscript

TB: Study design, preparation of protocol and review of manuscript

MS: Study design, preparation of protocol and review of manuscript

JD: Preparation of protocol and review of manuscript

NMcG: Preparation of protocol and review of manuscript

TBr, LD, HM: Preparation of study protocol.

RH: Statistical analysis plan, review of manuscript.

All authors read and approved the final manuscript.

DECLARATION OF INTERESTS

EL and TB report personal fees from Ethicon (Johnson and Johnson), Covidien (Medtronic). There are no other competing interests from the authors.

For peer review only

FUNDING STATEMENT

This project is funded by the NIHR Health Technology Assessment (HTA) Programme (ref 13/04/03). The views expressed are those of the author(s) and not necessarily those of the NHS, the NIHR or the Department of Health and Social Care.

JMB is an NIHR Senior Investigator. JMB, DE and SP are supported by the Medical Research Council (MRC) Hub for Trials Methodology Research ConDuCT-II (Collaboration and Innovation for Difficult Trials in Invasive Procedures) (MR/K025643/1). JMB and DE are also supported by the NIHR Bristol Biomedical Research Centre. The funders had no role in the study design, data collection and analysis, decision to publish or preparation of the manuscript.

CR was supported by the British Heart Foundation (BHF) until April 2016.

This study was designed and delivered in collaboration with the Clinical Trials and Evaluation Unit (CTEU), a UKCRC registered clinical trials unit which, as part of the Bristol Trials Centre, is in receipt of NIHR Clinical Trials Unit (CTU) support funding.

The NIHR, MRC and BHF will not be involved in the study management.

ADDITIONAL FIGURES

Figure 1. The trial schema showing the recruitment pathway for Phase 1 (pilot phase) of the VIOLET study

Figure 2. The trial schema showing the recruitment pathway for Phase 2 of the VIOLET study

LIST OF ABBREVIATIONS

BHF	British Heart Foundation
CALGB	Cancer and Leukemia Group B
CI	Chief Investigator
CONSORT	Consolidated Standards of Reporting Trials
ConDuCT-II	Collaboration and Innovation for Difficult Trials in Invasive Procedures
CTEU	Clinical Trials and Evaluation Unit

For peer review only - http://bmjopen.bmj.com/site/about/guidelines.xhtml

DMSC	Data monitoring and safety committee
EORTC QLQ-C30	European Organization for Research and Treatment of Cancer Quality of Life Questionnaire-C30
EORTC QLQ-LC13	European Organisation for Research and Treatment of Cancer Quality of Life Questionnaire Lung Cancer 13
EQ-5D	EuroQoL-5D
GMC	General Medical Council
HRQoL	Health related quality of life
HTA	Health Technology Assessment
MDT	Multi-Disciplinary Team
MRC	Medical Research Council
NIHR	National Institute for Health Research
PIL	Patient information leaflet
PPI	Patient and Public Involvement
QALY	Quality-adjusted life year
QRI	QuinteT Recruitment Intervention
RCT	Randomised controlled trial
REC	Research ethics committee
TSC	Trial Steering Committee
TMG	Trial Management Group
TNM	TNM Classification of Malignant Tumours
UKCRC	The UK Clinical Research Collaboration
VATS	Video-assisted thoracoscopic surgery

ACKNOWLEDGEMENTS

The VIOLET trial is sponsored by The Royal Brompton and Harefield NHS Foundation Trust. The sponsor will be responsible for the oversight of the VIOLET study and to ensure the trial is managed appropriately.

VIOLET is supported by the UK Thoracic Surgery Research Collaborative

****VIOLET Trialists**

Project management team members

Professor Eric Lim, Chief Investigator

Professor Chris Rogers, Methodological lead and Statistician

Tim Brush, Clinical Trial Coordinator

Lucy Dabner, Clinical Trial Coordinator

Dawn Phillips, Clinical Trial Coordinator

Holly Mckeon, Clinical Trial Coordinator

Chloe Beard, Assistant Trial Coordinator

For peer review only

For peer review only - http://bmjopen.bmj.com/site/about/guidelines.xhtml

Rosie Harris, Medical Statistician

Dr Daisy Elliott, Senior Research Associate, QuinteT Research Intervention

Dr Sangeetha Paramasivan, Senior Research Associate, QuinteT Research Intervention

Dr Alba Realpe Rojas, Senior Research Associate, QuinteT Research Intervention

Dr Sarah Wordsworth, Lead Health Economist

Dr Elizabeth Stokes, Health Economist

Professor Jane Blazeby, Methodologist and Surgeon

Professor Andrew G Nicholson, Pathologist

For peer review only

Participating sites members phase 1

Royal Brompton Hospital:

Professor Eric Lim, Principal Investigator, Consultant Thoracic Surgeon

Miss Sofina Begum, Consultant Thoracic Surgeon

Mr Simon Jordan, Consultant Thoracic Surgeon

Paulo De Sousa, Senior Research Nurse

Monica Tavares Barbosa, Research Nurse

Bristol Royal Infirmary:

Mr Tim Batchelor, Principal Investigator, Consultant Thoracic Surgeon

Ms Eveline Internullo, Consultant Thoracic Surgeon

Mr Rakesh Krishnadas, Consultant Thoracic Surgeon

Mr Gianluca Casali, Consultant Thoracic Surgeon

Mr Doug West, Consultant Thoracic Surgeon

Karen Bobruk, Research Nurse

Catherine O'Donovan, Research Nurse

Louise Flintoff, Research Nurse

Amelia Lowe, Trial Coordinator

Joanna Nicklin, Research Nurse

Emma Heron, Research Nurse

Jo Chambers, Research Nurse

Becky Houlihan, Research Nurse

Laura Beacham, Research Nurse

Heather Hudson, Research Nurse

For peer review only - http://bmjopen.bmj.com/site/about/guidelines.xhtml

Katy Tucker, Trial Coordinator

Toni Farmery, Trial Coordinator

Danielle Davis, Trial Coordinator

Liverpool Heart and Chest Hospital:

Mr Mike Shackcloth, Principal Investigator, Consultant Thoracic Surgeon

Mr Julius Asante-Siaw, Consultant Thoracic Surgeon

Ms Susannah Love, Consultant Thoracic Surgeon

Sarah Feeney, Research Nurse

Lindsey Murphy, Research Nurse

Almudena Duran Rosas, Research Nurse

Andrea Young, Research Nurse

James Cook Hospital:

Mr Joel Dunning, Principal Investigator, Consultant Thoracic Surgeon

Mr Ian Paul, Consultant Thoracic Surgeon

Hyder Latif, Clinical Trial Coordinator

Charlotte Jacobs, Clinical Trial Coordinator

Alison Chilvers, Clinical Trial Coordinator

Edward Stephenson, Research Data Assistant

Martyn Cain, Research Data Assistant

Nazalie Iqbal, Research Data Assistant

Harefield Hospital:

Mr Vladimir Anikin, Principal Investigator, Consultant Thoracic Surgeon

Mr Niall McGonigle, previous Principal Investigator, Consultant Thoracic Surgeon

Claire Prendergast, Research Nurse

Lisa Jones, Research Nurse

Paula Rogers, Research Nurse Manager

<u>Participating sites members phase 2</u>

Birmingham Heartlands Hospital:

Mr Babu Naidu, Principal Investigator, Consultant Thoracic Surgeon

Mr Hazem Fallouh, Consultant Thoracic Surgeon

For peer review only - http://bmjopen.bmj.com/site/about/guidelines.xhtml

Mr Luis Hernandez, Consultant Thoracic Surgeon
Mr Maninder Kalkat, Consultant Thoracic Surgeon
Mr Richard Steyn, Consultant Thoracic Surgeon
Nicola Oswald, Thoracic Research Fellow
Amy Kerr, Senior Research Nurse
Charlotte Ferris, Research Nurse
Jo Webb, Research Nurse
Joanne Taylor, Research Nurse
Hollie Bancroft, R&D Biomedical scientist
Salma Kadiri, Research Practitioner
Zara Jalal Senior, Thoracic Research Data Manager

Oxford University Hospitals NHS Foundation Trust:
Miss Elizabeth Belcher, Principal Investigator, Consultant Thoracic Surgeon
Mr Dionisios Stavroulias, Consultant Thoracic Surgeon
Mr Francesco Di Chiara, Consultant Thoracic Surgeon
Kathryn Saunders, Research Nurse
May Havinden-Williams, Research Nurse
Mark Ainsworth, Research Nurse

Castle Hill Hospital:
Professor Mahmoud Loubani, Principal Investigator, Consultant Cardiothoracic Surgeon
Mr Syed Qadri, Consultant Thoracic Surgeon
Karen Dobbs, Research Nurse
Paul Atkin, Research Nurse
Dominic Fellowes, Research Nurse
Leanne Cox, Clinical Trials Assistant

Edinburgh Royal Infirmary:
Mr Vipin Zamvar, Principal Investigator, Consultant Cardiothoracic Surgeon
Lucy Marshall, Research Nurse
Fiona Strachan, Research Nurse Manager
Stacey Stewart, Research Nurse

For peer review only

Independent Trial Steering Committee members

Professor Ruth Langley (Chair), Professor of Oncology and Clinical Trials

Professor Joy Adamson, Professor of Applied Health Research & Ageing

Mr Ian Hunt, Consultant Thoracic Surgeon

Professor Peter Licht, Professor of Cardiothoracic Surgery

Dr Arjun Nair, Consultant Radiologist

Mr Chris Hall, Patient representative

Mr Mike Cowen, Consultant Cardiothoracic Surgeon (from study start to Jan 2017)

Independent Data Monitoring and Safety Committee members

Ms Susan J Dutton (Chair since May 2017, previously member of the Committee), University Research Lecturer and Oxford Clinical Trials Research Unit (OCTRU) Lead Statistician

Mr Alan Kirk, Consultant Thoracic Surgeon

Professor Keith Kerr, Professor of Pulmonary Pathology

Mr Rajesh Shah, Consultant Thoracic Surgeon

Dr Nagmi Qureshi, Consultant Radiologist

Professor Tom Treasure, Professor of Cardiothoracic Surgery (Chair from study start to March 2017)

For peer review only

REFERENCES

1. The SCTS Thoracic Surgery Audit Group. Society for Cardiothoracic Surgery in Great Britain and Ireland. The Thoracic Surgery Registry Brief Report Audit Years 2011-12 to 2013-14 [Available from: https://scts.org/_userfiles/pages/file/Audit%20and%20Outcomes/3_year_data_summary_2015[1].pdf.
2. Yan TD, Black D, Bannon PG, et al. Systematic review and meta-analysis of randomized and nonrandomized trials on safety and efficacy of video-assisted thoracic surgery lobectomy for early-stage non-small-cell lung cancer. J Clin Oncol. 2009;27(15):2553-62.
3. Cao C, Manganas C, Ang SC, et al. A meta-analysis of unmatched and matched patients comparing video-assisted thoracoscopic lobectomy and conventional open lobectomy. Ann Cardiothorac Surg. 2012;1(1):16-23.
4. Kirby TJ, Mack MJ, Landreneau RJ, et al. Lobectomy--video-assisted thoracic surgery versus muscle-sparing thoracotomy. A randomized trial. J Thorac Cardiovasc Surg. 1995;109(5):997-1001; discussion -2.
5. Sugi K, Kaneda Y, Esato K. Video-assisted thoracoscopic lobectomy achieves a satisfactory long-term prognosis in patients with clinical stage IA lung cancer. World J Surg. 2000;24(1):27-30.
6. Sugiura H, Morikawa T, Kaji M, et al. Long-term benefits for the quality of life after video-assisted thoracoscopic lobectomy in patients with lung cancer. Surg Laparosc Endosc Percutan Tech. 1999;9(6):403-8.
7. Bendixen M, Jorgensen OD, Kronborg C, et al. Postoperative pain and quality of life after lobectomy via video-assisted thoracoscopic surgery or anterolateral thoracotomy for early stage lung cancer: a randomised controlled trial. Lancet Oncol. 2016;17(6):836-44.
8. Long H, Tan Q, Luo Q, et al. Thoracoscopic Surgery Versus Thoracotomy for Lung Cancer: Short-Term Outcomes of a Randomized Trial. Ann Thorac Surg. 2018;105(2):386-92.
9. Bendixen M, Kronborg C, Jorgensen OD, et al. Cost-utility analysis of minimally invasive surgery for lung cancer: a randomized controlled trial. Eur J Cardiothorac Surg. 2019.
10. Pagès PB, Abou Hanna H, Bertaux AC, et al. Medicoeconomic analysis of lobectomy using thoracoscopy versus thoracotomy for lung cancer: a study protocol for a multicentre randomised controlled trial (Lungsco01). BMJ Open. 2017;7(6):e012963.
11. Swanson SJ, Herndon JE, 2nd, D'Amico TA, et al. Video-assisted thoracic surgery lobectomy: report of CALGB 39802--a prospective, multi-institution feasibility study. J Clin Oncol. 2007;25(31):4993-7.
12. Goldstraw P. International Association for the Study of Lung Cancer Staging Manual in Thoracic Oncology. Florida, USA: Editorial Rx Press; 2009.
13. Bang H, Ni L, Davis CE. Assessment of blinding in clinical trials. Control Clin Trials. 2004;25(2):143-56.
14. Maringwa JT, Quinten C, King M, et al. Minimal important differences for interpreting health-related quality of life scores from the EORTC QLQ-C30 in lung cancer patients participating in randomized controlled trials. Support Care Cancer. 2011;19(11):1753-60.
15. EuroQol Group. EuroQol - a new facility for the measurement of health-related quality of life. Health policy. 1990;16(3):199-208.

For peer review only - http://bmjopen.bmj.com/site/about/guidelines.xhtml

16. Herdman M, Gudex C, Lloyd A, et al. Development and preliminary testing of the new five-level version of EQ-5D (EQ-5D-5L). Quality of life research : an international journal of quality of life aspects of treatment, care and rehabilitation. 2011;20(10):1727-36.
17. Healy P, Galvin S, Williamson PR, et al. Identifying trial recruitment uncertainties using a James Lind Alliance Priority Setting Partnership - the PRioRiTy (Prioritising Recruitment in Randomised Trials) study. Trials. 2018;19(1):147.
18. Treweek S, Mitchell E, Pitkethly M, et al. Strategies to improve recruitment to randomised controlled trials. Cochrane Database of Systematic Reviews. 2010(1).
19. Ergina PL, Cook JA, Blazeby JM, et al. Challenges in evaluating surgical innovation. Lancet. 2009;374(9695):1097-104.
20. Frampton SK, R.L. John Wickham's New Surgery: 'Minimally Invasive Therapy', Innovation, and Approaches to Medical Practice in Twentieth-century Britain. Soc Hist Med. 2017;30(3):544–66.
21. Schlich TT, C.L. Patient choice and the history of minimally invasive surgery. Lancet. 2016;388:1369-70.
22. Donovan JL, Lane JA, Peters TJ, et al. Development of a complex intervention improved randomization and informed consent in a randomized controlled trial. J Clin Epidemiol. 2009;62(1):29-36.
23. Paramasivan S, Rogers CA, Welbourn R, et al. Enabling recruitment success in bariatric surgical trials: pilot phase of the By-Band-Sleeve study. International journal of obesity (2005). 2017;41(11):1654-61.
24. Stein RC, Dunn JA, Bartlett JM, et al. OPTIMA prelim: a randomised feasibility study of personalised care in the treatment of women with early breast cancer. Health Technol Assess. 2016;20(10):xxiii-xxix, 1-201.
25. Donovan JL, Paramasivan S, de Salis I, et al. Clear obstacles and hidden challenges: understanding recruiter perspectives in six pragmatic randomised controlled trials. Trials. 2014;15:5.
26. Mills N, Donovan JL, Wade J, et al. Exploring treatment preferences facilitated recruitment to randomized controlled trials. J Clin Epidemiol. 2011;64(10):1127-36.
27. Rooshenas L, Elliott D, Wade J, et al. Conveying Equipoise during Recruitment for Clinical Trials: Qualitative Synthesis of Clinicians' Practices across Six Randomised Controlled Trials. PLoS Med. 2016;13(10):e1002147.
28. Beard DJ, Rees JL, Cook JA, et al. Arthroscopic subacromial decompression for subacromial shoulder pain (CSAW): a multicentre, pragmatic, parallel group, placebo-controlled, three-group, randomised surgical trial. Lancet. 2017;391(10118):329-38.
29. Hamdy FC, Donovan JL, Lane JA, et al. 10-Year Outcomes after Monitoring, Surgery, or Radiotherapy for Localized Prostate Cancer. N Engl J Med. 2016;375(15):1415-24.
30. Rooshenas L SL, Blazeby JM, Rogers CA, Tilling KM, Husbands S, et al. The Quintet Recruitment Intervention supported five randomized trials to recruit to target: a mixed-methods evaluation. Journal of Clinical Epidemiology 2019(106):108-20.
31. Donovan JL, Rooshenas L, Jepson M, et al. Optimising recruitment and informed consent in randomised controlled trials: the development and implementation of the Quintet Recruitment Intervention (QRI). Trials. 2016;17(1):283.
32. National Institute for Health and Care Excellence. Guide to the Methods of Technology Appraisal 2013. London: National Institute for Health and Care Excellence; 2013.

For peer review only - http://bmjopen.bmj.com/site/about/guidelines.xhtml

33. Dolan P. Modeling valuations for EuroQol health states. Med Care. 1997;35(11):1095-108.
34. National Institute for Health and Care Excellence. Position statement on use of the EQ-5D-5L valuation set for England (updated November 2018)2018 09 January 2019. Available from: https://www.nice.org.uk/Media/Default/About/what-we-do/NICE-guidance/NICE-technology-appraisal-guidance/eq5d5l_nice_position_statement.pdf.
35. van Hout B, Janssen MF, Feng YS, et al. Interim scoring for the EQ-5D-5L: mapping the EQ-5D-5L to EQ-5D-3L value sets. Value in health : the journal of the International Society for Pharmacoeconomics and Outcomes Research. 2012;15(5):708-15.
36. White IR, Royston P, Wood AM. Multiple imputation using chained equations: Issues and guidance for practice. Statistics in medicine. 2011;30(4):377-99.
37. Glaser BGS, A.L. The Discovery of Grounded Theory: Strategies for Qualitative Research. Piscataway, NJ, USA: Transaction Publishers; 2009.

For peer review only

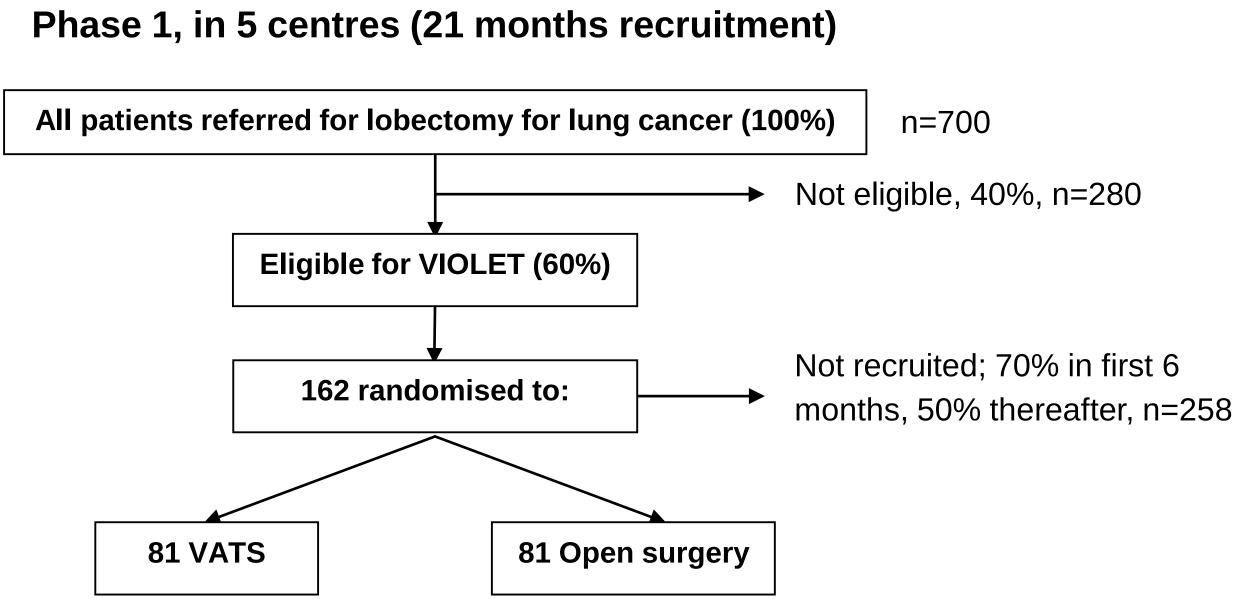

Figure 1: The trial schema for Phase 1 (pilot phase) of the VIOLET study is depicted above

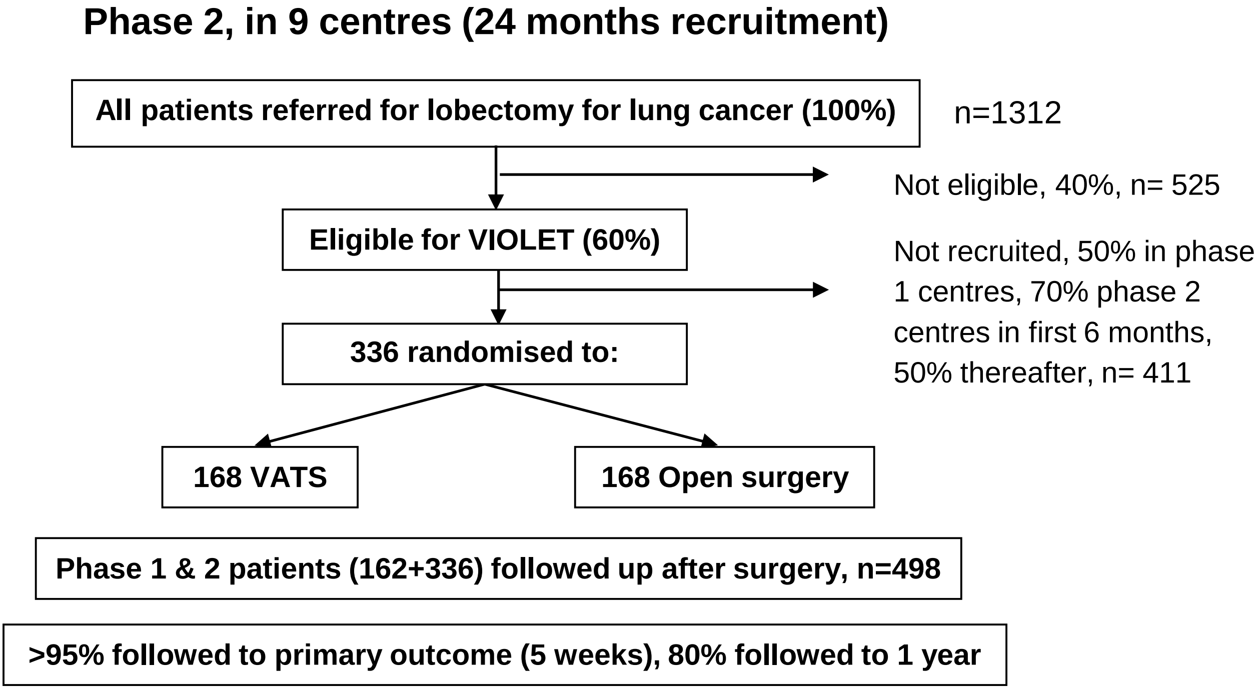

For peer review only - http://bmjopen.bmj.com/site/about/guidelines.xhtml

Figure 2: The trial schema for Phase 2 of the VIOLET study is depicted above

SPIRIT

STANDARD PROTOCOL ITEMS: RECOMMENDATIONS FOR INTERVENTIONAL TRIALS

SPIRIT 2013 Checklist: Recommended items to address in a clinical trial protocol and related documents*

Section/item	Item No	Description	Addressed on page number
**Administrative information**			
Title	1	Descriptive title identifying the study design, population, interventions, and, if applicable, trial acronym	1
Trial registration	2a	Trial identifier and registry name. If not yet registered, name of intended registry	3
	2b	All items from the World Health Organization Trial Registration Data Set	N/A
Protocol version	3	Date and version identifier	20
Funding	4	Sources and types of financial, material, and other support	22
Roles and responsibilities	5a	Names, affiliations, and roles of protocol contributors	21
	5b	Name and contact information for the trial sponsor	20
	5c	Role of study sponsor and funders, if any, in study design; collection, management, analysis, and interpretation of data; writing of the report; and the decision to submit the report for publication, including whether they will have ultimate authority over any of these activities	22
	5d	Composition, roles, and responsibilities of the coordinating centre, steering committee, endpoint adjudication committee, data management team, and other individuals or groups overseeing the trial, if applicable (see Item 21a for data monitoring committee)	N/A

For peer review only - http://bmjopen.bmj.com/site/about/guidelines.xhtml

Introduction

Background and rationale	6a	Description of research question and justification for undertaking the trial, including summary of relevant studies (published and unpublished) examining benefits and harms for each intervention	4, 5
	6b	Explanation for choice of comparators	5
Objectives	7	Specific objectives or hypotheses	5
Trial design	8	Description of trial design including type of trial (eg, parallel group, crossover, factorial, single group), allocation ratio, and framework (eg, superiority, equivalence, noninferiority, exploratory)	5, 6

Methods: Participants, interventions, and outcomes

Study setting	9	Description of study settings (eg, community clinic, academic hospital) and list of countries where data will be collected. Reference to where list of study sites can be obtained	5, 6
Eligibility criteria	10	Inclusion and exclusion criteria for participants. If applicable, eligibility criteria for study centres and individuals who will perform the interventions (eg, surgeons, psychotherapists)	6, 7
Interventions	11a	Interventions for each group with sufficient detail to allow replication, including how and when they will be administered	8, 9
	11b	Criteria for discontinuing or modifying allocated interventions for a given trial participant (eg, drug dose change in response to harms, participant request, or improving/worsening disease)	8-11
	11c	Strategies to improve adherence to intervention protocols, and any procedures for monitoring adherence (eg, drug tablet return, laboratory tests)	11, 12
	11d	Relevant concomitant care and interventions that are permitted or prohibited during the trial	8-11
Outcomes	12	Primary, secondary, and other outcomes, including the specific measurement variable (eg, systolic blood pressure), analysis metric (eg, change from baseline, final value, time to event), method of aggregation (eg, median, proportion), and time point for each outcome. Explanation of the clinical relevance of chosen efficacy and harm outcomes is strongly recommended	9, 10
Participant timeline	13	Time schedule of enrolment, interventions (including any run-ins and washouts), assessments, and visits for participants. A schematic diagram is highly recommended (see Figure)	9, 10

For peer review only - http://bmjopen.bmj.com/site/about/guidelines.xhtml

Sample size	14	Estimated number of participants needed to achieve study objectives and how it was determined, including clinical and statistical assumptions supporting any sample size calculations	13, 14
Recruitment	15	Strategies for achieving adequate participant enrolment to reach target sample size	14-17
**Methods: Assignment of interventions (for controlled trials)**			
Allocation:			
Sequence generation	16a	Method of generating the allocation sequence (eg, computer-generated random numbers), and list of any factors for stratification. To reduce predictability of a random sequence, details of any planned restriction (eg, blocking) should be provided in a separate document that is unavailable to those who enrol participants or assign interventions	7, 8
Allocation concealment mechanism	16b	Mechanism of implementing the allocation sequence (eg, central telephone; sequentially numbered, opaque, sealed envelopes), describing any steps to conceal the sequence until interventions are assigned	7, 8
Implementation	16c	Who will generate the allocation sequence, who will enrol participants, and who will assign participants to interventions	7, 8
Blinding (masking)	17a	Who will be blinded after assignment to interventions (eg, trial participants, care providers, outcome assessors, data analysts), and how	10, 11
	17b	If blinded, circumstances under which unblinding is permissible, and procedure for revealing a participant's allocated intervention during the trial	10, 11
**Methods: Data collection, management, and analysis**			
Data collection methods	18a	Plans for assessment and collection of outcome, baseline, and other trial data, including any related processes to promote data quality (eg, duplicate measurements, training of assessors) and a description of study instruments (eg, questionnaires, laboratory tests) along with their reliability and validity, if known. Reference to where data collection forms can be found, if not in the protocol	9, 10, 13, 14
	18b	Plans to promote participant retention and complete follow-up, including list of any outcome data to be collected for participants who discontinue or deviate from intervention protocols	14-16, 20

Data management	19	Plans for data entry, coding, security, and storage, including any related processes to promote data quality (eg, double data entry; range checks for data values). Reference to where details of data management procedures can be found, if not in the protocol	N/A
Statistical methods	20a	Statistical methods for analysing primary and secondary outcomes. Reference to where other details of the statistical analysis plan can be found, if not in the protocol	17, 18
	20b	Methods for any additional analyses (eg, subgroup and adjusted analyses)	N/A
	20c	Definition of analysis population relating to protocol non-adherence (eg, as randomised analysis), and any statistical methods to handle missing data (eg, multiple imputation)	18
**Methods: Monitoring**			
Data monitoring	21a	Composition of data monitoring committee (DMC); summary of its role and reporting structure; statement of whether it is independent from the sponsor and competing interests; and reference to where further details about its charter can be found, if not in the protocol. Alternatively, an explanation of why a DMC is not needed	27
	21b	Description of any interim analyses and stopping guidelines, including who will have access to these interim results and make the final decision to terminate the trial	N/A
Harms	22	Plans for collecting, assessing, reporting, and managing solicited and spontaneously reported adverse events and other unintended effects of trial interventions or trial conduct	N/A
Auditing	23	Frequency and procedures for auditing trial conduct, if any, and whether the process will be independent from investigators and the sponsor	N/A
**Ethics and dissemination**			
Research ethics approval	24	Plans for seeking research ethics committee/institutional review board (REC/IRB) approval	20, 21
Protocol amendments	25	Plans for communicating important protocol modifications (eg, changes to eligibility criteria, outcomes, analyses) to relevant parties (eg, investigators, REC/IRBs, trial participants, trial registries, journals, regulators)	3, 15

Consent or assent	26a	Who will obtain informed consent or assent from potential trial participants or authorised surrogates, and how (see Item 32)	7, 8
	26b	Additional consent provisions for collection and use of participant data and biological specimens in ancillary studies, if applicable	N/A
Confidentiality	27	How personal information about potential and enrolled participants will be collected, shared, and maintained in order to protect confidentiality before, during, and after the trial	9, 10
Declaration of interests	28	Financial and other competing interests for principal investigators for the overall trial and each study site	20, 22
Access to data	29	Statement of who will have access to the final trial dataset, and disclosure of contractual agreements that limit such access for investigators	19
Ancillary and post-trial care	30	Provisions, if any, for ancillary and post-trial care, and for compensation to those who suffer harm from trial participation	N/A
Dissemination policy	31a	Plans for investigators and sponsor to communicate trial results to participants, healthcare professionals, the public, and other relevant groups (eg, via publication, reporting in results databases, or other data sharing arrangements), including any publication restrictions	3, 15
	31b	Authorship eligibility guidelines and any intended use of professional writers	N/A
	31c	Plans, if any, for granting public access to the full protocol, participant-level dataset, and statistical code	N/A
**Appendices**			
Informed consent materials	32	Model consent form and other related documentation given to participants and authorised surrogates	On request
Biological specimens	33	Plans for collection, laboratory evaluation, and storage of biological specimens for genetic or molecular analysis in the current trial and for future use in ancillary studies, if applicable	N/A

*It is strongly recommended that this checklist be read in conjunction with the SPIRIT 2013 Explanation & Elaboration for important clarification on the items. Amendments to the protocol should be tracked and dated. The SPIRIT checklist is copyrighted by the SPIRIT Group under the Creative Commons "Attribution-NonCommercial-NoDerivs 3.0 Unported" license.

For peer review only - http://bmjopen.bmj.com/site/about/guidelines.xhtml

BMJ Open

Study Protocol for VIdeo assisted thoracoscopic lobectomy versus conventional Open LobEcTomy for lung cancer, a UK multi-centre randomised controlled trial with an internal pilot (The VIOLET study)

Journal:	*BMJ Open*
Manuscript ID	bmjopen-2019-029507.R2
Article Type:	Protocol
Date Submitted by the Author:	17-Jul-2019
Complete List of Authors:	Lim, Eric; The Royal Brompton Hospital, Academic Division of Thoracic Surgery<br>Batchelor, Tim; Bristol Royal Infirmary, Thoracic Surgery<br>Shackcloth, Michael; Liverpool Heart and Chest Hospital,<br>Dunning, Joel ; The James Cook University Hospital, Marton Road<br>McGonigle, Niall; Harefield Hospital, Royal Brompton and Harefield<br>Brush, Tim; University of Bristol<br>Dabner, Lucy ; University of Bristol<br>Harris, Rosie; University of Bristol<br>Mckeon, Holly; University of Bristol<br>Paramasivan, Sangeetha ; University of Bristol<br>Elliott, Daisy; University of Bristol, School of Social and Community Medicine<br>Stokes, Elizabeth; University of Oxford, Health Economics Research Centre Nuffield Department of Population Health<br>Wordsworth, Sarah; University of Oxford, University of Oxford<br>Blazeby, Jane; University of Bristol, Department of Social Medicine<br>Rogers, Chris; University of Bristol, Clinical Sciences
**Primary Subject Heading**:	Surgery
Secondary Subject Heading:	Oncology, Respiratory medicine
Keywords:	Randomised controlled trial, minimally invasive, Cardiothoracic surgery < SURGERY, open surgery, video assisted, lung cancer

SCHOLARONE™
Manuscripts

Study Protocol for VIdeo assisted thoracoscopic lobectomy versus conventional Open LobEcTomy for lung cancer, a UK multi-centre randomised controlled trial with an internal pilot (The VIOLET study)

Eric Lim[1]	e.lim@rbht.nhs.uk
Tim Batchelor[2]	tim.batchelor@uhbristol.nhs.uk
Michael Shackcloth[3]	michael.shackcloth@lhch.nhs.uk
Joel Dunning[4]	joeldunning@nhs.net
Niall McGonigle[5]	Niall.McGonigle@belfasttrust.hscni.net
Tim Brush[6]	tim@ebclinical.co.uk
Lucy Dabner[6]	lucy.dabner@bristol.ac.uk
Rosie Harris[6]	rosie.harris@bristol.ac.uk
Holly McKeon[6]	Holly.mckeon@bristol.ac.uk
Sangeetha Paramasivan[7]	sangeetha.paramasivan@bristol.ac.uk
Daisy Elliott[7]	daisy.elliott@bristol.ac.uk
Elizabeth Stokes[8]	elizabeth.stokes@dph.ox.ac.uk
Sarah Wordsworth[8]	sarah.wordsworth@dph.ox.ac.uk
Jane Blazeby[7]	j.m.blazeby@bristol.ac.uk
Chris A Rogers on behalf of The VIOLET Trialists**[6]	chris.rogers@bristol.ac.uk

1. The Royal Brompton and Harefield NHS Foundation Trust, Sydney Street, London, UK
2. Thoracic Surgery, Bristol Royal Infirmary, University Hospitals Bristol NHS Foundation Trust, Bristol, UK
3. Liverpool Heart and Chest Hospital, Thomas Drive, Liverpool, UK
4. The James Cook University Hospital, Marton Road, Middlesbrough, UK
5. Harefield Hospital, Royal Brompton and Harefield, UK
6. Clinical Trials and Evaluation Unit, Bristol Trials Centre, Bristol Medical School, University of Bristol, Bristol, UK
7. Population Health Sciences, Bristol Medical School, University of Bristol, Bristol, UK
8. Health Economics Research Centre, Nuffield Department of Population Health, University of Oxford, Oxford, UK

For peer review only - http://bmjopen.bmj.com/site/about/guidelines.xhtml

Corresponding author:

Professor Eric Lim, Academic Division of Thoracic Surgery, The Royal Brompton Hospital, Sydney Street, London, UK. Tel: +44 (0)207 351 8591.

Fax: +44 (0)207 351 8560

Email: e.lim@rbht.nhs.uk

Word count: 5485 (excluding title page, abstract, references, figures and tables)

Number of figures: 2

Number of tables: 1

Keywords: Randomised controlled trial; minimally invasive; Cardiothoracic surgery; open surgery; video assisted; lung cancer; lobectomy.

For peer review only

ABSTRACT

Introduction: Lung cancer is a leading cause of cancer deaths worldwide and surgery remains the main treatment for early stage disease. Prior to the introduction of video-assisted thoracoscopic surgery (VATS), lung resection for cancer was undertaken through an open thoracotomy. To date the evidence base supporting the different surgical approaches is based on non-randomised studies, small randomised trials and is focused mainly on short term in- hospital outcomes.

Methods and analysis: The VIOLET study is a UK multicentre parallel group randomised controlled trial (RCT) with blinding of outcome assessors and participants (to hospital discharge) comparing the effectiveness, cost-effectiveness and acceptability of VATS lobectomy versus open lobectomy for treatment of lung cancer. We will test the hypothesis that VATS lobectomy is superior to open lobectomy with respect to self-reported physical function five weeks after randomisation (approximately one month after surgery). Secondary outcomes include assessment of efficacy (hospital stay, pain, proportion and time to uptake of chemotherapy), measures of safety (adverse health events), oncological outcomes (proportion of patients upstaged to pN2 disease and disease-free survival), overall survival, and health related quality of life to 1-year. The QuinteT Recruitment Intervention is integrated into the trial to optimise recruitment.

Ethics and dissemination: This trial has been approved by the UK (Dulwich) National Research Ethics Service Committee London. Findings will be written-up as methodology papers for conference presentation, and publication in peer-reviewed journals. Many aspects of the feasibility work will inform surgical RCTs in general and these will be reported at methodology meetings. We will also link with lung cancer clinical studies groups. The patient and public involvement (PPI) group that works with the Respiratory Biomedical Research Unit at the Brompton Hospital will help identify how we can best publicise the findings.

Trial registration: VIOLET is registered at ISRCTN13472721 (doi 10.1186/ISRCTN13472721)

For peer review only - http://bmjopen.bmj.com/site/about/guidelines.xhtml

Article Summary

Strengths and limitations of this study

- First multicentre randomised trial on this topic
- All surgeons carry out both interventions; the randomisation scheme ensures surgeon balance across the groups to minimise performance bias
- Masking of the incision and evaluation of the success of blinding
- Procedures reflective of UK practice (majority are postero-lateral thoracotomy)
- Surgeon crossovers (i.e. surgeon changes after randomisation) can occur in centres with pooled service provision

INTRODUCTION

Background and objectives

Lung cancer is a leading cause of cancer death worldwide and survival in the United Kingdom (UK) remains amongst the lowest in Europe. Surgery, conventionally undertaken through an open thoracotomy for lung resection, remains the treatment for early stage disease. The randomised trial comparing lobectomy with limited resection (segment or wedge), published in 1995 concluded that lobectomy should be the surgical procedure for patients with lung cancer.(1) The only grade 1 evidence published since is a post-hoc analysis of the CALGB/Alliance 140503 trial in patients with peripheral non-small-cell lung cancer, which concluded that lobar and sublobar resection had similar perioperative mortality and morbidity outcomes.(2) Since the introduction of minimal access video-assisted thoracoscopic surgery (VATS) techniques, lung cancer resection undertaken through a VATS approach increased from 14% in 2010 to 40% in 2014 in the UK.(3)

Much of the evidence generated to date is based on non-randomised studies(4, 5) or small randomised trials focusing on short term (in-hospital) outcomes(6), that are underpowered to detect differences in longer term outcomes such as survival(7) or have focused solely on operative technique.(8) Currently, the most well-designed randomised controlled trial (RCT) by Bendixen et al, reported shorter hospital stay and less pain in patients randomised to VATS lobectomy.(9) In this study, all patients received epidural anaesthesia and anterior thoracotomy for open surgery, which is

For peer review only - http://bmjopen.bmj.com/site/about/guidelines.xhtml

not the current practice for most thoracic surgery centres in the UK. In contrast, a recent trial by Hao et al from China, published in 2018, reported a similar hospital stay in the VATS and axillary thoracotomy groups.(10) In addition, little high quality randomised data has been published to ascertain the cost effectiveness (i.e. quality of life and costs) for VATS, highlighted in a follow up report by Bendixen et al and an on-going trial in France (Lungsco1) that will specifically compare VATS lobectomy versus open thoracotomy from an economic cost to society perspective.(11, 12)

A well designed and conducted RCT comparing the effectiveness and cost-effectiveness of minimal access and open surgery is needed to inform current UK (NHS) practice, health policy and individual surgeon and patient decision-making.

The VIOLET study is a UK multicentre pragmatic RCT comparing the effectiveness, cost-effectiveness and acceptability of VATS lobectomy versus open lobectomy for treatment of lung cancer.

Aims and objectives

The VIOLET study will test the hypothesis that VATS lobectomy is superior to open lobectomy with respect to self-reported physical function five weeks after randomisation (approximately one month after surgery).

Specific objectives are to estimate:

A. The difference between groups in the average self-reported physical function at five weeks.

B. The difference between groups with respect to a range of secondary outcomes including assessment of efficacy (hospital stay, pain, proportion and time to uptake of chemotherapy), measures of safety (adverse health events), oncological outcomes (proportion of patients upstaged to pN2 disease and disease-free survival) and overall survival.

C. The cost effectiveness of VATS lobectomy compared to open lobectomy.

For peer review only

METHODS

Trial design

A UK-based multicentre parallel group RCT with blinding of outcome assessors and participants until hospital discharge after surgery. Figures 1 and 2 show the expected patient pathway for both phases of recruitment to the VIOLET study.

Phase 1: The first phase with an integrated qualitative component is necessary to establish the processes for recruitment and consent. This phase is also essential to develop a study manual and a measure of surgical expertise to proceed to phase 2. Phase 1 will be conducted in five centres; Royal Brompton Hospital in London, The University Hospitals Bristol in Bristol, Liverpool Heart and Chest Hospital in Liverpool, The James Cook University Hospital in Middlesbrough and Harefield Hospital in Harefield. These centres are well spread geographically and represent a mix of university and NHS trusts that are representative of NHS practice. Progression from pilot to the full trial will be dependent on pre-agreed progression criteria (assessed after 18 months of recruitment):

Specifically:

(a) at least 60% of patients undergoing lobectomy are considered eligible for the trial (if necessary, by revising the eligibility criteria);

(b) at least 50% consent to randomisation after 6 months of recruitment;

(c) less than 5% fail to receive their allocated treatment;

(d) less than 5% lost to follow up, excluding deaths;

Phase 2: This phase will extend the study to up to a further five centres. All centres will use the optimum methods of recruitment established in phase 1 and will follow-up all participants to one year.

Study population

Participating *centres* will only be eligible if they meet all the following eligibility criteria: 1. NHS Trust with an established and accredited lung cancer multi-disciplinary team (MDT); 2. Centre carries out ≥40 VATS lobectomies each year and employs at least one surgeon who has carried out ≥ 50 VATS lobectomies.

For peer review only - http://bmjopen.bmj.com/site/about/guidelines.xhtml

Participating *surgeons* will be eligible for the trial if they have performed ≥ 50 VATS lobectomies. Prospective surgeons will be required to submit their activity logs, which will be validated against local audit data from the MDT meetings, prior to acceptance to the trial. Lobectomy via open surgery is currently standard procedure and therefore surgical ability and competence will be assured by Specialist GMC registration.

Patients may enter the study if all the following apply:

1. Adult aged ≥16 years of age
2. Able to give written informed consent, undergoing either
    i. Lobectomy or bilobectomy for treatment of known or suspected primary lung cancer beyond lobar orifice* in TNM8 stage cT1-3 (by size criteria, equivalent to TNM7 stage cT1a-2b) or cT3 (by virtue of 2 nodules in the same lobe), N0-1 and M0 or
    ii. Undergoing frozen section biopsy with the intention to proceed with lobectomy or bilobectomy if primary lung cancer with a peripheral tumour beyond a lobar orifice* in TNM8 stage cT1-3 (by size criteria, equivalent to TNM7 stage cT1a-2b) or cT3 (by virtue of 2 nodules in the same lobe), N0-1 and M0 is confirmed
3. Disease suitable for both minimal access (VATS lobectomy) and lobectomy via open surgery

*In the case of bilobectomy, the distance for the "lobar" orifice is in reference to the bronchus intermedius

Patients may not enter the study if any of the following apply:

1. Previous malignancy that influences life expectancy
2. Pneumonectomy, segmentectomy or non-anatomic resection (e.g. wedge resection) is planned
3. Patient has a serious concomitant disorder that would compromise patient safety during surgery
4. Planned robotic surgery.

For peer review only - http://bmjopen.bmj.com/site/about/guidelines.xhtml

Randomisation

Participants will be randomised in a 1:1 ratio to either VATS lobectomy or open lobectomy. Randomisation will take place through a secure internet-based randomisation system, access to which will be restricted to authorised study personnel. Cohort minimisation (with a random element incorporated) will be used to ensure balance across groups with respect to the surgeon and the allocation will be stratified by centre.

Due to the pragmatic nature of this trial there will inevitably be some variability between surgeons, the surgical teams and the perioperative processes. Such heterogeneity is important as this accurately reflects real clinical practice.

Randomisation will be performed one week prior to the planned operation date, once eligibility has been confirmed and written consent taken by a research nurse. This will allow sufficient time for operating theatre schedules to be arranged. If there is a change in surgeon after randomisation, the analysis will account for the surgeon responsible for performing the operation and not the surgeon originally allocated to the patient.

Trial interventions

All operations will be undertaken with general anaesthesia and with the patient in the lateral decubitus position.

VATS lobectomy is undertaken through one to four keyhole incisions without rib spreading. The use of 'rib spreading' is prohibited as this is the key intra-operative manoeuvre which disrupts tissues and causes pain (and is used in open surgery). The procedure is performed with videoscopic visualisation without direct vision. The hilar structures are dissected, stapled and divided. Endoscopic ligation of pulmonary arterial branches may be performed. The fissure is completed and the lobe of lung resected. The incisions are closed in layers and may involve muscle, fat and skin layers. This definition of VATS lobectomy is a modification of Cancer and Leukaemia Group B (CALGB) 39802.(13)

For peer review only - http://bmjopen.bmj.com/site/about/guidelines.xhtml

For peer review only

Conventional open lobectomy is undertaken through a single incision with or without rib resection and with rib spreading. The operation is performed under direct vision with isolation of the hilar structures (vein, artery and bronchus) which are dissected, ligated and divided in sequence and the lobe of lung resected. The procedures may be undertaken using ligatures, over sewing or with staplers. The thoracotomy is closed in layers starting from pericostal sutures over the ribs, muscle, fat and skin layers.

In both groups, lymph node management is undertaken in accordance with the International Association of the Study of Lung Cancer (IASLC) recommendations where a minimum of six nodes / stations are removed, of which three are from the mediastinum that includes the subcarinal station.(14)

Because this is a pragmatic trial, adaptations and variation in both procedures (with the exception of the mandated elements outlined above) will be permitted although intra-operative details will be collected, and compliance monitored.

Primary and secondary outcomes

The primary outcome is self-reported physical function measured using the European Organization for Research and Treatment of Cancer Quality of Life Questionnaire-C30 (EORTC QLQ-C30) at 5 weeks post-randomisation. Physical function has been chosen because it is a patient-centred outcome that will reflect the anticipated earlier recovery with VATS lobectomy and has been used in other minimal access surgery trials. The primary endpoint has been chosen to be five weeks (one-month post-surgery) to capture the early benefits of minimal access surgery on recovery. The EORTC QLQ-C30 has been validated for use in European cohorts. As well as assessing physical function the questionnaire also assesses psychological and social well-being. Secondary outcomes have been selected to assess the efficacy of the two approaches.

Secondary outcomes are 1. Time from surgery to hospital discharge; 2. Adverse health events; 3. Proportion and time to uptake of adjuvant treatment; 4. Proportion of patients upstaged to pN2 disease after the procedure; 5. Overall and disease-free

For peer review only - http://bmjopen.bmj.com/site/about/guidelines.xhtml

survival to one-year; 6. Proportion of patients who undergo complete resection during the procedure; 7. Proportion of patients who experience prolonged incision pain defined as the need for analgesia > 6 weeks after surgery; 8. Generic and disease-specific Health-related quality of life (HRQoL) assessed using the EORTC QLQ-C30, QLQ-LC13 and EQ-5D-5L questionnaires completed at 2 weeks, 5 weeks, 3 months, 6 months and 1-year post-randomisation); 9. Resource use measured for the duration of post-operative hospital stay until discharge, and at 5 weeks, 3 months, 6 months and one-year post-randomisation.

Data Collection

The schedule of data collection for the study is shown in Table 1. Data will be collected on paper and then entered onto a bespoke database. Access to the database will be via a secure password-protected web-interface (NHS clinical portal). Study data transferred electronically between the University of Bristol and the NHS will only be transferred via a secure NHS.net network in an encrypted form.

Table 1. Data collection for trial participants who agree to randomisation to VATS lobectomy or open lobectomy

	**Pre-randomisation**	**Post-randomisation**								
	**Baseline**	**Day of Surgery**	**Post-op**	**2 days post-op**	**Dis-charge**	**2 weeks***	**5 weeks***	**3 months***	**6 months***	**1 year***
Eligibility	X									
Imaging review (CT / PET-CT¥)	X									
Participant characteristics	X									
Audio recorded consultation	X									
Lobectomy via VATS lobectomy or Open Lobectomy		X								
Intra-operative details		X								
Histopathology staging		X								
Tumour sample for research		X								
Patient Questionnaires										
QLQ-C30	X					X	X	X	X	X
QLQ-LC13	X					X	X	X	X	X
EQ5D	X					X	X	X	X	X

For peer review only - http://bmjopen.bmj.com/site/about/guidelines.xhtml

	Pre-randomisation	Post-randomisation								
	Baseline	Day of Surgery	Post-op	2 days post-op	Dis-charge	2 weeks*	5 weeks*	3 months*	6 months*	1 year*
Bang Blinding Index				X	X					
Pain score	X		X	X						
Adverse Events		X					X	X	X	X
Resource use	X	X					X	X	X	X
CT scan of chest & abdomen										X

*Follow-up time-points will be calculated from the date of randomisation.
¥ Review of images available from staging scans performed in accordance with standard practice at participating centres

Blinding of staff and study participants

The operating surgeon and staff responsible for the care of the participant during the operation cannot be blinded to the participants' treatment allocation. However, in order to minimise the risk of bias, attempts will be made to blind the research nurse responsible for the collection of follow-up data. Specifically, randomisation will be performed by a member of the research team who is not responsible for the collection of follow up data for VIOLET study participants.

Furthermore, efforts will be made to minimise the risk of inadvertent unblinding of the research nurse responsible for data collection during the patient's post-operative stay. To accomplish this, large adhesive dressings will be applied to thorax. These adhesive dressings will be positioned similarly for all participants, regardless of their surgical allocation and will cover both real and potential incision/port locations. The initial adhesive dressings will be applied in the operating theatre by the operating team and these will not be changed until 3 days after surgery (or discharge if discharged before day 3), unless soiling or lack of adherence prompts their premature replacement. Three days after surgery, dressings will be changed by a nurse who is not responsible for conducting the participants' follow-up assessments. Wound cleaning will be performed on all real and potential incision/port locations to promote allocation masking.

Patients who agree to participate in the RCT will not be informed of their treatment allocation until they are discharged from hospital after their operation. In order to ensure that study patients are not unblinded during wound cleaning and dressing

For peer review only

For peer review only - http://bmjopen.bmj.com/site/about/guidelines.xhtml

change, participants will be asked to turn their head away from the wound site that is being tended to. When participants are considered ‘fit-for-discharge’, they will be informed of their treatment allocation and advised as to how best to care for their surgical wounds. Blinding in surgical trials are considered challenging yet an important aspect to reduce bias, patient drop-out and increase the validity of results.(15-17) Participants are made aware at consent that they will not be informed of their treatment allocation until after their surgery. Blinding was approved by the Research Ethics Committee.

The success of blinding will be monitored during each participant’s in-hospital stay. Participants will be asked to complete the Bang-blinding Index(18) at 2 days post-operatively and at discharge, but before the treatment allocation is revealed. The research nurse responsible for data collection and follow-up of VIOLET study participants will also be asked to complete the Bang-blinding Index when the participant is ready for discharge and after the participant attends for their 5 week and 1-year follow-up appointments.

Standardisation of post-operative management

As this is a pragmatic RCT, post-operative care and the criteria for drain removal will be in accordance with local practice. However, we have identified two elements of patient care, which require standardisation to minimise the potential for bias, namely pain-control and the criteria by which a participant’s medical fitness-for-discharge is assessed.

Standardising the use of analgesia across participating centres is impractical and does not reflect the intended pragmatic nature of the trial, it, would also produce data unrepresentative of real clinical practice. Therefore, each participating centre will prescribe analgesia in accordance with their local protocols. All patients recruited to the RCT at that centre will be given the same analgesia regardless of their treatment allocation (i.e. VATS lobectomy or open lobectomy). Local protocols for the provision of analgesia will be defined by the local Principal Investigator (in collaboration with the local research team) prior to the start of recruitment to the RCT. Analgesia administered throughout the participant’s in-hospital stay will be

For peer review only - http://bmjopen.bmj.com/site/about/guidelines.xhtml

recorded on the trial case report forms (CRFs) and compliance with the pre-defined and centre-specific analgesia protocols will be monitored.

In order to objectively compare the time from surgery to hospital discharge between VATS lobectomy and open lobectomy, the following discharge suitability criteria have been developed. Study participants will be evaluated against these criteria to ensure that they are medically fit-for-discharge:

- Participant has achieved satisfactory mobility
- Pain under control with analgesia
- Satisfactory serum haemoglobin and electrolytes (i.e. does not require intervention)
- Satisfactory chest-x-ray (which will be performed as part of routine clinical care)
- No complications that require further / additional treatment

Participants who are considered medically fit-for-discharge may not necessarily be discharged immediately; in some instances, social and other factors may necessitate extended hospitalisation. The time at which participants are considered medically fit-for-discharge and when they are physically discharged from hospital will both be recorded on the trial CRFs.

Sample size calculation

We hypothesise that self-reported physical function (scale 0 – 100, with higher scores indicating better function) five weeks after randomisation for participants undergoing a VATS lobectomy will be superior to the physical function for participants having an open lobectomy, as measured using the EORTC QLQ-C30. The sample size has been chosen to test this hypothesis.

Although the primary endpoint is at 5 weeks post-randomisation self-reported physical function will also be assessed at other time points (baseline, 2 weeks, 3 months, 6 months and 1-year). In estimating the sample size these additional measurements have been taken into account. The power calculation requires the estimation of four parameters, i.e. the effect size that would be considered clinically important, the number of pre and post-surgery measures, and the correlations

For peer review only - http://bmjopen.bmj.com/site/about/guidelines.xhtml

between pre and post-surgery scores and between repeated post-surgery scores. The effect size was chosen based on the published literature,(19) which suggests that an effect size of 0.2 to 0.6 standard deviations equates to a clinically important difference in physical function score of between 5 and 14 points or approximately a one category change in performance status. In the absence of data from which to estimate the correlations between repeated measures we assumed conservative estimates (0.3 between pre and post measures, 0.6 between repeated post measures).

The study size has been set at 398; allowing for a 20% dropout at 1-year, the target sample size is 498 participants. This will provide 90% power to test the hypothesis, assuming an effect size of 0.25 standard deviations in physical function would be clinically important. The calculation based on five post-surgery measures assumes the treatment difference is similar at the five time points.

However, it is anticipated that the difference in physical function may change over time. The calculation based on a single measure shows that the study will have >80% power to detect a difference of 0.25 standard deviations and >90% power to detect a difference of 0.3 standard deviations at the primary endpoint where dropout is expected to be less than 5%.

A study in 498 participants will also have 80% power to detect a 1-day difference in length of hospital stay (i.e. median 3 days versus 4 days, hazard ratio 1.3); assuming 2% of patients do not survive to discharge.

Research procedures

Generic and disease-specific HRQoL measures will assess the profiles of VATS and open lobectomy in the early and mid-postoperative phases. The extensively validated EQ-5D-5L will assess generic aspects of HRQoL and will be used in the economic evaluation.(20, 21) The EORTC QLQ-C30 is one of the most widely used instruments for assessing HRQoL in patients with cancer and the QLQ-LC13 is the lung cancer module with 13 items that assesses lung cancer–specific symptoms.

For peer review only - http://bmjopen.bmj.com/site/about/guidelines.xhtml

Study participants will be asked to complete HRQoL questionnaires at baseline and post-operatively at 2 weeks, 5 weeks, 3 months, 6 months and 1-year post-randomisation. Baseline questionnaires will be administered by the research team at site, whereas the questionnaires completed post-operatively will be administered by the coordinating centre. Participants can choose to receive post-operative questionnaires by post or complete via a secure website.

Patient and Public Involvement (PPI)

The Royal Brompton Hospital Cancer Consortia PPI group were involved from inception and advised on trial design, identification of the choice and timing of the primary outcome, and secondary outcomes that were considered to be important. They were consulted between August 2012 and September 2013. The aim of PPI involvement in VIOLET was to advise on patient-orientated outcomes that matter. The group consists of four patients who have undergone surgery for cancer and one carer. Dr Hall, who is a patient, and a general practitioner by profession, has agreed to sit on the Trial Steering Committee (TSC).

The PPI group will also be involved in reviewing the content and format of PILs and dissemination of the results of the study.

Integrated QuinteT Recruitment Intervention (QRI)

The VIOLET study will employ an integrated QRI to optimise and sustain recruitment throughout the recruitment period because recruitment is anticipated to be difficult. Although recruitment to RCTs is recognised as a research priority,(22) there is a dearth of robust evidence about effective strategies to improve recruitment in RCTs.(23). Surgical RCTs face specific recruitment challenges due to the complex nature of surgical procedures, the dependence on many healthcare professionals across disciplines and surgeon-related factors such as variations in individual practice/expertise.(24) In addition, surgical RCTs, such as VIOLET, that compare minimally invasive and open operations have historically been difficult to conduct and recruit to.(25, 26)

For peer review only

The QRI, employing primarily qualitative research methods can be used to understand recruitment in specific RCTs(27-29) as well as across RCTs.(30-32) It has been shown to optimise recruitment and informed consent, thereby contributing to successful recruitment and trial completion.(33-35) In VIOLET, in order to understand the recruitment process at each centre in real time, investigate the sources of recruitment difficulties and address the challenges, some of the key methods employed(36) will be as follows:

Patient pathway through eligibility and recruitment: A comprehensive process of logging potential trial patients through screening and eligibility phases will be undertaken to provide basic data about the levels of eligibility and recruitment, and identify points at which patients opt in or out of the RCT.

In-depth interviews: In-depth, semi-structured interviews will be conducted and audio-recorded with a purposive sample of staff members involved with aspects of trial design/management and recruitment across centres in phase 1 (and phase 2 where necessary). Patients eligible for recruitment to the RCT may also be interviewed. Across the different groups, interviews will explore participants' perspectives of the trial, the two procedures and acceptability of randomisation between procedures. In addition, recruitment staff (primarily surgeons) interviews will explore their experiences of undertaking both procedures (where appropriate), perceptions of equipoise for themselves and their colleagues, and views on likely outcome of the trial. Interview topic guides will be used to ensure similar topic areas are covered across interviews, while still providing the scope for participants to raise issues of pertinence to them.

Audio recording of recruitment appointments: Face-to-face and telephone consultations of healthcare staff (thoracic surgeons, nurses etc) with potentially eligible patients will be routinely audio recorded across centres to understand the recruitment process at each centre and to identify and investigate the challenges to recruitment. The QRI researcher will listen to and qualitatively analyse the appointments, documenting instances such as unclear, insufficient or imbalanced information provision and unintentional transferring of clinician treatment preferences to patients,

For peer review only - http://bmjopen.bmj.com/site/about/guidelines.xhtml

An account of the anonymised findings from all the data will be fed back to the RCT CI, with a plan of action to optimise recruitment developed collaboratively with key stakeholders. The data will be used by the QRI team to provide supportive and confidential individual and group feedback to recruiters to help them to communicate equipoise, balance treatment options and explain to patients the benefits and purposes of trial participation, whilst optimising informed consent. Feedback sessions will include comparisons between what clinicians think they say to patients (interview data) and what they actually say to patients (consultation data). Rates of recruitment of eligible patients will be closely monitored against the feedback meetings and it is expected that an improvement will be demonstrated in recruitment over time with experience and training for recruiters (as we have demonstrated is possible in other similar trials.(27-29, 33-35))

Economic evaluation

The economic evaluation will compare the costs and effects of VATS lobectomy versus open lobectomy, and will follow established guidelines as set out by the National Institute for Health and Care Excellence(37). The within-trial cost-effectiveness analysis will be undertaken from an NHS and personal social services perspective, with a one-year time horizon from the day of surgery. The primary outcome measure for the economic evaluation will be quality-adjusted life-years (QALYs), estimated using the EuroQol EQ-5D-5L, administered at baseline (pre-randomisation), and five time points post-randomisation (see Table 1). Resource use data collection will be integrated into the trial CRFs for the index admission for items such as duration of surgery, number of staples used, and length of stay; and captured from participants regularly during the one-year follow up (see Table 1) for events such as hospital readmissions, outpatient attendances, and GP or nurse visits in the community.

Statistical analysis plan

The data will be analysed on intention to treat (ITT) and follow CONSORT reporting guidelines (http://www.consort-statement.org/). Randomised participants who are

For peer review only - http://bmjopen.bmj.com/site/about/guidelines.xhtml

not found to have lung cancer will be included in the primary analysis, but a modified ITT analysis excluding these participants will also be performed. Analyses will be adjusted for centre and for design factors included in the cohort minimisation (e.g. the operating surgeon). As the allocation to VATS or open lobectomy is minimised by surgeon, clustering may occur within the dataset. The structure of the data, i.e. nesting of patients by surgeon and centre, will be accounted for in the primary analysis.

Patient reported outcome scores (HRQoL) and will be compared using a mixed regression model, adjusted for baseline measures where appropriate. Changes in treatment effect with time will be assessed by adding a treatment x time interaction to the model and comparing models using a likelihood ratio test. Deaths will be accounted for by modelling HRQoL and survival jointly. Model fit will be assessed and alternative models and / or transformations (e.g. to induce normality) will be explored where appropriate.

Missing items or errors on questionnaire measures will be dealt with according to the scoring manuals or via imputation methods. For other outcomes a complete case analysis will be undertaken if fewer than 5% of cases have missing data, otherwise multiple imputation methods will be considered. Compliance rates will be reported, including the numbers of patients who have withdrawn from the study, have been lost to follow up or died. Causes of death for trial participants will be recorded.

Frequencies of adverse events will be described. Treatment differences will be reported with 95% confidence intervals. In this study of 498 patients we are underpowered to detect differences in survival of less than approximately 20% at 2 years. However, survival rates and 95% confidence intervals will be reported.

One subgroup analysis is planned, comparing pain scores by type of analgesia (paravertebral block vs. intercostal block). This will be tested by adding an analgesia by treatment interaction term to the model. In addition, as an exploratory analysis we will report pain scores within the VATS lobectomy group by number of port sites (single vs multiple port sites), but a formal comparison between the sub-sets of the VATS group is not planned.

For peer review only - http://bmjopen.bmj.com/site/about/guidelines.xhtml

The primary analysis will take place when follow-up is complete for all recruited participants. Interim analysis will be decided in discussion with the Data Monitoring and Safety Committee (DMSC). There is no intention to compare any outcomes between groups after phase 1; the only analyses will be descriptive statistics to summarise recruitment to decide whether the trial satisfies the progression criteria.

Economic Evaluation: For the economic evaluation, unit costs will be derived from nationally published sources and attached to resource use data, and the total costs per participant calculated. Responses to the EQ-5D-5L will be assigned valuations derived from published UK population tariffs(38-40), and combined with survival to calculate QALYs gained per participant. Missing resource use and EQ-5D-5L data will be handled using multiple imputation methods(41). From the average costs and QALYs gained in each trial group, the incremental cost-effectiveness ratio will be derived, producing an incremental cost per QALY gained of VATS lobectomy compared to open lobectomy. Univariate and multivariate sensitivity analyses will assess the impact of varying key parameters in the analysis on baseline cost-effectiveness results. Results will be expressed in terms of a cost-effectiveness acceptability curve, which indicates the likelihood that VATS lobectomy is cost-effective for different levels of willingness to pay for health gain.

Qualitative analysis: Analysis of qualitative data will involve transcribing the audio-recorded consultations, interviews and meetings with consent. The QRI researcher will a) analyse the transcripts and notes thematically using techniques of constant comparison(42) and case study approaches to explore the 'clear obstacles' and 'hidden challenges(30) to recruitment in Violet, and b) employ targeted conversation analysis(27) to focus on areas in the consultations where communication appears to struggle or break down to identify aspects of recruitment that could be improved. Subsets of interview and consultation transcripts will be independently coded by two qualitative researchers, with the coding discussed and any discrepancies resolved, to establish a coding frame that can be applied to other transcripts. Descriptive accounts will summarise key challenges to recruitment. Anonymised findings will be documented and synthesised for presentation to the RCT CI.

For peer review only - http://bmjopen.bmj.com/site/about/guidelines.xhtml

Access to study data: Access to the study data will be limited to authorised personnel. Data will be collected and retained in accordance with the UK Data Protection Act 1998. An anonymised dataset will be held for future research as per the National Institute for Health Research (NIHR) contractual arrangements.

ETHICS

Research ethics approval was granted by the UK (Dulwich) National Research Ethics Service Committee London (reference 14/LO/2129) on 7 January 2015. The trial is managed by the Clinical Trials and Evaluation Unit Bristol (CTEU Bristol) and sponsored by Royal Brompton & Harefield NHS Foundation Trust. Participants have the right to withdraw at any time and if they do withdraw, data collected up until the time of withdrawal will be included in the analyses, unless the participant expresses a wish for their data to be destroyed. Withdrawing patients will be asked at this point if they can be contacted to complete HRQoL questionnaires for an assessment of physical function (primary end point). Participants who choose to withdraw from the study will be treated according to their hospitals' standard procedures.

Changes to the protocol since it was first approved

The number of VATS lobectomies performed for surgeons to be eligible to participate in the VIOLET study was reduced from >50 to >40 to allow more surgeons to participate as there was no evidence to suggest a material difference in outcome. Version 5.0 (dated 13/02/2018) of the protocol is currently in use.

Trial entry criteria by stage were amended following the introduction of the 8th edition of the TNM grading to:

i. Lobectomy or bilobectomy for treatment of known or suspected primary lung cancer beyond lobar orifice* in TNM8 stage cT1-3 (by size criteria, equivalent to TNM7 stage cT1a-2b) or cT3 (by virtue of 2 nodules in the same lobe), N0-1 and M0 or

ii. Undergoing frozen section biopsy with the intention to proceed with lobectomy or bilobectomy if primary lung cancer with a peripheral tumour beyond a lobar orifice* in TNM8 stage cT1-3 (by size criteria, equivalent to TNM7 stage cT1a-2b) or cT3 (by virtue of 2 nodules in the same lobe), N0-1 and M0 is confirmed.

For peer review only - http://bmjopen.bmj.com/site/about/guidelines.xhtml

*In the case of bilobectomy, the distance for the “lobar” orifice is in reference to the bronchus intermedius

The protocol was amended so that the research nurse at the site could obtain questionnaire data during a study visit or telephone call, for those participants who do not return their questionnaire. The relevant regulatory approvals were obtained for amendments to the protocol. Relevant parties (e.g. investigators, trial participants) were informed.

For peer review only

Study status

The actual numbers recruited at 18 months were 160 randomised participants and having received Trial Steering Committee and Funder approval, phase 2 is ongoing and the study is actively recruiting in eight centres. The centres opened in Phase 2 are Heartlands Hospital in Birmingham, John Radcliffe Hospital in Oxford and Castle Hill Hospital in Hull.

The full protocol is available from: https://www.journalslibrary.nihr.ac.uk/programmes/hta/130403/

AUTHOR CONTRIBUTIONS

EL: Study design, preparation and drafting of protocol and manuscript, Chief Investigator for the trial

CAR: Study design, sample size and statistical analysis plan, drafting of protocol and manuscript

JB: Study design, preparation of study protocol and review of manuscript

SP & DE: Design of integrated qualitative study, preparation of study protocol, review of manuscript

ES & SW: Study design, preparation of study protocol, design of health economic component, review of manuscript

TB: Study design, preparation of protocol and review of manuscript

MS: Study design, preparation of protocol and review of manuscript

JD: Preparation of protocol and review of manuscript

NMcG: Preparation of protocol and review of manuscript

For peer review only - http://bmjopen.bmj.com/site/about/guidelines.xhtml

TBr, LD, HM: Preparation of study protocol.

RH: Statistical analysis plan, review of manuscript.

All authors read and approved the final manuscript.

DECLARATION OF INTERESTS

EL and TB report personal fees from Ethicon (Johnson and Johnson), Covidien (Medtronic). There are no other competing interests from the authors.

FUNDING STATEMENT

This project is funded by the NIHR Health Technology Assessment (HTA) Programme (ref 13/04/03). The views expressed are those of the author(s) and not necessarily those of the NHS, the NIHR or the Department of Health and Social Care.

JMB is an NIHR Senior Investigator. JMB, DE and SP are supported by the Medical Research Council (MRC) Hub for Trials Methodology Research ConDuCT-II (Collaboration and Innovation for Difficult Trials in Invasive Procedures) (MR/K025643/1). JMB and DE are also supported by the NIHR Bristol Biomedical Research Centre. The funders had no role in the study design, data collection and analysis, decision to publish or preparation of the manuscript.

CR was supported by the British Heart Foundation (BHF) until April 2016.

This study was designed and delivered in collaboration with the Clinical Trials and Evaluation Unit (CTEU), a UKCRC registered clinical trials unit which, as part of the Bristol Trials Centre, is in receipt of NIHR Clinical Trials Unit (CTU) support funding.

The NIHR, MRC and BHF will not be involved in the study management.

ADDITIONAL FIGURES

Figure 1. The trial schema showing the recruitment pathway for Phase 1 (pilot phase) of the VIOLET study

For peer review only - http://bmjopen.bmj.com/site/about/guidelines.xhtml

Figure 2. The trial schema showing the recruitment pathway for Phase 2 of the VIOLET study

LIST OF ABBREVIATIONS

BHF	British Heart Foundation
CALGB	Cancer and Leukemia Group B
CI	Chief Investigator
CONSORT	Consolidated Standards of Reporting Trials
ConDuCT-II	Collaboration and Innovation for Difficult Trials in Invasive Procedures
CTEU	Clinical Trials and Evaluation Unit
DMSC	Data monitoring and safety committee
EORTC QLQ-C30	European Organization for Research and Treatment of Cancer Quality of Life Questionnaire-C30
EORTC QLQ-LC13	European Organisation for Research and Treatment of Cancer Quality of Life Questionnaire Lung Cancer 13
EQ-5D	EuroQoL-5D
GMC	General Medical Council
HRQoL	Health related quality of life
HTA	Health Technology Assessment
MDT	Multi-Disciplinary Team
MRC	Medical Research Council
NIHR	National Institute for Health Research
PIL	Patient information leaflet
PPI	Patient and Public Involvement
QALY	Quality-adjusted life year
QRI	QuinteT Recruitment Intervention
RCT	Randomised controlled trial
REC	Research ethics committee
TSC	Trial Steering Committee
TMG	Trial Management Group
TNM	TNM Classification of Malignant Tumours
UKCRC	The UK Clinical Research Collaboration
VATS	Video-assisted thoracoscopic surgery

For peer review only

ACKNOWLEDGEMENTS

The VIOLET trial is sponsored by The Royal Brompton and Harefield NHS Foundation Trust. The sponsor will be responsible for the oversight of the VIOLET study and to ensure the trial is managed appropriately.

VIOLET is supported by the UK Thoracic Surgery Research Collaborative

For peer review only - http://bmjopen.bmj.com/site/about/guidelines.xhtml

**VIOLET Trialists

Project management team members

Professor Eric Lim, Chief Investigator

Professor Chris Rogers, Methodological lead and Statistician

Tim Brush, Clinical Trial Coordinator

Lucy Dabner, Clinical Trial Coordinator

Dawn Phillips, Clinical Trial Coordinator

Holly Mckeon, Clinical Trial Coordinator

Chloe Beard, Assistant Trial Coordinator

Rosie Harris, Medical Statistician

Dr Daisy Elliott, Senior Research Associate, QuinteT Research Intervention

Dr Sangeetha Paramasivan, Senior Research Associate, QuinteT Research Intervention

Dr Alba Realpe Rojas, Senior Research Associate, QuinteT Research Intervention

Dr Sarah Wordsworth, Lead Health Economist

Dr Elizabeth Stokes, Health Economist

Professor Jane Blazeby, Methodologist and Surgeon

Professor Andrew G Nicholson, Pathologist

<u>Participating sites members phase 1</u>

Royal Brompton Hospital:

Professor Eric Lim, Principal Investigator, Consultant Thoracic Surgeon

Miss Sofina Begum, Consultant Thoracic Surgeon

Mr Simon Jordan, Consultant Thoracic Surgeon

Paulo De Sousa, Senior Research Nurse

Monica Tavares Barbosa, Research Nurse

Bristol Royal Infirmary:

Mr Tim Batchelor, Principal Investigator, Consultant Thoracic Surgeon

Ms Eveline Internullo, Consultant Thoracic Surgeon

Mr Rakesh Krishnadas, Consultant Thoracic Surgeon

Mr Gianluca Casali, Consultant Thoracic Surgeon

For peer review only - http://bmjopen.bmj.com/site/about/guidelines.xhtml

Mr Doug West, Consultant Thoracic Surgeon

Karen Bobruk, Research Nurse

Catherine O'Donovan, Research Nurse

Louise Flintoff, Research Nurse

Amelia Lowe, Trial Coordinator

Joanna Nicklin, Research Nurse

Emma Heron, Research Nurse

Jo Chambers, Research Nurse

Becky Houlihan, Research Nurse

Laura Beacham, Research Nurse

Heather Hudson, Research Nurse

Katy Tucker, Trial Coordinator

Toni Farmery, Trial Coordinator

Danielle Davis, Trial Coordinator

Liverpool Heart and Chest Hospital:

Mr Mike Shackcloth, Principal Investigator, Consultant Thoracic Surgeon

Mr Julius Asante-Siaw, Consultant Thoracic Surgeon

Ms Susannah Love, Consultant Thoracic Surgeon

Sarah Feeney, Research Nurse

Lindsey Murphy, Research Nurse

Almudena Duran Rosas, Research Nurse

Andrea Young, Research Nurse

James Cook Hospital:

Mr Joel Dunning, Principal Investigator, Consultant Thoracic Surgeon

Mr Ian Paul, Consultant Thoracic Surgeon

Hyder Latif, Clinical Trial Coordinator

Charlotte Jacobs, Clinical Trial Coordinator

Alison Chilvers, Clinical Trial Coordinator

Edward Stephenson, Research Data Assistant

Martyn Cain, Research Data Assistant

Nazalie Iqbal, Research Data Assistant

For peer review only - http://bmjopen.bmj.com/site/about/guidelines.xhtml

Harefield Hospital:

Mr Vladimir Anikin, Principal Investigator, Consultant Thoracic Surgeon

Mr Niall McGonigle, previous Principal Investigator, Consultant Thoracic Surgeon

Claire Prendergast, Research Nurse

Lisa Jones, Research Nurse

Paula Rogers, Research Nurse Manager

Participating sites members phase 2

Birmingham Heartlands Hospital:

Mr Babu Naidu, Principal Investigator, Consultant Thoracic Surgeon

Mr Hazem Fallouh, Consultant Thoracic Surgeon

Mr Luis Hernandez, Consultant Thoracic Surgeon

Mr Maninder Kalkat, Consultant Thoracic Surgeon

Mr Richard Steyn, Consultant Thoracic Surgeon

Nicola Oswald, Thoracic Research Fellow

Amy Kerr, Senior Research Nurse

Charlotte Ferris, Research Nurse

Jo Webb, Research Nurse

Joanne Taylor, Research Nurse

Hollie Bancroft, R&D Biomedical scientist

Salma Kadiri, Research Practitioner

Zara Jalal Senior, Thoracic Research Data Manager

Oxford University Hospitals NHS Foundation Trust:

Miss Elizabeth Belcher, Principal Investigator, Consultant Thoracic Surgeon

Mr Dionisios Stavroulias, Consultant Thoracic Surgeon

Mr Francesco Di Chiara, Consultant Thoracic Surgeon

Kathryn Saunders, Research Nurse

May Havinden-Williams, Research Nurse

Mark Ainsworth, Research Nurse

Castle Hill Hospital:

Professor Mahmoud Loubani, Principal Investigator, Consultant Cardiothoracic Surgeon

For peer review only

Mr Syed Qadri, Consultant Thoracic Surgeon

Karen Dobbs, Research Nurse

Paul Atkin, Research Nurse

Dominic Fellowes, Research Nurse

Leanne Cox, Clinical Trials Assistant

Edinburgh Royal Infirmary:

Mr Vipin Zamvar, Principal Investigator, Consultant Cardiothoracic Surgeon

Lucy Marshall, Research Nurse

Fiona Strachan, Research Nurse Manager

Stacey Stewart, Research Nurse

Independent Trial Steering Committee members

Professor Ruth Langley (Chair), Professor of Oncology and Clinical Trials

Professor Joy Adamson, Professor of Applied Health Research & Ageing

Mr Ian Hunt, Consultant Thoracic Surgeon

Professor Peter Licht, Professor of Cardiothoracic Surgery

Dr Arjun Nair, Consultant Radiologist

Mr Chris Hall, Patient representative

Mr Mike Cowen, Consultant Cardiothoracic Surgeon (from study start to Jan 2017)

Independent Data Monitoring and Safety Committee members

Ms Susan J Dutton (Chair since May 2017, previously member of the Committee), University Research Lecturer and Oxford Clinical Trials Research Unit (OCTRU) Lead Statistician

Mr Alan Kirk, Consultant Thoracic Surgeon

Professor Keith Kerr, Professor of Pulmonary Pathology

Mr Rajesh Shah, Consultant Thoracic Surgeon

Dr Nagmi Qureshi, Consultant Radiologist

Professor Tom Treasure, Professor of Cardiothoracic Surgery (Chair from study start to March 2017)

For peer review only

REFERENCES

1. Ginsberg RJ, Rubinstein LV. Randomized trial of lobectomy versus limited resection for T1 N0 non-small cell lung cancer. Lung Cancer Study Group. Ann Thorac Surg. 1995;60(3):615-22; discussion 22-3.
2. Altorki NK, Wang X, Wigle D, et al. Perioperative mortality and morbidity after sublobar versus lobar resection for early-stage non-small-cell lung cancer: post-hoc analysis of an international, randomised, phase 3 trial (CALGB/Alliance 140503). Lancet Respir Med. 2018;6(12):915-24.
3. The SCTS Thoracic Surgery Audit Group. Society for Cardiothoracic Surgery in Great Britain and Ireland. The Thoracic Surgery Registry Brief Report Audit Years 2011-12 to 2013-14 [Available from: https://scts.org/_userfiles/pages/file/Audit%20and%20Outcomes/3_year_data_summary_2015[1].pdf.
4. Yan TD, Black D, Bannon PG, et al. Systematic review and meta-analysis of randomized and nonrandomized trials on safety and efficacy of video-assisted thoracic surgery lobectomy for early-stage non-small-cell lung cancer. J Clin Oncol. 2009;27(15):2553-62.
5. Cao C, Manganas C, Ang SC, et al. A meta-analysis of unmatched and matched patients comparing video-assisted thoracoscopic lobectomy and conventional open lobectomy. Ann Cardiothorac Surg. 2012;1(1):16-23.
6. Kirby TJ, Mack MJ, Landreneau RJ, et al. Lobectomy--video-assisted thoracic surgery versus muscle-sparing thoracotomy. A randomized trial. J Thorac Cardiovasc Surg. 1995;109(5):997-1001; discussion -2.
7. Sugi K, Kaneda Y, Esato K. Video-assisted thoracoscopic lobectomy achieves a satisfactory long-term prognosis in patients with clinical stage IA lung cancer. World J Surg. 2000;24(1):27-30.
8. Sugiura H, Morikawa T, Kaji M, et al. Long-term benefits for the quality of life after video-assisted thoracoscopic lobectomy in patients with lung cancer. Surg Laparosc Endosc Percutan Tech. 1999;9(6):403-8.
9. Bendixen M, Jorgensen OD, Kronborg C, et al. Postoperative pain and quality of life after lobectomy via video-assisted thoracoscopic surgery or anterolateral thoracotomy for early stage lung cancer: a randomised controlled trial. Lancet Oncol. 2016;17(6):836-44.
10. Long H, Tan Q, Luo Q, et al. Thoracoscopic Surgery Versus Thoracotomy for Lung Cancer: Short-Term Outcomes of a Randomized Trial. Ann Thorac Surg. 2018;105(2):386-92.
11. Bendixen M, Kronborg C, Jorgensen OD, et al. Cost-utility analysis of minimally invasive surgery for lung cancer: a randomized controlled trial. Eur J Cardiothorac Surg. 2019.
12. Pagès PB, Abou Hanna H, Bertaux AC, et al. Medicoeconomic analysis of lobectomy using thoracoscopy versus thoracotomy for lung cancer: a study protocol for a multicentre randomised controlled trial (Lungsco01). BMJ Open. 2017;7(6):e012963.
13. Swanson SJ, Herndon JE, 2nd, D'Amico TA, et al. Video-assisted thoracic surgery lobectomy: report of CALGB 39802--a prospective, multi-institution feasibility study. J Clin Oncol. 2007;25(31):4993-7.
14. Goldstraw P. International Association for the Study of Lung Cancer Staging Manual in Thoracic Oncology. Florida, USA: Editorial Rx Press; 2009.

For peer review only - http://bmjopen.bmj.com/site/about/guidelines.xhtml

15. Speich B. Blinding in Surgical Randomized Clinical Trials in 2015. Ann Surg. 2017;266(1):21-2.
16. Wartolowska K, Beard D, Carr A. Blinding in trials of interventional procedures is possible and worthwhile. F1000Res. 2017;6:1663.
17. Karanicolas PJ, Farrokhyar F, Bhandari M. Practical tips for surgical research: blinding: who, what, when, why, how? Can J Surg. 2010;53(5):345-8.
18. Bang H, Ni L, Davis CE. Assessment of blinding in clinical trials. Control Clin Trials. 2004;25(2):143-56.
19. Maringwa JT, Quinten C, King M, et al. Minimal important differences for interpreting health-related quality of life scores from the EORTC QLQ-C30 in lung cancer patients participating in randomized controlled trials. Support Care Cancer. 2011;19(11):1753-60.
20. EuroQol Group. EuroQol - a new facility for the measurement of health-related quality of life. Health policy. 1990;16(3):199-208.
21. Herdman M, Gudex C, Lloyd A, et al. Development and preliminary testing of the new five-level version of EQ-5D (EQ-5D-5L). Quality of life research : an international journal of quality of life aspects of treatment, care and rehabilitation. 2011;20(10):1727-36.
22. Healy P, Galvin S, Williamson PR, et al. Identifying trial recruitment uncertainties using a James Lind Alliance Priority Setting Partnership - the PRioRiTy (Prioritising Recruitment in Randomised Trials) study. Trials. 2018;19(1):147.
23. Treweek S, Mitchell E, Pitkethly M, et al. Strategies to improve recruitment to randomised controlled trials. Cochrane Database of Systematic Reviews. 2010(1).
24. Ergina PL, Cook JA, Blazeby JM, et al. Challenges in evaluating surgical innovation. Lancet. 2009;374(9695):1097-104.
25. Frampton SK, R.L. John Wickham's New Surgery: 'Minimally Invasive Therapy', Innovation, and Approaches to Medical Practice in Twentieth-century Britain. Soc Hist Med. 2017;30(3):544–66.
26. Schlich TT, C.L. Patient choice and the history of minimally invasive surgery. Lancet. 2016;388:1369-70.
27. Donovan JL, Lane JA, Peters TJ, et al. Development of a complex intervention improved randomization and informed consent in a randomized controlled trial. J Clin Epidemiol. 2009;62(1):29-36.
28. Paramasivan S, Rogers CA, Welbourn R, et al. Enabling recruitment success in bariatric surgical trials: pilot phase of the By-Band-Sleeve study. Int J Obes (Lond). 2017;41(11):1654-61.
29. Stein RC, Dunn JA, Bartlett JM, et al. OPTIMA prelim: a randomised feasibility study of personalised care in the treatment of women with early breast cancer. Health Technol Assess. 2016;20(10):xxiii-xxix, 1-201.
30. Donovan JL, Paramasivan S, de Salis I, et al. Clear obstacles and hidden challenges: understanding recruiter perspectives in six pragmatic randomised controlled trials. Trials. 2014;15:5.
31. Mills N, Donovan JL, Wade J, et al. Exploring treatment preferences facilitated recruitment to randomized controlled trials. J Clin Epidemiol. 2011;64(10):1127-36.
32. Rooshenas L, Elliott D, Wade J, et al. Conveying Equipoise during Recruitment for Clinical Trials: Qualitative Synthesis of Clinicians' Practices across Six Randomised Controlled Trials. PLoS Med. 2016;13(10):e1002147.
33. Beard DJ, Rees JL, Cook JA, et al. Arthroscopic subacromial decompression for subacromial shoulder pain (CSAW): a multicentre, pragmatic, parallel group,

For peer review only - http://bmjopen.bmj.com/site/about/guidelines.xhtml

placebo-controlled, three-group, randomised surgical trial. Lancet. 2017;391(10118):329-38.
34. Hamdy FC, Donovan JL, Lane JA, et al. 10-Year Outcomes after Monitoring, Surgery, or Radiotherapy for Localized Prostate Cancer. N Engl J Med. 2016;375(15):1415-24.
35. Rooshenas L SL, Blazeby JM, Rogers CA, Tilling KM, Husbands S, et al. The Quintet Recruitment Intervention supported five randomized trials to recruit to target: a mixed-methods evaluation. Journal of Clinical Epidemiology 2019(106):108-20.
36. Donovan JL, Rooshenas L, Jepson M, et al. Optimising recruitment and informed consent in randomised controlled trials: the development and implementation of the Quintet Recruitment Intervention (QRI). Trials. 2016;17(1):283.
37. National Institute for Health and Care Excellence. Guide to the Methods of Technology Appraisal 2013. London: National Institute for Health and Care Excellence; 2013.
38. Dolan P. Modeling valuations for EuroQol health states. Med Care. 1997;35(11):1095-108.
39. National Institute for Health and Care Excellence. Position statement on use of the EQ-5D-5L valuation set for England (updated November 2018)2018 09 January 2019. Available from: https://www.nice.org.uk/Media/Default/About/what-we-do/NICE-guidance/NICE-technology-appraisal-guidance/eq5d5l_nice_position_statement.pdf.
40. van Hout B, Janssen MF, Feng YS, et al. Interim scoring for the EQ-5D-5L: mapping the EQ-5D-5L to EQ-5D-3L value sets. Value in health : the journal of the International Society for Pharmacoeconomics and Outcomes Research. 2012;15(5):708-15.
41. White IR, Royston P, Wood AM. Multiple imputation using chained equations: Issues and guidance for practice. Statistics in medicine. 2011;30(4):377-99.
42. Glaser BGS, A.L. The Discovery of Grounded Theory: Strategies for Qualitative Research. Piscataway, NJ, USA: Transaction Publishers; 2009.

For peer review only

For peer review only - http://bmjopen.bmj.com/site/about/guidelines.xhtml

Phase 1, in 5 centres (21 months recruitment)

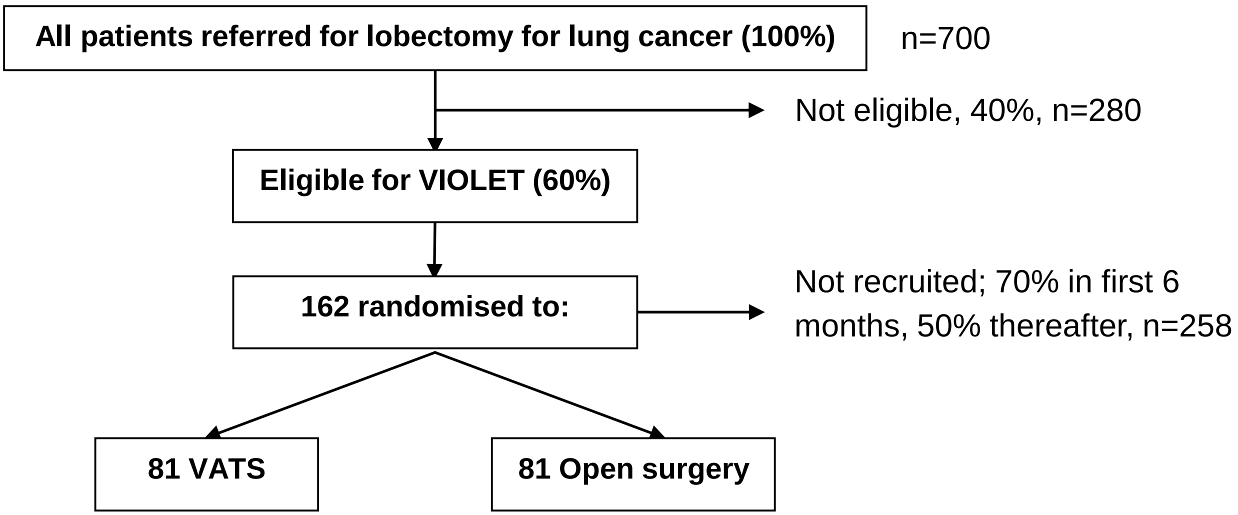

Figure 1: The trial schema for Phase 1 (pilot phase) of the VIOLET study is depicted above

For peer review only - http://bmjopen.bmj.com/site/about/guidelines.xhtml

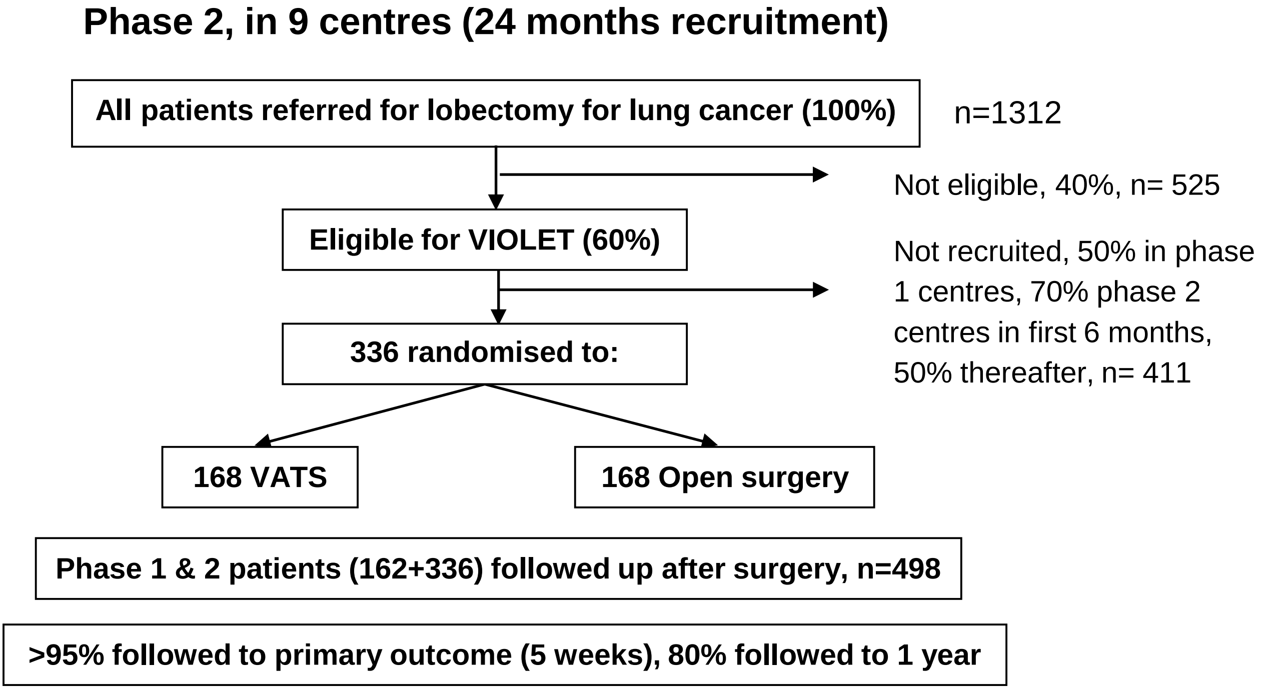

For peer review only - http://bmjopen.bmj.com/site/about/guidelines.xhtml

Figure 2: The trial schema for Phase 2 of the VIOLET study is depicted above

STANDARD PROTOCOL ITEMS: RECOMMENDATIONS FOR INTERVENTIONAL TRIALS

SPIRIT 2013 Checklist: Recommended items to address in a clinical trial protocol and related documents*

Section/item	Item No	Description	Addressed on page number
**Administrative information**			
Title	1	Descriptive title identifying the study design, population, interventions, and, if applicable, trial acronym	1
Trial registration	2a	Trial identifier and registry name. If not yet registered, name of intended registry	3
	2b	All items from the World Health Organization Trial Registration Data Set	N/A
Protocol version	3	Date and version identifier	20
Funding	4	Sources and types of financial, material, and other support	22
Roles and responsibilities	5a	Names, affiliations, and roles of protocol contributors	21
	5b	Name and contact information for the trial sponsor	20
	5c	Role of study sponsor and funders, if any, in study design; collection, management, analysis, and interpretation of data; writing of the report; and the decision to submit the report for publication, including whether they will have ultimate authority over any of these activities	22
	5d	Composition, roles, and responsibilities of the coordinating centre, steering committee, endpoint adjudication committee, data management team, and other individuals or groups overseeing the trial, if applicable (see Item 21a for data monitoring committee)	N/A

For peer review only

Introduction

Background and rationale	6a	Description of research question and justification for undertaking the trial, including summary of relevant studies (published and unpublished) examining benefits and harms for each intervention	4, 5
	6b	Explanation for choice of comparators	5
Objectives	7	Specific objectives or hypotheses	5
Trial design	8	Description of trial design including type of trial (eg, parallel group, crossover, factorial, single group), allocation ratio, and framework (eg, superiority, equivalence, noninferiority, exploratory)	5, 6

Methods: Participants, interventions, and outcomes

Study setting	9	Description of study settings (eg, community clinic, academic hospital) and list of countries where data will be collected. Reference to where list of study sites can be obtained	5, 6
Eligibility criteria	10	Inclusion and exclusion criteria for participants. If applicable, eligibility criteria for study centres and individuals who will perform the interventions (eg, surgeons, psychotherapists)	6, 7
Interventions	11a	Interventions for each group with sufficient detail to allow replication, including how and when they will be administered	8, 9
	11b	Criteria for discontinuing or modifying allocated interventions for a given trial participant (eg, drug dose change in response to harms, participant request, or improving/worsening disease)	8-11
	11c	Strategies to improve adherence to intervention protocols, and any procedures for monitoring adherence (eg, drug tablet return, laboratory tests)	11, 12
	11d	Relevant concomitant care and interventions that are permitted or prohibited during the trial	8-11
Outcomes	12	Primary, secondary, and other outcomes, including the specific measurement variable (eg, systolic blood pressure), analysis metric (eg, change from baseline, final value, time to event), method of aggregation (eg, median, proportion), and time point for each outcome. Explanation of the clinical relevance of chosen efficacy and harm outcomes is strongly recommended	9, 10
Participant timeline	13	Time schedule of enrolment, interventions (including any run-ins and washouts), assessments, and visits for participants. A schematic diagram is highly recommended (see Figure)	9, 10

For peer review only - http://bmjopen.bmj.com/site/about/guidelines.xhtml

Sample size	14	Estimated number of participants needed to achieve study objectives and how it was determined, including clinical and statistical assumptions supporting any sample size calculations	13, 14
Recruitment	15	Strategies for achieving adequate participant enrolment to reach target sample size	14-17
**Methods: Assignment of interventions (for controlled trials)**			
Allocation:			
Sequence generation	16a	Method of generating the allocation sequence (eg, computer-generated random numbers), and list of any factors for stratification. To reduce predictability of a random sequence, details of any planned restriction (eg, blocking) should be provided in a separate document that is unavailable to those who enrol participants or assign interventions	7, 8
Allocation concealment mechanism	16b	Mechanism of implementing the allocation sequence (eg, central telephone; sequentially numbered, opaque, sealed envelopes), describing any steps to conceal the sequence until interventions are assigned	7, 8
Implementation	16c	Who will generate the allocation sequence, who will enrol participants, and who will assign participants to interventions	7, 8
Blinding (masking)	17a	Who will be blinded after assignment to interventions (eg, trial participants, care providers, outcome assessors, data analysts), and how	10, 11
	17b	If blinded, circumstances under which unblinding is permissible, and procedure for revealing a participant's allocated intervention during the trial	10, 11
**Methods: Data collection, management, and analysis**			
Data collection methods	18a	Plans for assessment and collection of outcome, baseline, and other trial data, including any related processes to promote data quality (eg, duplicate measurements, training of assessors) and a description of study instruments (eg, questionnaires, laboratory tests) along with their reliability and validity, if known. Reference to where data collection forms can be found, if not in the protocol	9, 10, 13, 14
	18b	Plans to promote participant retention and complete follow-up, including list of any outcome data to be collected for participants who discontinue or deviate from intervention protocols	14-16, 20

Data management	19	Plans for data entry, coding, security, and storage, including any related processes to promote data quality (eg, double data entry; range checks for data values). Reference to where details of data management procedures can be found, if not in the protocol	N/A
Statistical methods	20a	Statistical methods for analysing primary and secondary outcomes. Reference to where other details of the statistical analysis plan can be found, if not in the protocol	17, 18
	20b	Methods for any additional analyses (eg, subgroup and adjusted analyses)	N/A
	20c	Definition of analysis population relating to protocol non-adherence (eg, as randomised analysis), and any statistical methods to handle missing data (eg, multiple imputation)	18
**Methods: Monitoring**			
Data monitoring	21a	Composition of data monitoring committee (DMC); summary of its role and reporting structure; statement of whether it is independent from the sponsor and competing interests; and reference to where further details about its charter can be found, if not in the protocol. Alternatively, an explanation of why a DMC is not needed	27
	21b	Description of any interim analyses and stopping guidelines, including who will have access to these interim results and make the final decision to terminate the trial	N/A
Harms	22	Plans for collecting, assessing, reporting, and managing solicited and spontaneously reported adverse events and other unintended effects of trial interventions or trial conduct	N/A
Auditing	23	Frequency and procedures for auditing trial conduct, if any, and whether the process will be independent from investigators and the sponsor	N/A
**Ethics and dissemination**			
Research ethics approval	24	Plans for seeking research ethics committee/institutional review board (REC/IRB) approval	20, 21
Protocol amendments	25	Plans for communicating important protocol modifications (eg, changes to eligibility criteria, outcomes, analyses) to relevant parties (eg, investigators, REC/IRBs, trial participants, trial registries, journals, regulators)	3, 15

For peer review only - http://bmjopen.bmj.com/site/about/guidelines.xhtml

Consent or assent	26a	Who will obtain informed consent or assent from potential trial participants or authorised surrogates, and how (see Item 32)	7, 8
	26b	Additional consent provisions for collection and use of participant data and biological specimens in ancillary studies, if applicable	N/A
Confidentiality	27	How personal information about potential and enrolled participants will be collected, shared, and maintained in order to protect confidentiality before, during, and after the trial	9, 10
Declaration of interests	28	Financial and other competing interests for principal investigators for the overall trial and each study site	20, 22
Access to data	29	Statement of who will have access to the final trial dataset, and disclosure of contractual agreements that limit such access for investigators	19
Ancillary and post-trial care	30	Provisions, if any, for ancillary and post-trial care, and for compensation to those who suffer harm from trial participation	N/A
Dissemination policy	31a	Plans for investigators and sponsor to communicate trial results to participants, healthcare professionals, the public, and other relevant groups (eg, via publication, reporting in results databases, or other data sharing arrangements), including any publication restrictions	3, 15
	31b	Authorship eligibility guidelines and any intended use of professional writers	N/A
	31c	Plans, if any, for granting public access to the full protocol, participant-level dataset, and statistical code	N/A
**Appendices**			
Informed consent materials	32	Model consent form and other related documentation given to participants and authorised surrogates	On request
Biological specimens	33	Plans for collection, laboratory evaluation, and storage of biological specimens for genetic or molecular analysis in the current trial and for future use in ancillary studies, if applicable	N/A

*It is strongly recommended that this checklist be read in conjunction with the SPIRIT 2013 Explanation & Elaboration for important clarification on the items. Amendments to the protocol should be tracked and dated. The SPIRIT checklist is copyrighted by the SPIRIT Group under the Creative Commons "Attribution-NonCommercial-NoDerivs 3.0 Unported" license.
